# Stability of Partially Congested Travelling Wave Solutions for the Dissipative Aw-Rascle System

**DOI:** 10.1007/s00021-025-00988-2

**Published:** 2025-12-18

**Authors:** É Deléage, Muhammed Ali Mehmood

**Affiliations:** 1 Aix Marseille Univ., CNRS, Centrale Marseille, I2M UMR CNRS 7373, 13000 Marseille, France; 2 Univ. Grenoble Alpes, CNRS, INRAE, IRD, Grenoble INP, IGE, 38000 Grenoble, France; 3 Imperial College London, London, United Kingdom

**Keywords:** Aw-Rascle system, Compressible Navier-Stokes equations, Singular limit, Viscous shock waves, Nonlinear stability, 35Q35, 35L67, 35B25, 76T20

## Abstract

We prove the non-linear stability of a class of travelling-wave solutions to the dissipative Aw-Rascle system with a singular offset function, which is formally equivalent to the compressible pressureless Navier-Stokes system with a singular viscosity. These solutions encode the effect of congestion by connecting a congested left state to an uncongested right state, and may also be viewed as approximations of solutions to the ‘hard-congestion model’. By using carefully weighted energy estimates we are able to prove the non-linear stability of viscous shock waves to the Aw-Rascle system under a small zero integral perturbation, which in particular extends previous results that do not handle the case where the viscosity is singular.

## Introduction

### Presentation of the Model

We study the following generalised Aw-Rascle system on the real line:

1 $$\begin{aligned} \left\{ \begin{aligned}&\partial _t \rho +\partial _y(\rho u)=0,\\&\partial _t(\rho w)+\partial _y (\rho u w)=0. \end{aligned} \right. \end{aligned}$$

Here, the quantity $\rho$ represents the density while *u* and *w* respectively refer to the actual and desired velocities of agents. This system was originally coined in 2000 by Aw and Rascle [2] and has popularly been used to model the evolution of a system of interacting agents, such as the flow of vehicular traffic [10, 19, 22] or crowd dynamics [1]. The standard Aw-Rascle system is complemented by the relation $w = u + P(\rho )$, where $P=P(\rho )$ is known as the ‘offset’ function. In this paper, we consider the case where $w = u+\partial _y p_\epsilon (\rho )$, with $p_\epsilon$ being a singular function of the density depending on a parameter $\epsilon$. More precisely, for $\epsilon >0$ fixed, we take

2 $$\begin{aligned} w= u+ \partial _{y}p_\epsilon (\rho ) =u+ \partial _{y} \left( \epsilon \left( \frac{\rho }{1-\rho }\right) ^\gamma \right) ,\qquad \gamma \ge 1. \end{aligned}$$

Note that the singularity as $\rho$ approaches 1 in (2) is physically significant, since it implies that the density of agents within the system may not exceed a maximal packing constraint, i.e. $\rho \le 1$. In one-dimension it is interesting to note that the system may be formally rewritten as

3 $$\begin{aligned} \left\{ \begin{aligned}&\partial _t \rho +\partial _y(\rho u)=0,\\&\partial _t(\rho u)+\partial _y(\rho u^2)-\partial _y(\rho ^2p_\epsilon '(\rho )\partial _y u)=0, \end{aligned} \right. \end{aligned}$$

which resembles the one-dimensional compressible pressureless Navier-Stokes equations with a singular degenerate viscosity coefficient $\lambda _\epsilon := \rho ^2p_\epsilon '(\rho )$.

The system (3) with (2) was rigorously derived by Lefebvre-Lepot and Maury in [18] from a microscopic lubrication model, and describes the evolution of particles suspended in a viscous fluid that interact with each other via a lubricating force. The viscosity coefficient obtained by Lefebvre-Lepot and Maury is $\epsilon (1-\rho )^{-1}$, where $\epsilon$ is the viscosity of the interstitial fluid. The case $\gamma = 1$ has also proven to be physically relevant in applications. Indeed, the viscosity coefficient of dense granular suspensions was experimentally measured to behave like $(\phi _c-\phi )^{-2}$ as the solid volume fraction $\phi$ (which corresponds to the non-dimensionalised density) approaches the maximal volume fraction $\phi _c$ (see for instance [15] for a review).

Let us now go back to the momentum equation of (3). In the region where $\rho$ is far from 1, the viscosity $\lambda _\epsilon$ vanishes uniformly as $\epsilon$ goes to 0 and (3) formally degenerates towards the pressureless gas system [3, 4]. On the other hand, in the congested region, $\rho$ is very close to 1, and the singularity $(1-\rho )^{-(\gamma +1)}$ compensates the degeneracy in $\epsilon$. The limit $\epsilon \rightarrow 0$ in model (3)-(2) (see [10]) is then viewed as a transition towards a granular suspension model, where the interacting force is governed by the contact between the solid grains. The Aw-Rascle system with this choice of offset function was investigated by the authors of [10] on a one-dimensional periodic domain, where the global well-posedness for fixed $\epsilon$ was studied, as well as the limit $\epsilon \rightarrow 0$, which is known as the ‘hard-congestion limit’. The authors demonstrated that up to a subsequence, solutions of (1)-(2) converge towards weak solutions of the ‘hard-congestion model’ (see also [6, 19]): 
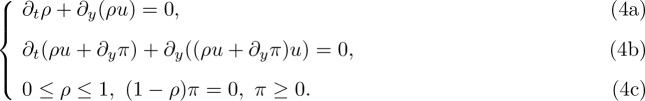
 In this sense, the original system (1)-(2) may be referred to as an approximation of (4). System (4) is an example of a free-congested system, where the free phase refers to the region where $\rho < 1$ and the congested region is where $\rho = 1$. The potential $\pi$ is obtained in the limit and can be viewed as a Lagrange multiplier associated with the incompressibility constraint $\partial _{x}u=0$ which holds in the congested region. The existence of strong solutions to the system (4) is still not known and there are also no results on the stability of non-trivial solutions (i.e. where $\pi$ is not identically 0). Note however that the existence of weak and measure-valued (duality) solutions to this model was recently obtained on the real line in [9]. We also refer to [8, 14, 17] and the references therein for further examples of the theoretical and numerical analysis pertaining to the Aw-Rascle system.

In the present work, we are concerned with the stability of a specific class of solutions to the system (1)-(2), namely the solutions $(\rho , u)$ which are travelling waves that connect a congested left state ($\rho = 1$) to a non-congested right state ($\rho < 1$). Since systems (1)-(2) and (3) are equivalent (for sufficiently regular solutions), our task is closely related to the stability of travelling wave solutions to the compressible Navier-Stokes system, which has been studied by Dalibard and Perrin in [12]. There, the authors studied system (3) with the addition of a pressure and a constant viscosity coefficient $\mu >0$. A similar study was also carried out in [23] by Vasseur and Yao. Both of these works make use of a new formulation of the system, which is obtained by introducing the ‘effective velocity’ *w* and rewriting the system in terms of (*w*, *v*), where $v:=1/\rho$ is the specific volume. The parabolic equation satisfied by *v* and the transport structure for *w* then allows for desirable energy estimates, which are carried out with the help of integrated variables (see Equation (12) below). Rewriting the system using the effective velocity has also proven to be advantageous when investigating the existence and uniqueness of weak/strong solutions to the compressible Navier-Stokes system with a density-dependent viscosity of the form $\mu (\rho ) = \rho ^{\alpha }$ for $\alpha > 0$ (see [7, 11] for example).

To the best of our knowledge, there are no known results concerning the stability of shock waves where the viscosity coefficient is singular. The interest in such a result is twofold. Firstly, the stability of strong solutions to (1)-(2) is significant due to the equivalence with the compressible Navier-Stokes system (3), for which such a result does not exist in the literature as far as we know. On the other hand, a stability result for partially congested solutions would imply that the system (4) is also expected to be stable, which provides additional validity for the model (4) and further verifies the need for a rigid well-posedness theory. Note that in our case the presence of a singular, degenerate viscosity coefficient and the lack of pressure prevents us from using the arguments of [12, 23] to obtain the estimates required to prove the global existence and stability of travelling wave solutions to (1)-(2). Nonetheless, we demonstrate in this paper that through a careful choice of weighted energy estimates and the identification of good unknowns taking congestion into account, we can obtain results analogous to those of [12] for the Aw-Rascle system (1) with the singular offset function (2).

Let us now give an outline of the paper. In the next subsection we detail our main results, which are the existence of travelling wave solutions to system (1)-(2), and the nonlinear stability of these solutions. Then in Section 2 we introduce basic properties of travelling wave solutions, an integrated formulation of the system and some useful preliminary estimates. The bulk of our work goes into Section 3, where we obtain the well-posedness of the integrated system. Lastly, we prove the equivalence between the integrated system and the original system in Section 4.

### Main Results

We now mention our main results. Let the Lagrangian mass coordinate *x* be such that $\textrm{d}x=\rho \textrm{d}y-\rho u\textrm{d}t$, and $v:=1/\rho$ the specific volume. Then (3) becomes

5 $$\begin{aligned} \left\{ \begin{aligned}&\partial _t v -\partial _x u=0,\\&\partial _t u -\partial _x(\phi _\epsilon (v)\partial _x u)=0, \end{aligned} \right. \end{aligned}$$

where

6 $$\begin{aligned} \phi _\epsilon (v) := \frac{p_\epsilon '(1/v)}{v^3} =\frac{\epsilon \gamma }{v(v-1)^{\gamma +1}}, \qquad \textrm{such}\,\,\,\textrm{that}\qquad \partial _y p_\epsilon (\rho ) = -\phi _\epsilon (v)\partial _x v. \end{aligned}$$

In these coordinates, it follows that

$$\begin{aligned} w = u +\partial _y p_\epsilon (\rho ) = u - \phi _\epsilon (v) \partial _x v \end{aligned}$$

is constant, i.e. solves $\partial _t w =0$.

Our first lemma establishes the existence of travelling wave solutions, and gives a quantitative description of the profile.

#### Proposition 1.1

Let $$1=v_- < v_{+}$$ and $$ u_{-} > u_{+}$$ be real numbers. Then there exists a unique (up to a translation) travelling wave solution $$(\textbf{u},\textbf{v})(t,x) = (u_{\epsilon }, v_{\epsilon })(\xi )$$ of (5), complemented with the far field condition $$(\textbf{u},\textbf{v})\rightarrow (u_\pm ,v_\pm )$$ as $$\xi \rightarrow \pm \infty $$, where $$\xi := x-st$$ and *s* is the shock speed which satisfies

7 $$\begin{aligned} s = \frac{u_{-}-u_{+}}{v_{+}-1}. \end{aligned}$$

The solution is a smooth monotone increasing function connecting 1 (at $-\infty$) to $v_+$ (at $+\infty$).

By setting $v_\epsilon (0) =(1+v_+)/2$, one then has the following estimates:

- In the congested region $(\xi <0)$,
  8 $$\begin{aligned} 1 + \left( B-\frac{A_0}{\epsilon }\xi \right) ^{-1/\gamma } \le v_\epsilon (\xi ) \le 1+\left( B-\frac{A_1}{\epsilon }\xi \right) ^{-1/\gamma }, \end{aligned}$$
  where
  $$\begin{aligned} A_0:= \frac{s(v_+-1)(v_+ +1)}{2}, \qquad A_1:= \frac{s(v_+-1)}{2}, \qquad \textrm{and} \qquad B:= \left( \frac{2}{v_+-1}\right) ^\gamma . \end{aligned}$$
- In the free region $\xi >0$,
  9 $$\begin{aligned} v_+-\frac{v_+-1}{2}\exp \left( -\frac{A_2}{\epsilon }\xi \right) \le v_\epsilon (\xi ) \le v_+-\frac{v_+-1}{2}\exp \left( -\frac{A_3}{\epsilon }\xi \right) , \end{aligned}$$
  where
  $$\begin{aligned} A_2:= \frac{s(v_++1)(v_+-1)^{\gamma +1}}{2^{\gamma +2}\gamma }\qquad \textrm{and} \qquad A_3 := \frac{s v_+(v_+-1)^{\gamma +1}}{\gamma }. \end{aligned}$$

It follows that $v_\epsilon$ converges almost everywhere to the shock wave $v(\xi ):= \textbf{1}_{\xi <0}+v_+\textbf{1}_{\xi >0}$ as $\epsilon \rightarrow 0$, and it holds that

10 $$\begin{aligned} v_\epsilon (\xi )\le \left[ 1+\left( B-\frac{A_1}{\epsilon }\xi \right) ^{-1/\gamma }\right] \textbf{1}_{\xi <0}+v_+\textbf{1}_{\xi \ge 0}. \end{aligned}$$

With this in hand, we dedicate the rest of our effort towards studying the stability of the profiles $(u_{\epsilon }, v_{\epsilon })$ where $\epsilon< < 1$. We first express System (5) in term of the unknowns *v* and $w=u-\phi _\epsilon (v)\partial _x v$:

11 $$\begin{aligned} \left\{ \begin{aligned}&\partial _t w = 0,\\&\partial _t v -\partial _x w - \partial _x (\phi _\epsilon (v)\partial _x v)=0. \end{aligned} \right. \end{aligned}$$

In order to obtain effective energy estimates, we take inspiration from [12, 23] and choose to re-write this system in terms of the integrated variables. Suppose we have initial data $(w_{0}, v_{0})$ such that $(w_{0} - (w_{\epsilon })(0), v_{0} - (v_{\epsilon })(0)) \in (L^{1}_{0}(\mathbb {R}) \cap L^{\infty }(\mathbb {R}))^{2}$ where $L^{1}_{0}(\mathbb {R})$ is the subset of $L^{1}(\mathbb {R})$ consisting of zero mean functions. Then we define the integrated initial data as

$$\begin{aligned} W_{0}(x) := \int _{-\infty }^{x} (w_{0}(z) - w_{\epsilon }(0,z))~dz, \quad V_{0}(x) := \int _{-\infty }^{x} (v_{0}(z) - v_{\epsilon }(0,z))~dz. \end{aligned}$$

Supposing that $(w-w_{\epsilon }, v- v_{\epsilon })(t) \in L^{1}_{0}(\mathbb {R})$ holds true for positive times, we may also define the integrated variables

12 $$\begin{aligned} W(t,x) := \int _{-\infty }^{x} (w(t,z) - w_{\epsilon }(t,z))~dz, \quad V(t,x) := \int _{-\infty }^{x} (v(t,z) - v_{\epsilon }(t,z))~dz. \end{aligned}$$

Integrating (11) between $-\infty$ and *x* formally, we see that (*W*, *V*) solves

13 $$\begin{aligned} \left\{ \begin{aligned}&\partial _t W = 0,\\&\partial _t V -\partial _x W - \phi _\epsilon (v)\partial _x v+\phi _\epsilon (v_\epsilon )\partial _xv_\epsilon =0, \\&(W,V)(0, \cdot ) = (W_{0}, V_{0}). \end{aligned} \right. \end{aligned}$$

Since *W* is constant in time, we will denote it by its initial value $W_0$ from now on. The following result pertains to this system.

#### Theorem 1.2

(Existence of a strong solution to the integrated system) There exists a constant $\delta _0>0$, depending only on $s, v_+$ and $\gamma \ge 1$, such that the following result holds. Let $T>0$ and the initial condition $(V_0,W_0)$ be such that:

- $$V_0, W_0 \in H^2(\mathbb {R})$$;
- $$\eta _0:=\dfrac{\partial _x V_0}{v_{\epsilon }|_{t=0}-1} \in H^1(\mathbb {R})$$;
- $$\sqrt{x}\partial _x^kW_0\in L^2(\mathbb {R}_+)$$, for $$k=0,1,2$$.

If the following estimate holds:

14 $$\begin{aligned} \begin{aligned} \Vert (V_0,W_0,\epsilon \eta _0,\epsilon ^2\partial _x\eta _0)\Vert ^2_{L^2(\mathbb {R})}+ \sum _{k=0}^2\epsilon ^{2k-1}\Vert \sqrt{x}\partial _x^kW_0\Vert ^2_{L^2(\mathbb {R}_+)}&\\ + \left( \frac{T}{\epsilon }\right) ^{1/\gamma }\left( \epsilon ^2\Vert \partial _x W_0\Vert ^2_{L^2(\mathbb {R})}+\epsilon ^4\Vert \partial _x^2W_0\Vert ^2_{L^2(\mathbb {R})}\right)&\le \delta _0\epsilon ^3, \end{aligned} \end{aligned}$$

then there exists a unique solution $(W_0,V)$ to (13) on (0, *T*) such that

$$\begin{aligned}&V \in C([0,T]; H^{2}(\mathbb {R})) \cap L^{2}(0,T; H^{3}(\mathbb {R})). \end{aligned}$$

Moreover, there exists a constant $C = C(s,v_{+}, \gamma , \delta _{0})$ such that

15 $$\begin{aligned} \begin{aligned} \sup _{t \in [0,T]}&\left( \int _{\mathbb {R}} V^{2}~dx + \int _{0}^{t} \int _{\mathbb {R}} \phi _{\epsilon }(v_{\epsilon })|\partial _{x}V|^{2}~dxds \right) \\  &+ \sup _{t \in [0,T]} \left( \sum _{k=0}^{1}\epsilon ^{2k+2} \left[ \int _{\mathbb {R}} \left| \partial _{x}^{k}\left( \frac{\partial _{x}V}{v_{\epsilon }-1} \right) \right| ^{2} ~dx + \int _{0}^{t} \int _{\mathbb {R}} \phi _{\epsilon }(v_{\epsilon })\left| \partial _{x}^{k+1}\left( \frac{\partial _{x}V}{v_{\epsilon }-1} \right) \right| ^{2} ~dxds \right] \right) \le C \epsilon ^{3}. \end{aligned} \end{aligned}$$

#### Remark 1.3

It follows from Proposition 1.1 that the coefficient of diffusion $\phi _\epsilon (v_\epsilon )$ (see (6) above) is singular and tends to $+\infty$ as *x* tends to $-\infty$. As a consequence, we cannot close the estimates on *V* in the classical Sobolev spaces $H^s$. This is why we work with the weighted quantity $\eta :=\partial _x V/(v_\epsilon -1)$ instead (see (34)), which yields better estimates. The drawback of this method is that the time-independent quantity $W_0$ cannot be bounded uniformly in time in such weighted spaces. Hence we only obtain local in time well-posedness when $W_0\ne 0$, with a time of existence *T* proportional to $1/\Vert \partial _x W_0\Vert _{H^1}^{2\gamma }$.

#### Remark 1.4

The assumption $\sqrt{x} \partial _{x}^{k}W_0 \in L^{2}(\mathbb {R}_{+})$ for $k=0,1,2$ is classical for this kind of system and was already used in [13]. However, it is possible to remove this assumption and only assume that $W_0\in H^2(\mathbb {R})$. One then obtains a shorter time of existence, proportional to $1/\Vert W_0\Vert _{H^2}^2$ (see Remark 3.12 below).

#### Remark 1.5

Using $v_{\epsilon } < v_{+}$ and the regularity of $v_\epsilon$, the bound (15) implies that $V \in L^{\infty }(0,T; H^{2}(\mathbb {R})) \cap L^{2}(0,T; H^{3}(\mathbb {R}))$. Furthermore, by using the smallness assumption (14), we will show the lower bound $v>1$ for every *t*, *x*. In other words, the perturbation does not reach the congested state (see Remark 3.1).

#### Remark 1.6

Note that Theorem 1.2 provides a local-in-time result when $W_0\ne 0$. A natural question which follows is whether it is possible to obtain such a result without using the integrated variables formulation. An answer to this question is given in Remark 3.13, where we show that one can obtain a result on a finite time interval without the assumption of integrable perturbations. However, the time of existence of solutions obtained using this method is much shorter than the one given by Theorem 1.2, as it depends on the norm of both the initial perturbation in *v* and *w* (whereas the existence time resulting from Theorem 1.2 only depends on the perturbation in *w*). More details are given in Remark 3.13.

Under the same assumptions, we also prove a stability result.

#### Theorem 1.7

(Nonlinear stability of travelling wave solutions) Let $T>0$, $\gamma \ge 1$. Assume that the initial data $(u_{0}, v_{0})$ is such that

16 $$\begin{aligned} u_{0} - (u_{\epsilon })_{t=0} \in W^{1,1}_{0}(\mathbb {R}) \cap H^{1}(\mathbb {R}), \quad {\frac{\partial _x[u_0-(u_\epsilon )_{t=0}]}{v_\epsilon -1}\in L^2(\mathbb {R})}, \quad v_{0} - (v_{\epsilon })_{t=0} \in W^{2,1}_{0}(\mathbb {R}) \cap H^{2}(\mathbb {R}), \end{aligned}$$

and the associated integrated initial data $(W_{0}, V_{0})$ satisfies (14). Then there exists a unique global solution (*u*, *v*) to (1) on [0, *T*] which satisfies

$$\begin{aligned}&u-u_{\epsilon } \in C([0,T]; H^{1}(\mathbb {R}) \cap L^{1}_{0}(\mathbb {R})), \\&v-v_{\epsilon } \in C([0,T]; H^{1}(\mathbb {R}) \cap L^{1}_{0}(\mathbb {R})) \cap L^{2}(0,T; H^{2}(\mathbb {R})). \end{aligned}$$

In particular, there exists a constant $C_{1}>0$ dependent on $\gamma , v_{+}, T$ such that

$$\begin{aligned} \Vert u-u_{\epsilon }\Vert _{L^{\infty }(0,T; H^{1}(\mathbb {R}))} + \Vert v-v_{\epsilon }\Vert _{L^{\infty }(0,T; H^{1}(\mathbb {R}))} + \Vert v-v_{\epsilon }\Vert _{L^{2}(0,T; H^{2}(\mathbb {R}))} \le C_{1}. \end{aligned}$$

There also exists a constant $C_{2} > 0$ dependent on $\gamma , v_{+}, T, \epsilon$ such that

$$\begin{aligned} \Vert u-u_{\epsilon }\Vert _{L^{\infty }(0,T; L^{1}(\mathbb {R}))} + \Vert v-v_{\epsilon }\Vert _{L^{\infty }(0,T; L^{1}(\mathbb {R}))} \le C_{2}. \end{aligned}$$

As a consequence of the estimates derived during the course of proving the above theorem, we are also able to assert global existence with $T = + \infty$ and long-time stability if we remove the perturbation on the desired velocity.

#### Corollary 1.8

If we additionally assume that

17 $$\begin{aligned} (u - u_{\epsilon })(0) = (\partial _{x}\phi _{\epsilon }(v) - \partial _{x}\phi _{\epsilon }(v_{\epsilon }))(0), \end{aligned}$$

i.e. that $(w - w_{\epsilon })(0) = 0$, then the solution (*u*, *v*) is defined on $\mathbb {R}_{+} \times \mathbb {R}$, satisfies (15) with $T=+\infty$ and additionally

$$\begin{aligned} \sup _{x \in \mathbb {R}} \left( |v-v_{\epsilon }|(t,x) + |u-u_{\epsilon }|(t,x) \right) \rightarrow 0 \text { as } t \rightarrow \infty . \end{aligned}$$

#### Remark 1.9

All of our results remain true if we replace (2) by

$$\begin{aligned} w = u+ \partial _y \tilde{p}_\epsilon (\rho ) = u +\partial _y\left( \frac{\epsilon f(\rho )}{(1-\rho )^{\gamma }}\right) ,\qquad \gamma \ge 1, \end{aligned}$$

where *f* is smooth on $\mathbb {R}_+^*$ and such that $\gamma f(\rho )+(1-\rho )f'(\rho )>0$, i.e. such that $\tilde{p}_\epsilon '(\rho )>0$. In this case, one obtains that

18 $$\begin{aligned} \phi _\epsilon (v) = \epsilon v^{\gamma -3}\frac{(v-1)f'(1/v) + \gamma f(1/v)}{(v-1)^{\gamma +1}}. \end{aligned}$$

In order to improve readability and without loss of generality, we will stick to the case $f(\rho ) = \rho ^{\gamma }$, for which the coefficient $\phi _\epsilon$ can be written in the more compact form (6). This is also the form considered in [10]. All computations are similar (but heavier) in the general case (18).

## Properties of the Travelling Wave Solutions

### Existence and Asymptotic Behavior of the Travelling Waves

We give here the proof of Proposition 1.1.

#### Proof

We first prove the existence of travelling wave solutions to (5):

$$\begin{aligned} \left\{ \begin{aligned}&\partial _t v -\partial _x u=0,\\&\partial _t u -\partial _x(\phi _\epsilon (v)\partial _x u)=0, \end{aligned} \right. \end{aligned}$$

We look for a travelling wave solution of this system, i.e. a pair $(u_\epsilon ,v_\epsilon )$ where $u_\epsilon ,v_\epsilon$ are functions of the variable $\xi =x-st$, *s* being the speed of propagation of the solution. We also suppose that $(u_\epsilon ,v_\epsilon )\rightarrow (u_\pm ,v_\pm )$ and that $(u_\epsilon ',v_\epsilon ')\rightarrow (0,0)$ as $\xi$ goes to $\pm \infty$. We first obtain

$$\begin{aligned} \left\{ \begin{aligned}&-sv_\epsilon '-u_\epsilon '=0,\\&-s u_\epsilon '-(\phi _\epsilon (v_\epsilon )u_\epsilon ')'=0. \end{aligned} \right. \end{aligned}$$

Integrating this equation between $\xi$ and $+\infty$, we get

$$\begin{aligned} \left\{ \begin{aligned}&sv_\epsilon +u_\epsilon =sv_++u_+,\\&s u_\epsilon +\phi _\epsilon (v_\epsilon )u_\epsilon '=su_+. \end{aligned} \right. \end{aligned}$$

The first equation yields $u_\epsilon = sv_++u_+-sv_\epsilon$. Substituting this into the second line, we deduce that $v_\epsilon$ solves the following ODE:

19 $$\begin{aligned} v_\epsilon ' =\frac{s(v_+-v_\epsilon )}{\phi _\epsilon (v_\epsilon )}=\frac{s(v_+-v_\epsilon )v_\epsilon (v_\epsilon -1)^{\gamma +1}}{\epsilon \gamma }. \end{aligned}$$

**Fig. 1:**
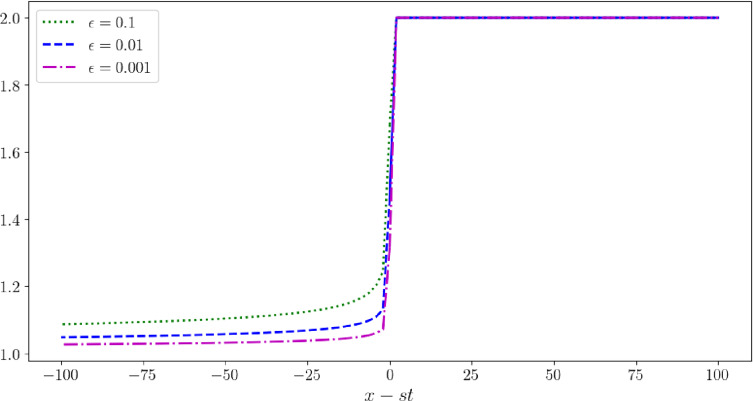
The profile of $v_{\epsilon }$, where we fix $v_{\epsilon }(0) = (1 + v_{+})/2$ and $v_{+} = 2, \gamma = 5$

Assuming $v_+>1$ and $s>0$, we may deduce that $v_-=1$, and $u_-=s(v_+-1)+u_+$, or equivalently $s=(u_--u_+)/(v_+-1)$. The function $v_\epsilon$ is therefore an increasing function taking values in the interval $(1, v_{+})$ (see Figure 1). The Cauchy-Lipschitz theorem thus yields that $v_\epsilon$ is the unique (up to a translation) global solution of (19), as stated in Proposition 1.1. Note that, as $v_\epsilon$ approaches 1, the diffusion coefficient $\phi _\epsilon (v_\epsilon )$ tends to $+\infty$.

Let us now make this statement more quantitative. From now on, suppose that $v_\epsilon (0)= (1+v_+)/2$, i.e. $v_\epsilon (0)$ is halfway between 1 and $v_+$. Let $\xi <0$. From the ODE (19) and the monotonicity of $v_\epsilon$, we obtain the following bound:

$$\begin{aligned} \frac{s(v_+-1)(v_\epsilon (\xi )-1)^{\gamma +1}}{2\epsilon \gamma }\le v_\epsilon '(\xi ) \le \frac{s(v_+-1)(v_+ +1)(v_\epsilon (\xi )-1)^{\gamma +1}}{2\epsilon \gamma }. \end{aligned}$$

Dividing by $(v_\epsilon (\xi )-1)^{\gamma +1}$ and integrating between $\xi$ and 0 yields

$$\begin{aligned} - \frac{s(v_+-1)}{2\epsilon \gamma }\xi \le -\frac{2^\gamma }{\gamma (v_+-1)^\gamma } + \frac{1}{\gamma (v_\epsilon (\xi )-1)^\gamma } \le -\frac{s(v_+-1)(v_+ +1)}{2\epsilon \gamma }\xi , \end{aligned}$$

i.e.

$$\begin{aligned} 1 + \left( B-\frac{A_0}{\epsilon }\xi \right) ^{-1/\gamma } \le v_\epsilon (\xi ) \le 1+\left( B-\frac{A_1}{\epsilon }\xi \right) ^{-1/\gamma }, \end{aligned}$$

where

$$\begin{aligned} A_0:= \frac{s(v_+-1)(v_+ +1)}{2}, \qquad A_1:= \frac{s(v_+-1)}{2}, \,\,\, \textrm{and} \,\,\, B:= \left( \frac{2}{v_+-1}\right) ^\gamma . \end{aligned}$$

Estimate (8) follows. When $\xi >0$, one has similarly

$$\begin{aligned} \frac{s(v_++1)(v_+-1)^{\gamma +1}}{2^{\gamma +2}\epsilon \gamma } \le \frac{v_\epsilon '(\xi )}{v_+-v_\epsilon (\xi )} \le \frac{sv_+(v_+-1)^{\gamma +1}}{\epsilon \gamma }, \end{aligned}$$

i.e.

$$\begin{aligned} \frac{A_2}{\epsilon } \le \frac{v_\epsilon '(\xi )}{v_+-v_\epsilon (\xi )} \le \frac{A_3}{\epsilon }, \end{aligned}$$

with

$$\begin{aligned} A_2:= \frac{s(v_++1)(v_+-1)^{\gamma +1}}{2^{\gamma +2}\gamma } \quad \textrm{and} \quad A_3:= \frac{sv_+(v_+-1)^{\gamma +1}}{\gamma } . \end{aligned}$$

Integrating between 0 and $\xi$ yields

$$\begin{aligned} \frac{A_2}{\epsilon }\xi \le -\ln [v_+-v_\epsilon (\xi )] + \ln \left[ \frac{v_+-1}{2}\right] \le \frac{A_3}{\epsilon }\xi , \end{aligned}$$

i.e.

$$\begin{aligned} \frac{v_+-1}{2}\exp \left( -\frac{A_2}{\epsilon }\xi \right) \ge v_+-v_\epsilon (\xi ) \ge \frac{v_+-1}{2}\exp \left( -\frac{A_3}{\epsilon }\xi \right) , \end{aligned}$$

which is the desired estimate. $\square$

### Passage to the Integrated System and Reformulation of the Equations

We now want to study the stability of the travelling wave solution obtained in the previous section. In order to do so, we first rewrite (5) in term of the unknowns *v* and $w =u-\phi _\epsilon (v)\partial _x v$:

20 $$\begin{aligned} \left\{ \begin{aligned}&\partial _t w = 0,\\&\partial _t v -\partial _x w - \partial _x (\phi _\epsilon (v)\partial _x v)=0. \end{aligned} \right. \end{aligned}$$

Let $(w_\epsilon ,v_\epsilon )$ denote the travelling wave solution. It solves the same system:

21 $$\begin{aligned} \left\{ \begin{aligned}&\partial _t w_\epsilon = 0,\\&\partial _t v_\epsilon -\partial _x w_\epsilon - \partial _x (\phi _\epsilon (v_\epsilon )\partial _x v_\epsilon )=0. \end{aligned} \right. \end{aligned}$$

Taking the difference between these two systems yields

22 $$\begin{aligned} \left\{ \begin{aligned}&\partial _t(w- w_\epsilon )= 0,\\&\partial _t (v-v_\epsilon ) -\partial _x (w-w_\epsilon ) - \partial _x(\phi _\epsilon (v)\partial _x v)+\partial _x(\phi _\epsilon (v_\epsilon )\partial _x v_\epsilon )=0. \end{aligned} \right. \end{aligned}$$

Following [12, 23], we introduce *W*, *V* such that

23 $$\begin{aligned} W(t,x):=\int _{-\infty }^x(w(t,x')-w_\epsilon (t,x'))\textrm{d}x'\,\,\,\textrm{and}\,\,\, V(t,x):=\int _{-\infty }^x(v(t,x')-v_\epsilon (t,x'))\textrm{d}x'. \end{aligned}$$

Integrating (22) between $-\infty$ and *x* yields for (*W*, *V*) the following system:

$$\begin{aligned} \left\{ \begin{aligned}&\partial _tW = 0,\\&\partial _t V -\partial _x W - \phi _\epsilon (v)\partial _x v+\phi _\epsilon (v_\epsilon )\partial _xv_\epsilon =0. \end{aligned} \right. \end{aligned}$$

Introducing $\psi _\epsilon$ such that $\psi _\epsilon '=\phi _\epsilon$, we obtain that $W=W_0$ is constant and

24 $$\begin{aligned} \partial _t V -\partial _x W_0 -\partial _x(\psi _\epsilon (v)-\psi _\epsilon (v_\epsilon ))=0. \end{aligned}$$

We first notice that we can replace *v* by $v_\epsilon +\partial _xV$. In order to write the system as a linearized part around $v_\epsilon$ plus a perturbation part, we also move the terms involving $\psi _\epsilon$ to the right-hand side and subtract $\partial _x(\phi _\epsilon (v_\epsilon )\partial _xV)$ from both sides:

$$\begin{aligned} \partial _t V -\partial _x W_0 -\partial _x(\phi _\epsilon (v_\epsilon )\partial _xV)=\partial _x\left[ \psi _\epsilon (v_\epsilon +\partial _xV)-\psi _\epsilon (v_\epsilon )-\psi _\epsilon '(v_\epsilon )\partial _x V\right] . \end{aligned}$$

This previous equation can be written in the following compact way:

25 $$\begin{aligned} \partial _t V -\partial _x W_0 -\partial _x(\phi _\epsilon (v_\epsilon )\partial _xV)=\partial _x H(\partial _xV), \end{aligned}$$

with

26 $$\begin{aligned} H(f):=\psi _\epsilon (v_\epsilon +f)-\psi _\epsilon (v_\epsilon )-\psi _\epsilon '(v_\epsilon )f. \end{aligned}$$

### Preliminary Estimates

We give in this section some estimates on the functions $v_\epsilon$, $\psi _\epsilon$, *H* and their derivatives.

#### Lemma 2.1

(Estimates on $v_\epsilon$) For any $k\ge 1$, there exists a constant $C= C(k,\gamma ,v_+,s)$ such that

27 $$\begin{aligned} \forall \epsilon >0, \qquad \left| \partial _x^kv_\epsilon \right| \le \frac{C(v_\epsilon -1)^{k\gamma +1}}{\epsilon ^k}. \end{aligned}$$

#### Proof

By using the ODE (19) satisfied by $v_\epsilon$, one can show by induction that for every $k\ge 1$, there exists a function $f_k$ independent of $\epsilon$ such that

- $f_k$ is smooth on $\mathbb {R}_+^*$,
- $$f_k(1)\ne 0$$,
- $$\partial _x^kv_\epsilon =f_k(v_\epsilon )(v_\epsilon -1)^{k\gamma +1}/\epsilon ^k$$.

Since $f_k$ is continuous on $[1,v_+]$ and $1<v_\epsilon <v_+$, the factor $f_k(v_\epsilon )$ is uniformly bounded. $\square$

#### Lemma 2.2

(Estimates on $\psi _\epsilon$) Let $\bar{v}>1$ be arbitrary. For any $k\ge 1$, there exists $C= C(k,\gamma ,\bar{v})$ such that, for every $v\in (1,\bar{v})$, for every $\epsilon >0$,

28 $$\begin{aligned} \left| \psi _\epsilon ^{(k)}(v)\right| \le \frac{C\epsilon }{(v-1)^{\gamma +k}}. \end{aligned}$$

#### Proof

As in the previous lemma, one can show that there exists a smooth function $g_k$ defined on $\mathbb {R}_+^*$, independent of $\epsilon$, such that $\psi _\epsilon ^{(k)}(v)=\epsilon g_k(v)(v-1)^{-\gamma -k}$ and $g_k(1)\ne 0$.

Since $g_k$ is smooth, we can bound it on $[1, \bar{v}]$. $\square$

As a consequence of Lemmas 2.1 and 2.2, we obtain the following estimates:

#### Lemma 2.3

The functions $\phi _\epsilon ^{1/2}\partial _x v_\epsilon$ and $\phi _\epsilon ^{1/2}\dfrac{\partial _x^kv_\epsilon }{v_\epsilon -1}$, $k\ge 2$, belong to $L^2(\mathbb {R})$, with a time-independent $L^2(\mathbb {R})$ norm.

#### Proof

We first see with Proposition 1.1 and the ODE (19) satisfied by $v_\epsilon$ that, for any $k\ge 1$, $\partial _x^kv_\epsilon \in L^1(\mathbb {R})\cap L^\infty (\mathbb {R})$. Since $\phi _\epsilon$ and $v_\epsilon -1$ go to a positive and finite limit as $\xi$ goes to $+\infty$, the integrability of the functions $\phi _\epsilon ^{1/2}\partial _x v_\epsilon$ and $\phi _\epsilon ^{1/2}\dfrac{\partial _x^kv_\epsilon }{v_\epsilon -1}$ at $+\infty$ is a consequence of the one of $\partial _x^k v_\epsilon$.

Concerning the integrability when $\xi \rightarrow -\infty$, we first see with Lemma 2.1 that there exists $C=C(\epsilon )>0$ such that

$$\begin{aligned} |\phi _\epsilon ^{1/2}\partial _x v_\epsilon | \le C(v_\epsilon -1)^{(\gamma +1)/2},\quad \textrm{and} \quad \left| \phi _\epsilon ^{1/2}\frac{\partial _x^kv_\epsilon }{v_\epsilon -1}\right| \le C(v_\epsilon -1)^{(2k-1)\gamma /2-1/2}. \end{aligned}$$

By Proposition 1.1, we deduce that

$$\begin{aligned} |\phi _\epsilon ^{1/2}\partial _x v_\epsilon | \le C\left( B-\frac{A_1}{\epsilon }\xi \right) ^{-1/2-1/(2\gamma )}\quad \textrm{and}\quad \left| \phi _\epsilon ^{1/2}\frac{\partial _x^kv_\epsilon }{v_\epsilon -1}\right| \le C\left( B-\frac{A_1}{\epsilon }\xi \right) ^{1/2+1/(2\gamma )-k}\in L^2(\mathbb {R}_-) \end{aligned}$$

since $k\ge 2$. Note that we consider functions that depend only on the variable $\xi =x-st$, and so the $L^2$-norms are independent of time. $\square$

#### Lemma 2.4

(Bounds on H) Let *f* such that there exists $\delta <1$ with $\Vert \frac{f}{v_\epsilon -1}\Vert _\infty \le \delta$. Then one has the following bounds on *H*(*f*):

29 $$\begin{aligned}  &   |H(f)|\le \frac{C\epsilon }{(v_\epsilon -1)^{\gamma +2}}f^2, \end{aligned}$$

30 $$\begin{aligned}  &   |\partial _xH(f)|\le \frac{C}{(v_\epsilon -1)^2}f^2+\frac{C\epsilon }{(v_\epsilon -1)^{\gamma +2}}|f||\partial _x f|, \end{aligned}$$

31 $$\begin{aligned}  &   |\partial _{x}^2H(f)|\le \frac{C(v_\epsilon -1)^{\gamma -2}}{\epsilon }f^2+\frac{C\epsilon }{(v_\epsilon -1)^{\gamma +2}}|f|| \partial _{x}^2f| + \frac{C\epsilon }{(v_\epsilon -1)^{\gamma +2}}|\partial _xf|^2, \end{aligned}$$

for some $$C= C(s,\gamma ,\delta , v_+)$$.

#### Proof

We first prove (29). Recall that

$$\begin{aligned} H(f)=\psi _\epsilon (v_\epsilon +f)-\psi _\epsilon (v_\epsilon )-\psi _\epsilon '(v_\epsilon )f. \end{aligned}$$

By Taylor’s theorem,

$$\begin{aligned} |H(f)|\le \frac{f^2}{2}\sup _{|v-v_\epsilon |\le |f|}|\psi _\epsilon ^{(2)}(v)|. \end{aligned}$$

By the hypothesis on *f*, for any *v* such that $|v-v_\epsilon |\le |f|$, it holds $0<(1-\delta )(v_\epsilon -1)<v-1<2v^+$.

Lemma 2.2 then implies that $|\psi _\epsilon ^{(2)}(v)|\le C\epsilon (v-1)^{-(\gamma +2)}$. Hence

$$\begin{aligned} |H(f)|\le \frac{C\epsilon }{(v_\epsilon -1)^{\gamma +2}}f^2, \end{aligned}$$

for some constant *C* depending on $\gamma , v_+,\delta$. This is the first inequality. The other inequalities are proved in the same way. We differentiate *H* with respect to *x* and obtain

$$\begin{aligned} \partial _xH(f)=\partial _xv_\epsilon \left( \psi _\epsilon '(v_\epsilon +f)-\psi _\epsilon '(v_\epsilon )-\psi _\epsilon ^{(2)}(v_\epsilon )f\right) +\partial _xf\left( \psi _\epsilon '(v_\epsilon +f)-\psi _\epsilon '(v_\epsilon )\right) . \end{aligned}$$

which yields (30) by the same arguments and Lemmas 2.1, 2.2.

We now differentiate a second time with respect to *x*:

$$\begin{aligned} \partial _{xx}H(f)=&\partial _{xx}v_\epsilon \left( \psi _\epsilon '(v_\epsilon +f)-\psi _\epsilon '(v_\epsilon )-\psi _\epsilon ^{(2)}(v_\epsilon )f\right) + (\partial _xf)^2\psi _\epsilon ^{(2)}(v_\epsilon +f)\\&+ (\partial _x v_\epsilon )^2\left( \psi _\epsilon ^{(2)}(v_\epsilon +f)-\psi _\epsilon ^{(2)}(v_\epsilon )-\psi _\epsilon ^{(3)}(v_\epsilon )f\right) \\&+2\partial _xf\partial _x v_\epsilon \left( \psi _\epsilon ^{(2)}(v_\epsilon +f)-\psi _\epsilon ^{(2)}(v_\epsilon )\right) +\partial _{xx}f\left( \psi _\epsilon '(v_\epsilon +f)-\psi _\epsilon '(v_\epsilon )\right) . \end{aligned}$$

which yields (31) by similar computations. $\square$

## Well-Posedness for the Integrated System

### Basic Energy Estimate

The goal of this section is to prove the existence of strong solutions to the equation

32 $$\begin{aligned} \partial _t V -\partial _x W_0 -\partial _x(\phi _\epsilon (v_\epsilon )\partial _xV)=\partial _x H(\partial _xV). \end{aligned}$$

Since $W_0$ is constant in time, the term $\partial _xW_0$ is moved to the right-hand side and treated as a source term. We are thus left to prove the existence of a strong solution *V* to (32). Our strategy is to employ a fixed-point argument, which requires us to first derive appropriate energy estimates. The first (zero-th order) estimate is obtained by multiplying (32) by *V*:

33 $$\begin{aligned} \begin{aligned} \frac{1}{2}\int _{\mathbb {R}} V^{2}(t)~dx + \int _{0}^{t} \int _{\mathbb {R}} \phi _{\epsilon }(v_{\epsilon }) |\partial _{x}V|^{2}~dxd{\tau } = \frac{1}{2}\int _{\mathbb {R}}V^{2}(0)~dx + \int _{0}^{t}\int _{\mathbb {R}} V (\partial _{x}H(\partial _x V) + \partial _{x}W_0)~dxd\tau . \end{aligned} \end{aligned}$$

From the left-hand side of the equation, we see that the natural energy space is $V\in L^\infty ([0,T],L^2(\mathbb {R}))$ and $\sqrt{\phi _\epsilon (v_\epsilon )}\partial _x V \in L^2((0,T)\times \mathbb {R})$. Let us now move to the right-hand side of (33) and try to close the estimate. For the term containing $W_0$, an integration by parts and Young’s inequality yield

$$\begin{aligned} \left| \int _0^t\int _{\mathbb {R}}V\partial _x W_0\right| = \left| -\int _0^t\int _{\mathbb {R}}W_0\partial _x V\right| \le \frac{1}{2}\int _0^t\int _{\mathbb {R}}\phi _\epsilon (v_\epsilon )|\partial _x V|^2+\frac{1}{2}\int _0^t\int _{\mathbb {R}}\frac{W_0^2}{\phi _\epsilon (v_\epsilon )}. \end{aligned}$$

Hence this term can be bounded through suitable assumptions on $W_0$. For the term containing $H(\partial _x V)$, we also perform an integration by parts and use the estimate (29) on *H* that we computed before:

$$\begin{aligned} \left| \int _0^t\int _{\mathbb {R}}V\partial _x H(\partial _x V)\right| = \left| -\int _0^t\int _{\mathbb {R}}H(\partial _x V)\partial _x V\right|&\le C\epsilon \int _0^t \int _{\mathbb {R}} \frac{|\partial _x V|^3}{(v_\epsilon -1)^{\gamma +2}}\\&\le C\epsilon \left\| \frac{1}{\phi _\epsilon (v_\epsilon )(v_\epsilon -1)^{\gamma +1}}\right\| _{L^\infty _{t,x}}\int _0^t\int _{\mathbb {R}}\phi _\epsilon (v_\epsilon )|\partial _x V|^2\frac{|\partial _x V|}{v_\epsilon -1} . \end{aligned}$$

By recalling that $\phi _\epsilon (v_\epsilon )=\dfrac{\epsilon \gamma }{v_\epsilon (v_\epsilon -1)^{\gamma +1}}$ (see (6)), we obtain that

$$\begin{aligned} \left| \int _0^t\int _{\mathbb {R}}V\partial _x H(\partial _x V)\right| \le C\int _0^t\int _{\mathbb {R}}\phi _\epsilon (v_\epsilon )|\partial _x V|^2\frac{|\partial _x V|}{v_\epsilon -1} \le C\left\| \frac{\partial _x V}{v_\epsilon -1}\right\| _{L^\infty _{t,x}}\int _0^t\int _{\mathbb {R}}\phi _\epsilon (v_\epsilon )|\partial _x V|^2, \end{aligned}$$

where $C=C(s,\gamma ,\delta )$. In order to close the estimate, we need that the norm of the quantity $\partial _x V/(v_\epsilon -1)$ in $L^\infty ([0,T]\times \mathbb {R})$ be small enough. The ideas of the proof of the well-posedness of (32) are thus the following:

- Derive a-priori energy estimates for the quantities $\partial _x V/(v_\epsilon -1)$ and $\partial _x[\partial _x V/(v_\epsilon -1)]$.
- Use the fact that $\partial _x V/(v_\epsilon -1) \in L^\infty ([0,T],H^1(\mathbb {R}))$ and the injection $H^1(\mathbb {R}) \hookrightarrow L^\infty (\mathbb {R})$ to complete the estimates and bound these quantities in the same function spaces as chosen for *V*.
- Use a fixed-point argument to obtain the local-in-time existence and uniqueness of strong solutions to (32).

#### Remark 3.1

Note that the assumption that $\Vert \partial _x V/(v_\epsilon -1)\Vert _{L^\infty _{t,x}}$ is small, which enables to close the previous estimate, is also the one needed to bound the quantity $H(\partial _xV)$ and its derivatives (see Lemma 2.4). This assumption has a simple interpretation; by recalling that $\partial _x V=v-v_\epsilon$, we see that it prevents the perturbation *v* from reaching 1. In other words, this assumption ensures that $\rho <1$, i.e. that the perturbation stays in the uncongested state. Recall however that $v\rightarrow 1$ as $x\rightarrow -\infty$. Therefore, although the perturbation does not enter the congested state, it becomes infinitely close to it.

#### Remark 3.2

As explained in the previous remark, it is crucial to bound the quantity $\partial _x V/(v_\epsilon -1)$. Note that adding the factor $1/(v_\epsilon -1)$ is not free, and the price to pay is that a lot of commutators appear and the equations become more complicated (see the next subsection). However, even if the weight $1/(v_\epsilon -1)$ is singular, the commutators are not difficult to bound. For instance,

$$\begin{aligned} \partial _x\left( \frac{\partial _x V}{v_\epsilon -1}\right) = \frac{\partial _x^2 V}{v_\epsilon -1}-\frac{v_\epsilon '}{(v_\epsilon -1)^2}\partial _x V. \end{aligned}$$

From the estimate on $v_\epsilon '$ that we derived in Lemma (2.1), it follows that the coefficient $v_\epsilon '/(v_\epsilon -1)^2$ is bounded by $C(v_\epsilon -1)^{\gamma -1}/\epsilon$. Hence this coefficient is uniformly bounded in *t*, *x* as soon as $\gamma \ge 1$, which is our hypothesis throughout the paper. The computations of the next subsections show that $\gamma \ge 1$ is the only hypothesis needed in order to obtain good bounds for every quantity which appears due to commutators. Furthermore, the factor $1/\epsilon$ in the coefficient $v_\epsilon '/(v_\epsilon -1)^2$ of the commutator suggests that the operator $\partial _x$ scales as $1/\epsilon$ for solutions of the equations, i.e. that $\Vert \partial _x f\Vert \cong \Vert f\Vert /\epsilon$. This observation is reflected in the definition of the norm to be seen in Sect. 3.3. Note that the scaling $\partial _x \sim 1/\epsilon$ is mostly useful for $x\rightarrow -\infty$. Indeed, the bound from Lemma 2.1 is not optimal when $x\rightarrow \infty$ and could be improved, but its current version is sufficient to obtain the results stated in this work.

### Formulation of the System for Higher Order Estimates

In this subsection we describe our approach for deriving higher order energy estimates. Our estimates will heavily involve the quantity

34 $$\begin{aligned} \eta \equiv \eta (\partial _x V) := \frac{\partial _{x}V}{v_{\epsilon }-1}. \end{aligned}$$

The evolution equation for $\eta$ reads as

35 $$\begin{aligned} \begin{aligned} \partial _{t}\eta - \partial _{x}(\phi _{\epsilon }(v_{\epsilon }) \partial _{x}\eta )&= \frac{\partial _{x}^{2}W_0}{v_{\epsilon }-1} + \frac{\partial _{x}^{2}H}{v_{\epsilon }-1} + \frac{s\eta v_{\epsilon }'}{v_{\epsilon }-1}\\  &+ \frac{\phi _{\epsilon }(v_{\epsilon })v_{\epsilon }' \partial _{x}^{2}V}{(v_{\epsilon }-1)^{2}} + \partial _{x} \left( \frac{\eta \phi _{\epsilon } (v_{\epsilon }) v_{\epsilon }'}{v_{\epsilon }-1} \right) + \frac{1}{v_{\epsilon }-1} \partial _{x} \left( \phi _{\epsilon }'(v_{\epsilon })v_{\epsilon }' \partial _{x}V \right) , \end{aligned} \end{aligned}$$

which may be expressed as

36 $$\begin{aligned} \partial _{t} \eta - \partial _{x}(\phi _{\epsilon }(v_{\epsilon })\partial _{x}\eta ) =\mathcal {L}_{\epsilon }(\eta ) + S_\epsilon (\partial _x V), \end{aligned}$$

where

$$\begin{aligned}&\mathcal {L}_{\epsilon }(\eta ) := \frac{s\eta v_{\epsilon }'}{v_{\epsilon }-1} + \frac{\phi _{\epsilon }(v_{\epsilon })v_{\epsilon }'}{v_{\epsilon }-1}\left( \partial _{x}\eta + \frac{\eta v_{\epsilon }'}{v_{\epsilon }-1} \right) + \partial _{x}\left( \frac{\eta \phi _{\epsilon }(v_{\epsilon })v_{\epsilon }'}{v_{\epsilon }-1}\right) + \frac{1}{v_{\epsilon }-1}\partial _{x} \left( \phi _{\epsilon }'(v_{\epsilon })v_{\epsilon }' (v_{\epsilon }-1) \eta \right) , \\&S_\epsilon (\partial _x V) := \frac{\partial _{x}^{2}[W_0+H(\partial _x V)]}{v_{\epsilon }-1}. \end{aligned}$$

Differentiating (36), we obtain

37 $$\begin{aligned} \partial _{t}(\partial _{x}\eta ) - \partial _{x}(\phi _{\epsilon }(v_{\epsilon })\partial _{x}^{2}\eta ) = \partial _{x}S_\epsilon (\partial _x V) +\mathcal {L}_{\epsilon }(\partial _{x}\eta ) + \mathcal {C}_\epsilon (\eta ) , \end{aligned}$$

where $\mathcal {C}_\epsilon (\eta ) := [\partial _x,\partial _x(\phi _\epsilon \partial _x\cdot )]\eta + [\partial _x,\mathcal {L}_\epsilon ]\eta = \partial _{x}(\partial _{x}\phi _{\epsilon }(v_{\epsilon })\partial _{x}\eta ) + [\partial _x,\mathcal {L}_\epsilon ] \eta$ appears due to commutators and

$$\begin{aligned} \begin{aligned} {[}\partial _x,\mathcal {L}_\epsilon ] \eta&:= s \eta \partial _{x} \left( \frac{v_{\epsilon }'}{v_{\epsilon }-1} \right) + \partial _{x}\left( \frac{\phi _{\epsilon }(v_{\epsilon })v_{\epsilon }'}{v_{\epsilon }-1}\right) \partial _{x}\eta \\  &+ \partial _{x} \left( \phi _{\epsilon }(v_{\epsilon }) (v_{\epsilon }')^{2} \frac{1}{(v_{\epsilon }-1)^{2}}\right) \eta + \partial _{x} \left( \eta \partial _{x}\left( \frac{\phi _{\epsilon }(v_{\epsilon })v_{\epsilon }'}{v_{\epsilon }-1} \right) \right) \\  &- \frac{v_{\epsilon }'}{(v_{\epsilon }-1)^{2}} \partial _{x} (\phi _{\epsilon }'v_{\epsilon }'(v_{\epsilon }-1)\eta ) + \frac{1}{v_{\epsilon }-1} \partial _{x} (\partial _{x}(\phi _{\epsilon }'(v_{\epsilon })v_{\epsilon }' (v_{\epsilon }-1))\eta ). \end{aligned} \end{aligned}$$

The equations (36) and (37) will be used to derive key estimates that allow us to eventually conclude the fixed-point argument.

Note that the operators $\mathcal {L}_\epsilon$ and $\mathcal {C}_\epsilon$, that appear in equations (36) and (37), are linear. In order to improve readability, we give bounds for these two operators in the following lemma.

#### Lemma 3.3

There exists $C=C(\gamma ,s,v_+)>0$ such that, for every $\alpha \in (0,1)$, for every $\epsilon >0$, for any $\eta$ sufficiently regular, there holds

38 $$\begin{aligned} \left| \int _0^t\int _{\mathbb {R}}\mathcal {L}_\epsilon (\eta )\eta \right| \le \alpha \int _0^t\int _{\mathbb {R}}\phi _\epsilon (v_\epsilon )(\partial _x\eta )^2 + \frac{C}{\alpha \epsilon ^2} \int _0^t\int _{\mathbb {R}}\phi _\epsilon (v_\epsilon )(\partial _x V)^2 \end{aligned}$$

and

39 $$\begin{aligned} \left| \int _0^t\int _{\mathbb {R}}\mathcal {C}_\epsilon (\eta )\partial _x\eta \right| \le \alpha \int _0^t\int _{\mathbb {R}}\phi _\epsilon (v_\epsilon )(\partial _x^2\eta )^2 + \frac{C}{\alpha \epsilon ^2} \int _0^t\int _{\mathbb {R}}\phi _\epsilon (v_\epsilon )\left( (\partial _x\eta )^2+\frac{(\partial _x V^2)}{\epsilon ^2}\right) . \end{aligned}$$

We defer the proof of this lemma to Appendix B.

#### Remark 3.4

Lemma 3.3 is crucial to obtain global in time estimates, as it enables to control the linear operators $\mathcal {L}_\epsilon$ and $\mathcal {C}_\epsilon$ for any positive time with the help of the viscous dissipation. Note that both estimate (38) and (39) strongly rely on the norm of the integrated variables. However, if one only wanted to obtain a local in time well-posedness result, there is no need to assume that the perturbation is integrable. Indeed, by performing integration by parts, one can obtain the following alternative bounds, that do not rely on the integrated variables:

40 $$\begin{aligned}&\left| \int _{\mathbb {R}}\mathcal {L}_\epsilon (\eta )\eta \right| \le \frac{C}{\epsilon }\int _{\mathbb {R}}\eta ^2, \end{aligned}$$

41 $$\begin{aligned}&\left| \int _{\mathbb {R}}\mathcal {C}_\epsilon (\eta )\partial _x\eta \right| \le \frac{C}{\epsilon }\int _{\mathbb {R}}|\partial _x\eta |^2 + \frac{C}{\epsilon ^3}\int _{\mathbb {R}}\eta ^2, \end{aligned}$$

where $C=C(s,\gamma ,v_+)$. Estimates (40) and (41) coupled to a Gronwall type lemma are then enough to obtain a local in time result on a short time interval (see Remark 3.13).

### Construction of Global Strong Solutions

In this subsection we lay out the framework for our fixed point argument before finally stating the result of this section, i.e. the well posedness of (32) in an appropriate space. Firstly, we fix $T>0$ arbitrary. For $t\in [0,T]$, we define the energies

42 $$\begin{aligned} E_{0}(t; V) := \int _{\mathbb {R}} V^{2}(t)~dx, \quad D_{0}(t;V) := \int _{\mathbb {R}} \phi _{\epsilon }(v_{\epsilon })|\partial _{x}V(t)|^{2}~dx, \end{aligned}$$

as well as

43 $$\begin{aligned} \begin{aligned} E_{k}(t;V) := \int _{\mathbb {R}} |\partial _{x}^{k-1}\eta |^{2}(t)~dx, \quad D_{k}(t;V) := \int _{\mathbb {R}} \phi _{\epsilon }(v_{\epsilon }) |\partial _{x}^{k}\eta |^{2}(t)~dx, \quad \text { for } k=1,2. \end{aligned} \end{aligned}$$

Then given $V_{1}$, we introduce the system

44 $$\begin{aligned} \left\{ \begin{aligned}&\partial _t V_{2} -\partial _x W_0 -\partial _x(\phi _\epsilon (v_\epsilon )\partial _xV_{2})=\partial _x H(\partial _xV_{1}),\\&V_{2}|_{t=0} = V_{0}, \end{aligned} \right. \end{aligned}$$

and the application $\mathcal {A}^{\epsilon } : V_{1} \mapsto V_{2},$ where $V_{1}, V_{2} \in \mathcal {X}$ and

45 $$\begin{aligned}  &   \mathcal {X} = \left\{ V : E_{k}(t, V) \in L^{\infty }(0,T) \text { and } D_{k}(t; V) \in L^{1}(0,T)~ \text { for } k=0,1,2. \right\} ,\nonumber \\  &   \Vert V\Vert _{\mathcal {X}}^{2} := \sup _{t \in [0,T]} \left( \sum _{k=0}^{2}c_{k} \epsilon ^{2k} \left[ E_{k}(t; V(t)) + \int _{0}^{t}D_{k}(\tau ; V(\tau ))~d\tau \right] \right) , \end{aligned}$$

where $c_{k}=c_{k}(s,\gamma , v_{+})$, for $k=0,1,2$, are constants independent of $\epsilon$ which are to be determined. For a proof that the map $\mathcal {A}^\epsilon$ is well defined, see Appendix A. For $\delta > 0$ we define the ball

46 $$\begin{aligned} B_{\delta } := \left\{ V \in \mathcal {X} : \Vert V\Vert _{\mathcal {X}} < \delta \epsilon ^{3/2} \right\} . \end{aligned}$$

The remainder of this section aims to prove the following result.

#### Proposition 3.5

There exists constants $\delta _0,\delta$ depending only on $(s,\gamma , v_+)$ such that the following statement holds. Assume that the initial data satisfies

47 $$\begin{aligned} \begin{aligned} \sum _{k=0}^2 \left( c_k \epsilon ^{2k} E_{k}(0; V_{0})+\epsilon ^{2k-1}\Vert \sqrt{x}\partial _x^k W_0\Vert _{L^2(\mathbb {R}_+)}^2\right) + \Vert W_0\Vert _{L^2_x}^2 + \left( \frac{T}{\epsilon }\right) ^{1/\gamma }\left( \epsilon ^2\Vert \partial _x W_0\Vert ^2_{L^2_x}+\epsilon ^{4} \Vert \partial _{x}^{2}W_{0}\Vert _{L^{2}_{x}}^{2}\right) \le \delta _0 \epsilon ^3, \end{aligned} \end{aligned}$$

where $c_{k} = c_{k}(s,\gamma , v_{+})$ for $k=0,1,2$ are positive constants. Then

1. the ball $B_{\delta }$ is stable by $\mathcal {A}^{\epsilon }$,
2. the map $\mathcal {A}^{\epsilon }$ is a contraction on $B_{\delta }$.

Consequently, $\mathcal {A}^{\epsilon }$ has a unique fixed point in $B_{\delta }$.

### Stability of the Ball $B_{\delta }$

We let $\delta > 0$, $V_{1} \in B_{\delta }$ and $V_{2} := \mathcal {A}^{\epsilon }(V_{1})$. The aim of this subsection is to show that we can find $\delta >0$ such that $V_{2} \in B_{\delta }$. First, let us clarify the meaning of some notation we will use in this section.

- We denote by $C= C(s,\gamma , v_{+})$ an arbitrary positive constant independent of $\epsilon$. This constant may change, even within the same line. We also denote $C' = C'(s,\gamma ,v_+,c_0,c_1,c_2)$ another positive constant that may additionally depend on the constants $c_0,c_1,c_2$ appearing in the definition of the norm $\Vert \cdot \Vert _{\mathcal {X}}$.
- To ease notation, we will often shorten $\phi _{\epsilon }(v_{\epsilon }), \psi _{\epsilon }(v_{\epsilon })$ to $\phi _{\epsilon }, \psi _{\epsilon }$ respectively. For $i=1,2$ we also adopt the notation $\eta _{i} := \frac{\partial _{x}V_{i}}{v_{\epsilon }-1}$.
- We adopt the notation $X_{t}Y_{x} := X(0,T; Y(\mathbb {R}))$ for appropriate function spaces *X* and *Y*.

Next, we make note of some useful estimates which will be repeatedly used in the remainder of the paper. We have up to a constant independent of $\epsilon$,

48 $$\begin{aligned}&\phi _\epsilon (v_\epsilon ) = \frac{\epsilon \gamma }{v_\epsilon (v_\epsilon -1)^{\gamma +1}},\nonumber \\&\phi _{\epsilon }^{(k)}(v_{\epsilon }) \cong \frac{\phi _{\epsilon }(v_{\epsilon })}{(v_{\epsilon }-1)^{k}}, \end{aligned}$$

49 $$\begin{aligned}&\partial _{x}v_{\epsilon } = v_{\epsilon }' ~\cong ~ \frac{1}{\epsilon }(v_{\epsilon }-1)^{\gamma +1}. \end{aligned}$$

As a consequence of (48) and (49), we have $(v_{\epsilon }-1)^{2}\partial _{x}v_{\epsilon } \le C/\epsilon$ and $(v_{\epsilon }-1)^{2}/ \phi _{\epsilon } \le C/\epsilon$, provided $\gamma \ge 1$. Let us now note a Gagliardo-Nirenberg-Sobolev interpolation inequality which we will use. For $f \in H^1(\mathbb {R})$,

50 $$\begin{aligned}&\Vert f\Vert _{L^{\infty }_{x}} \le C\Vert \partial _{x}f\Vert _{L^{2}_{x}}^{\frac{1}{2}} \Vert f\Vert _{L^{2}_{x}}^{\frac{1}{2}}, \end{aligned}$$

This interpolation inequality coupled to the definition of the norm $\Vert \cdot \Vert _{\mathcal {X}}$ (45) yields the estimates stated in the following lemma:

#### Lemma 3.6

For any $V\in \mathcal {X}$, let as before

$$\begin{aligned} \eta := \frac{\partial _xV}{v_\epsilon -1}. \end{aligned}$$

Then the $L^\infty$ norm of $\eta$ is bounded by

51 $$\begin{aligned}&\Vert \eta \Vert _{L^\infty _{t,x}} \le C \Vert \eta \Vert _{L^\infty _tL^2_x}^{1/2}\Vert \partial _x\eta \Vert _{L^\infty _tL^2_x}^{1/2}\le \frac{C'}{\epsilon ^{3/2}}\Vert V\Vert _{\mathcal {X}}. \end{aligned}$$

Finally, we mention some guidelines which our estimates will follow.

- We will often artificially place a factor of $\phi _{\epsilon }$ into the integral in order to obtain an expression which is a function of the energies (42)-(43). This results in the multiplication of a factor of $1/\epsilon$. For example, to estimate the term $A:= \int _{0}^{t} \int _{\mathbb {R}} \partial _{x}V \partial _{x}\eta ~dxd\tau$, we have
  52 $$\begin{aligned} A \le \left\| \frac{1}{\phi _{\epsilon }}\right\| _{L^{\infty }_{t,x}} \int _{0}^{t} \int _{\mathbb {R}} | \sqrt{\phi _{\epsilon }} \partial _{x}V| |\sqrt{\phi _{\epsilon }} \partial _{x}\eta |~dxd\tau \le \frac{C}{\epsilon } \Vert \sqrt{\phi _{\epsilon }} \partial _{x}V\Vert _{L^{2}_{t,x}} \Vert \sqrt{\phi _{\epsilon }} \partial _{x}\eta \Vert _{L^{2}_{t,x}}. \end{aligned}$$
- To estimate terms involving the expression $\partial _{x}^{2}V / (v_{\epsilon }-1)$, we will make use of the identity
  53 $$\begin{aligned} \frac{\partial _{x}^{2}V}{v_{\epsilon }-1} = \partial _{x}\eta + \frac{v_{\epsilon }'\partial _{x}V}{(v_{\epsilon }-1)^{2}}. \end{aligned}$$

#### Estimates for $k=0$

Fix $t \in [0,T]$. We multiply (44) by $V_2$ and integrate on $\mathbb {R}\times (0,t)$ to obtain as before:

54 $$\begin{aligned} \frac{1}{2}\int _{\mathbb {R}}V_2^2(t)dx +\int _0^t\int _{\mathbb {R}}\phi _\epsilon |\partial _x V_2|^2dxd\tau = \frac{1}{2}\int _{\mathbb {R}}V_2^2(0)dx+\int _0^t\int _{\mathbb {R}}V_2(\partial _x H(\partial _x V_1)+\partial _x W_0)dxd\tau . \end{aligned}$$

Integrating by parts, we have

55 $$\begin{aligned} \begin{aligned} \int _{0}^{t}\int _{\mathbb {R}} V_{2} \partial _{x}(H(\partial _{x}V_{1}) + W_0) = - \int _{0}^{t} \int _{\mathbb {R}} H(\partial _{x}V_{1})\partial _{x}V_{2} -\int _{0}^{t} \int _{\mathbb {R}} W_0 \partial _{x}V_2=: G_{1} + G_{2}. \end{aligned} \end{aligned}$$

Using the estimate on *H* and (50), and introducing $\eta _2 = \frac{\partial _x V_2}{v_\epsilon -1}$,

56 $$\begin{aligned} \begin{aligned} |G_{1}|&\le C\int _{0}^{t} \int _{\mathbb {R}} \phi _{\epsilon }(v_{\epsilon }) \frac{|\partial _{x}V_{1}|^{2}}{v_{\epsilon }-1} |\partial _{x}V_{2}| = C \int _{0}^{t}\int _{\mathbb {R}} \phi _{\epsilon }|\partial _{x}V_{1}|^{2} |\eta _{2}| \\&\le C \Vert \eta _{2}\Vert _{L^{\infty }_{t,x}} \Vert \sqrt{\phi _{\epsilon }}\partial _{x}V_{1}\Vert _{L^{2}_{t,x}}^{2} \le C\Vert \eta _2\Vert _{L^{\infty }_{t}L^{2}_{x}}^{1/2} \Vert \partial _{x}\eta _2\Vert _{L^{\infty }_{t}L^{2}_{x}}^{1/2}\Vert \sqrt{\phi _{\epsilon }}\partial _{x}V_{1}\Vert _{L^{2}_{t,x}}^{2} \\&\le \frac{C'}{\epsilon ^{3/2}} \Vert V_{1}\Vert _{\mathcal {X}}^{2}\Vert V_{2}\Vert _{\mathcal {X}}. \end{aligned} \end{aligned}$$

We now estimate $G_2$. Young’s inequality gives that

$$\begin{aligned} \left| \int _{0}^{t} \int _{\mathbb {R}} W_0 \partial _xV_{2}\right| \le \frac{1}{4}\int _{0}^{t} D_{0}(\tau ; V_{2})~d\tau +\int _0^t\int _\mathbb {R}\frac{W_0^2}{\phi _\epsilon }. \end{aligned}$$

Using Fubini and the fact that $W_0$ is independant of time, one then has

$$\begin{aligned} \int _0^t\int _\mathbb {R}\frac{W_0^2}{\phi _\epsilon } = \int _\mathbb {R}W_0^2\int _0^t\frac{1}{\phi _\epsilon } = \int _\mathbb {R}W_0^2\int _0^t\frac{1}{\phi _\epsilon } \textbf{1}_{\xi <0}\,d\tau dx + \int _\mathbb {R}W_0^2\int _0^t\frac{1}{\phi _\epsilon } \textbf{1}_{\xi >0}\,d\tau dx, \end{aligned}$$

where $\xi := x-s\tau$. By using Equation (10), one then obtain for the first integral the estimate

$$\begin{aligned} \int _\mathbb {R}W_0^2\int _0^t\frac{1}{\phi _\epsilon } \textbf{1}_{\xi<0}\,d\tau dx \le \frac{C}{\epsilon }\int _\mathbb {R}W_0^2\int _0^{+\infty }\left( B-\frac{A_1}{\epsilon }\xi \right) ^{-1-1/\gamma } \textbf{1}_{\xi <0}\,d\tau dx \le C\int _{\mathbb {R}}W_0^2\,dx, \end{aligned}$$

and for the second integral

$$\begin{aligned} \int _\mathbb {R}W_0^2\int _0^t\frac{1}{\phi _\epsilon } \textbf{1}_{\xi>0}\,d\tau dx \le \frac{C}{\epsilon }\int _\mathbb {R}W_0^2\int _0^{x/s}\textbf{1}_{\xi >0}d\tau dx=\frac{C}{\epsilon }\int _{\mathbb {R}}W_0^2 x \textbf{1}_{x\ge 0}\,dx. \end{aligned}$$

Returning to (54), we get

$$\begin{aligned}&\int _{\mathbb {R}} V^{2}_2(t)~dx + \frac{3}{2}\int _{0}^{t} \int _{\mathbb {R}} \phi _{\epsilon }(v_{\epsilon }) |\partial _{x}V_2|^{2}~dxd\tau \\ \le&\int _{\mathbb {R}}V^{2}_2(0)~dx + C \Vert W_{0}\Vert _{L^{2}_{x}}^{2} +\frac{C}{\epsilon }\Vert \sqrt{x}W_0\Vert _{L^2(\mathbb {R}_+)}+ \frac{C'}{\epsilon ^{3/2}} \Vert V_{1}\Vert _{\mathcal {X}}^{2}\Vert V_{2}\Vert _{\mathcal {X}}, \end{aligned}$$

i.e.

57 $$\begin{aligned} E_{0}(t; V_{2}) + \frac{3}{2}\int _{0}^{t}D_{0}(\tau ;V_{2})~d\tau \le E_{0}(0; V_{2}) + C \Vert W_0\Vert _{L^{2}_{x}}^{2}+\frac{C}{\epsilon }\Vert \sqrt{x}W_0\Vert ^2_{L^2(\mathbb {R}_+)} + \frac{C'}{\epsilon ^{3/2}} \Vert V_{1}\Vert _{\mathcal {X}}^{2}\Vert V_{2}\Vert _{\mathcal {X}} . \end{aligned}$$

##### Remark 3.7

Note that (57) does not depend on $T>0$. This is due to the assumption that $\sqrt{x}W_0\in L^2(\mathbb {R}_+)$. If this assumption is removed, one can still obtain a bound, but it is not global in time anymore. Indeed, we can simply apply Holder and Young’s inequality to get

$$\begin{aligned} \begin{aligned} \left| \int _{0}^{t} \int _{\mathbb {R}} W_0 \partial _{x}V_{2} \right|&\le \Vert \phi _{\epsilon }^{-1/2}\Vert _{L^{\infty }_{t,x}} \Vert \sqrt{\phi _{\epsilon }} \partial _{x}V_{2}\Vert _{L^{2}_{t,x}} \Vert W_0\Vert _{L^{2}_{t,x}} \le \frac{Ct}{\epsilon } \Vert W_{0}\Vert _{L^{2}_{x}}^{2} + \frac{1}{4} \int _{0}^{t} D_{0}(\tau ; V_{2})~d\tau . \end{aligned} \end{aligned}$$

Thus instead of (57) we obtain

58 $$\begin{aligned} E_{0}(t; V_{2}) + \frac{3}{2}\int _{0}^{t}D_{0}(\tau ;V_{2})~d\tau \le E_{0}(0; V_{2}) + \frac{Ct}{\epsilon }\Vert W_{0}\Vert _{L^{2}_{x}}^{2} + \frac{C'}{\epsilon ^{3/2}} \Vert V_{1}\Vert _{\mathcal {X}}^{2}\Vert V_{2}\Vert _{\mathcal {X}} . \end{aligned}$$

#### Estimates for $k=1$

From (36), we deduce that $\eta _2$ solves

$$\begin{aligned} \partial _{t} \eta _2 - \partial _{x}(\phi _{\epsilon }(v_{\epsilon })\partial _{x}\eta _2) =\mathcal {L}_{\epsilon }(\eta _2) + S_\epsilon (\partial _x V_1). \end{aligned}$$

Multiplying by $\eta _{2}$ and integrating in space and time, we get

59 $$\begin{aligned} \begin{aligned} \frac{1}{2}\int _{\mathbb {R}} |\eta _{2}(t)|^{2}~dx&+ \int _{0}^{t} \int _{\mathbb {R}} \phi _{\epsilon }(v_{\epsilon })|\partial _{x}\eta _{2}|^{2}~dxd\tau - \frac{1}{2}\int _{\mathbb {R}} |\eta _{2}(0)|^{2}~dx \\  &= \int _{0}^{t}\int _{\mathbb {R}} \eta _{2} \frac{\partial _{x}^{2}H(\partial _{x}V_{1})}{v_{\epsilon }-1}~dxd\tau + \int _{0}^{t} \int _{\mathbb {R}} \eta _{2} \frac{\partial _{x}^{2}W_0}{v_{\epsilon }-1}~dxd\tau + \int _{0}^{t}\int _{\mathbb {R}} \mathcal {L}_\epsilon (\eta _2)\eta _2 \,\,dxd\tau \\&=: \sum _{n=1}^{3}I_{n}. \end{aligned} \end{aligned}$$

We now estimate each of $I_{1}, I_2 , I_{3}$. For $I_{1}$, we first integrate by parts to get

$$\begin{aligned} I_{1} = - \int _{0}^{t} \int _{\mathbb {R}} \partial _{x}\eta _{2} \frac{\partial _{x}H(\partial _{x}V_{1})}{v_{\epsilon }-1} + \int _{0}^{t}\int _{\mathbb {R}} \eta _{2} \frac{v_{\epsilon }' \partial _{x}H(\partial _{x}V_{1})}{(v_{\epsilon }-1)^{2}} =: (1a) + (1b). \end{aligned}$$

Using (30),

$$\begin{aligned} \begin{aligned} |(1a)|&\le C\int _{0}^{t} \int _{\mathbb {R}} |\partial _{x} \eta _{2}| \left| \frac{1}{v_{\epsilon }-1} \right| \left( \left| \frac{\partial _{x}V_{1}}{v_{\epsilon }-1} \right| ^{2} + \frac{\epsilon }{(v_{\epsilon }-1)^{\gamma +2}}|\partial _{x}V_{1}| |\partial _{x}^{2}V_{1}| \right) . \end{aligned} \end{aligned}$$

The first term can be estimated as

$$\begin{aligned} \begin{aligned} \int _{0}^{t} \int _{\mathbb {R}} |\partial _{x} \eta _{2}| \left| \frac{1}{v_{\epsilon }-1} \right| \left| \frac{\partial _{x}V_{1}}{v_{\epsilon }-1} \right| ^{2}&\le \left\| \frac{1}{\phi _{\epsilon }(v_{\epsilon }-1)^{2}}\right\| _{L^{\infty }_{t,x}} \Vert \eta _{1}\Vert _{L^{\infty }_{t,x}} \Vert \sqrt{\phi _{\epsilon }}\partial _{x}V_{1}\Vert _{L^{2}_{t,x}} \Vert \sqrt{\phi _{\epsilon }} \partial _{x} \eta _{2}\Vert _{L^{2}_{t,x}} \\  &\le \frac{C'}{\epsilon ^{7/2}} \Vert V_{1}\Vert _{\mathcal {X}}^{2} \Vert V_{2}\Vert _{\mathcal {X}}, \end{aligned} \end{aligned}$$

where we have used (50). Note that the identity (53) implies that

60 $$\begin{aligned} \begin{aligned} \left\| \sqrt{\phi _{\epsilon }}\frac{\partial _{x}^{2}V_{1}}{v_{\epsilon }-1} \right\| _{L^{2}_{t,x}}&\le \Vert \sqrt{\phi _{\epsilon }} \partial _{x}\eta _{1} \Vert _{L^{2}_{t,x}} + \Vert v_{\epsilon }'(v_{\epsilon }-1)^{-2} \sqrt{\phi _{\epsilon }} \partial _{x}V_{1}\Vert _{L^{2}_{t,x}} \le \frac{C'}{\epsilon }\Vert V_{1}\Vert _{\mathcal {X}}. \end{aligned} \end{aligned}$$

Therefore, we have

$$\begin{aligned} \begin{aligned} \int _{0}^{t}\int _{\mathbb {R}} \frac{\epsilon }{(v_{\epsilon }-1)^{\gamma +1}}|\partial _{x}\eta _{2}| \left| \frac{\partial _{x}^{2}V_{1}}{v_{\epsilon }-1}\right| \left| \frac{\partial _{x}V_{1}}{v_{\epsilon }-1}\right|&\le \left\| \frac{\epsilon }{(v_\epsilon -1)^{\gamma +1}\phi _\epsilon }\right\| _{L^\infty _{t,x}}\Vert \eta _1\Vert _{L^\infty _{t,x}} \left\| \sqrt{\phi _\epsilon }\partial _x\eta _2\right\| _{L^2_{t,x}}\left\| \sqrt{\phi _\epsilon }\frac{\partial _x^2 V_1}{v_\epsilon -1}\right\| _{L^2_{t,x}}\\&\le \frac{C'}{\epsilon ^{7/2}} \Vert V_{1}\Vert _{\mathcal {X}}^{2}\Vert V_{2}\Vert _{\mathcal {X}}. \end{aligned} \end{aligned}$$

Next note that using (30),

$$\begin{aligned} |(1b)| \le \,&\int _{0}^{t} \int _{\mathbb {R}} \frac{v_{\epsilon }'}{(v_{\epsilon }-1)^3} |\partial _{x}V_{2} | |\partial _{x}H(\partial _{x}V_{1})| \\ \le \,&\int _{0}^{t} \int _{\mathbb {R}} | \partial _{x}V_{2} |\frac{v_{\epsilon }'}{(v_{\epsilon }-1)^3 } \left( \left| \frac{\partial _{x}V_{1}}{v_{\epsilon }-1} \right| ^{2} + \frac{\epsilon }{(v_{\epsilon }-1)^{\gamma +2}}|\partial _{x}V_{1}| |\partial _{x}^{2}V_{1}| \right) . \end{aligned}$$

Then estimating in the same way as (1*a*), we also find that $(1b) \le (C'/\epsilon ^{7/2}) \Vert V_{1}\Vert _{\mathcal {X}}^{2}\Vert V_{2}\Vert _{\mathcal {X}}$ and so

61 $$\begin{aligned} |I_{1}| \le \frac{C'}{\epsilon ^{7/2}} \Vert V_{1}\Vert _{\mathcal {X}}^{2}\Vert V_{2}\Vert _{\mathcal {X}}. \end{aligned}$$

Integrating by parts,

$$\begin{aligned} \begin{aligned} I_{2} = - \int _{0}^{t} \int _{\mathbb {R}} \frac{\partial _{x}W_0}{v_{\epsilon }-1} \partial _{x}\eta _{2} + \int _{0}^{t}\int _{\mathbb {R}} \frac{v_{\epsilon }' \partial _{x}W_0}{(v_{\epsilon }-1)^{2}} \frac{\partial _{x}V_{2}}{v_{\epsilon }-1}. \end{aligned} \end{aligned}$$

We first compute using Young’s inequality that

$$\begin{aligned} \left| \int _0^t\int _{\mathbb {R}}\frac{\partial _x W_0}{v_\epsilon -1}\partial _x\eta _2\right| \le \frac{1}{8}\int _0^tD_1(\tau ; V_2)\,d\tau + C\int _0^t\int _{\mathbb {R}}\frac{1}{\phi _\epsilon }\left( \frac{\partial _x W_0}{v_\epsilon -1}\right) ^2. \end{aligned}$$

The last integral can be estimated by

$$\begin{aligned} \int _0^t\int _{\mathbb {R}}\frac{1}{\phi _\epsilon }\left( \frac{\partial _x W_0}{v_\epsilon -1}\right) ^2 \le \int _{\mathbb {R}}(\partial _x W_0)^2\int _0^t\frac{1}{\phi _\epsilon (v_\epsilon -1)^2}\left( \textbf{1}_{\xi <0}+\textbf{1}_{\xi >0}\right) d\tau dx, \end{aligned}$$

with $\xi :=x-s\tau$. The bound (10) then yields for the part $\{\xi <0\}$ the estimate

$$\begin{aligned} \int _0^t\frac{1}{\phi _\epsilon (v_\epsilon -1)^2}\textbf{1}_{\xi<0} \le&\frac{C}{\epsilon }\int _0^t\left( B-\frac{A_1}{\epsilon }\xi \right) ^{-1+1/\gamma }\textbf{1}_{\xi <0}\\ \le&C\left[ \left( B-\frac{A_1}{\epsilon }(x-st)\right) ^{1/\gamma }-\left( B-\frac{A_1}{\epsilon }(x-\max (x,0))\right) ^{1/\gamma }\right] \textbf{1}_{x-st\le 0}. \end{aligned}$$

Note that the bound obtained in the end is a positive nondecreasing function that goes to $+\infty$ as *t* goes to $+\infty$, for every *x*. The inequality (8) shows that this bound is optimal, i.e. that a similar lower bounds holds for this integral. Hence it seems complicated to bound the contribution of the integral $I_2$ uniformly in time. However, it is always possible to write a bound of the form

$$\begin{aligned} \int _0^t\frac{1}{\phi _\epsilon (v_\epsilon -1)^2}\textbf{1}_{\xi <0} \le C\left( \frac{t}{\epsilon }\right) ^{1/\gamma }, \end{aligned}$$

uniformly in *x*. For the part $\{\xi >0\}$, one has as before

$$\begin{aligned} \int _\mathbb {R}(\partial _x W_0)^2\int _0^t\frac{1}{\phi _\epsilon (v_\epsilon -1)^2} \textbf{1}_{\xi>0}\,d\tau dx \le \frac{C}{\epsilon }\int _\mathbb {R}(\partial _x W_0)^2\int _0^{x/s}\textbf{1}_{\xi >0}d\tau dx=\frac{C}{\epsilon }\int _{\mathbb {R}}(\partial _x W_0)^2 x \textbf{1}_{x\ge 0}\,dx. \end{aligned}$$

For the second integral appearing in $I_2$, we use the same splitting between $\xi <0$ and $\xi >0$, together with (10) to obtain again

$$\begin{aligned} \left| \int _{0}^{t}\int _{\mathbb {R}} \frac{v_{\epsilon }' \partial _{x}W_0}{(v_{\epsilon }-1)^{2}} \frac{\partial _{x}V_{2}}{v_{\epsilon }-1}\right| \le \frac{1}{2\epsilon ^2}\int _0^t D_0(\tau ,V_2)d\tau + C\left( \frac{t}{\epsilon }\right) ^{1/\gamma }\Vert \partial _x W_0\Vert ^2_{L^2_x} + \frac{C}{\epsilon }\Vert \sqrt{x}\partial _x W_0\Vert _{L^2(\mathbb {R}_+)}^2. \end{aligned}$$

This leads to

62 $$\begin{aligned} |I_2| \le \frac{1}{8}\int _0^tD_1(\tau ;V_2)d\tau +\frac{1}{2\epsilon ^2}\int _0^tD_0(\tau ;V_2)d\tau +C\left( \frac{t}{\epsilon }\right) ^{1/\gamma }\Vert \partial _x W_0\Vert ^2_{L^2_x} + \frac{C}{\epsilon }\Vert \sqrt{x}\partial _x W_0\Vert _{L^2(\mathbb {R}_+)}^2. \end{aligned}$$

By comparison with (57), we see that multiplicating by the singular weight $1/(v_\epsilon -1)$ prevents the estimates from being uniform in time when $W_0\ne 0$.

##### Remark 3.8

If we do not suppose that $\sqrt{x}\partial _x W_0 \in L^2(\mathbb {R}_+)$, but only that $W_0\in H^1(\mathbb {R})$, it is still possible to obtain a weaker estimate:

$$\begin{aligned} \begin{aligned} |I_{2}|&\le \left\| \frac{1}{(v_{\epsilon }-1)\sqrt{\phi _{\epsilon }}}\right\| _{L^{\infty }_{t,x}} \Vert \sqrt{\phi _{\epsilon }} \partial _{x}\eta _{2}\Vert _{L^{2}_{t,x}} \Vert \partial _{x}W_0\Vert _{L^{2}_{t,x}} + \left\| \frac{v_{\epsilon }'}{(v_{\epsilon }-1)^{3}\sqrt{\phi _{\epsilon }}}\right\| _{L^{\infty }_{t,x}}\Vert \sqrt{\phi _{\epsilon } }\partial _{x}V_{2}\Vert _{L^{2}_{t,x}} \Vert \partial _{x}W_0\Vert _{L^{2}_{t,x}} \\  &\le \frac{Ct}{\epsilon } \Vert \partial _{x}W_{0}\Vert _{L^{2}_{x}}^{2} + \frac{C}{\epsilon ^{2}} \int _{0}^{t}D_{0}(\tau ;V_{2})~d\tau + \frac{1}{8} \int _{0}^{t} D_{1}(\tau ;V_{2})~d\tau . \end{aligned} \end{aligned}$$

Finally, we use Equation (38) of Lemma 3.3 with $\alpha =1/8$ in order to bound $I_3$:

$$\begin{aligned} |I_3| = \left| \int _0^t\int _{\mathbb {R}}\mathcal {L}_\epsilon (\eta _2)\eta _2 \right| \le \frac{1}{8} \int _0^t\int _{\mathbb {R}}\phi _\epsilon (\partial _x\eta _2)^2 + \frac{C}{\epsilon ^2} \int _0^t\int _{\mathbb {R}}\phi _\epsilon (\partial _x V_2)^2 \end{aligned}$$

Collecting our estimates for $I_{1}-I_{3}$ and returning to (59), we find

63 $$\begin{aligned} \begin{aligned} E_{1}(t;V_{2}) + \frac{3}{2}\int _{0}^{t} D_{1}(\tau ;V_{2}) ~ d\tau&\le E_{1}(0;V_{2}) + C\left( \frac{t}{\epsilon }\right) ^{1/\gamma } \Vert \partial _{x}W_{0}\Vert _{L^{2}_{x}}^{2} +\frac{C}{\epsilon }\Vert \sqrt{x}\partial _x W_0\Vert ^2_{L^2(\mathbb {R}_+)} \\  &~~~ + \frac{C'}{\epsilon ^{7/2}} \Vert V_{1}\Vert _{\mathcal {X}}^{2}\Vert V_{2}\Vert _{\mathcal {X}} + \frac{C}{\epsilon ^{2}}\int _{0}^{t}D_{0}(\tau ;V_{2})~d\tau , \end{aligned} \end{aligned}$$

which yields, after multiplication by $\epsilon ^2$,

64 $$\begin{aligned} \begin{aligned} \epsilon ^2 E_{1}(t;V_{2}) + \frac{3}{2}\epsilon ^2\int _{0}^{t} D_{1}(\tau ;V_{2}) ~ d\tau&\le \epsilon ^2 E_{1}(0;V_{2}) + C\left( \frac{t}{\epsilon }\right) ^{1/\gamma } \epsilon ^2\Vert \partial _{x}W_{0}\Vert _{L^{2}_{x}}^{2} +C\epsilon \Vert \sqrt{x}\partial _x W_0\Vert ^2_{L^2(\mathbb {R}_+)} \\  &~~~ + \frac{C'}{\epsilon ^{3/2}} \Vert V_{1}\Vert _{\mathcal {X}}^{2}\Vert V_{2}\Vert _{\mathcal {X}} + C\int _{0}^{t}D_{0}(\tau ;V_{2})~d\tau . \end{aligned} \end{aligned}$$

##### Remark 3.9

If we do not suppose that $\sqrt{x}\partial _x W_0$ belong to $L^2(\mathbb {R}_+)$, we still have

$$\begin{aligned} \begin{aligned} \epsilon ^{2} E_{1}(t;V_{2}) + \frac{3}{2}\epsilon ^{2} \int _{0}^{t} D_{1}(\tau ;V_{2})~d\tau \le&\epsilon ^{2}E_{1}(0;V_{2}) + \frac{C'}{\epsilon ^{3/2}} \Vert V_{1}\Vert _{\mathcal {X}}^{2}\Vert V_{2}\Vert _{\mathcal {X}} + Ct\epsilon \Vert \partial _{x}W_{0}\Vert _{L^{2}_{t,x}}^{2} . \end{aligned} \end{aligned}$$

#### Estimates for $k=2$

Equation (37) gives that $\partial _x \eta _2$ solves

$$\begin{aligned} \partial _{t}(\partial _{x}\eta _2) - \partial _{x}(\phi _{\epsilon }(v_{\epsilon })\partial _{x}^{2}\eta _2) = \partial _{x}S_\epsilon (\partial _x V_1) +\mathcal {L}_{\epsilon }(\partial _{x}\eta _2) + \mathcal {C}_\epsilon (\eta _2). \end{aligned}$$

Multiplying by $\partial _{x}\eta _{2}$ and integrating in space and time leads to

65 $$\begin{aligned} \begin{aligned} \frac{1}{2}\int _{\mathbb {R}} |\partial _{x}\eta _{2}(t)|^{2}~dx&+ \int _{0}^{t} \int _{\mathbb {R}} \phi _{\epsilon }(v_{\epsilon }) |\partial _{x}^{2}\eta _{2}|^{2}~dxd\tau - \frac{1}{2}\int _{\mathbb {R}} |\partial _{x}\eta _{2}(0)|^{2}~dx \\ =&\int _{0}^{t}\int _{\mathbb {R}} \frac{\partial _{x}^{2}H(\partial _x V_1)}{v_{\epsilon }-1} \partial _{x}^{2}\eta _{2} + \int _{0}^{t}\int _{\mathbb {R}} \frac{\partial _{x}^{2}W_0}{v_{\epsilon }-1} \partial _{x}^{2}\eta _{2} + \int _{0}^{t}\mathcal {L}_\epsilon (\partial _x\eta _2)\partial _x \eta _2 + \int _{0}^{t}\int _{\mathbb {R}} \partial _{x}\eta _{2} ~ \mathcal {C}_\epsilon (\eta _{2}) \\ =&: \sum _{n=1}^{4}J_{n}. \end{aligned} \end{aligned}$$

We now estimate $J_{1}, J_2, J_{3}, J_4$. Using (31),

66 $$\begin{aligned} \begin{aligned} |J_{1}|&\le C \int _{0}^{t}\int _{\mathbb {R}} \frac{|\partial _{x}^{2}\eta _{2}|}{v_{\epsilon }-1} \left( \frac{(v_{\epsilon }-1)^{\gamma -2}}{\epsilon } |\partial _{x}V_{1}|^{2} + \frac{\epsilon }{(v_{\epsilon }-1)^{\gamma +2}}|\partial _{x}V_{1}| |\partial _{x}^{3}V_{1}| + \frac{\epsilon }{(v_{\epsilon }-1)^{\gamma +2}}|\partial _{x}^{2}V_{1}|^{2} \right) \\  &\le \frac{C}{\epsilon ^{2}} \Vert \eta _{1}\Vert _{L^{\infty }_{t,x}}\Vert \sqrt{\phi _{\epsilon }} \partial _{x}^{2}\eta _{2}\Vert _{L^{2}_{t,x}} \Vert \sqrt{\phi _{\epsilon }} \partial _{x}V_{1}\Vert _{L^{2}_{t,x}} + C\epsilon \int _{0}^{t} \int _{\mathbb {R}} \frac{|\partial _{x}V_{1}|}{v_{\epsilon }-1} \frac{|\partial _{x}^{2}\eta _{2}|}{(v_{\epsilon }-1)^{\gamma +2}}|\partial _{x}^{3}V_{1}| \\  &~~~+ C\epsilon \int _{0}^{t} \int _{\mathbb {R}} \frac{|\partial _{x}^{2}\eta _{2}|}{v_{\epsilon }-1} \frac{ |\partial _{x}^{2}V_{1}|^{2}}{(v_{\epsilon }-1)^{\gamma +2}}. \end{aligned} \end{aligned}$$

We have that

$$\begin{aligned} \begin{aligned} \epsilon \int _{0}^{t} \int _{\mathbb {R}} \frac{|\partial _{x}V_{1}|}{v_{\epsilon }-1} \frac{|\partial _{x}^{2}\eta _{2}|}{(v_{\epsilon }-1)^{\gamma +2}}|\partial _{x}^{3}V_{1}|&\le C\Vert \eta _{1}\Vert _{L^{\infty }_{t,x}} \int _{0}^{t} \int _{\mathbb {R}} \frac{|\partial _{x}^{3}V_{1}|}{v_{\epsilon }-1} \phi _{\epsilon } |\partial _{x}^{2}\eta _{2}| \\  &\le C \Vert \eta _{1}\Vert _{L^{\infty }_{t,x}} \left\| \sqrt{\phi _{\epsilon }}\partial _{x}^{2}\eta _{2}\right\| _{L^{2}_{t,x}} \left\| \sqrt{\phi _{\epsilon }}\frac{\partial _{x}^{3}V_{1}}{v_{\epsilon }-1}\right\| _{L^{2}_{t,x}}. \end{aligned} \end{aligned}$$

We compute that

$$\begin{aligned} \frac{\partial _{x}^{3}V_{1}}{v_{\epsilon }-1} = \partial _{x}^{2}\eta _{1} + \frac{2( v_{\epsilon }')}{v_{\epsilon }-1} \partial _{x}\eta _{1} + \frac{\partial _x V_1}{v_\epsilon -1}\left[ \frac{(v_\epsilon ')^2}{(v_\epsilon -1)^2}+\partial _x\left( \frac{v_\epsilon '}{v_\epsilon -1}\right) \right] , \end{aligned}$$

hence

67 $$\begin{aligned} \left\| \sqrt{\phi _{\epsilon }}\frac{\partial _{x}^{3}V_{1}}{v_{\epsilon }-1}\right\| _{L^{2}_{t,x}} \le \frac{C'}{\epsilon ^2}\Vert V_1\Vert _{\mathcal {X}}, \end{aligned}$$

which yields with the previous computations that

$$\begin{aligned} \epsilon \int _{0}^{t} \int _{\mathbb {R}} \frac{|\partial _{x}V_{1}|}{v_{\epsilon }-1} \frac{|\partial _{x}^{2}\eta _{2}|}{(v_{\epsilon }-1)^{\gamma +2}}|\partial _{x}^{3}V_{1}| \le \frac{C'}{\epsilon ^{11/2}}\Vert V_1\Vert _{\mathcal {X}}\Vert V_2\Vert _{\mathcal {X}}^2. \end{aligned}$$

To estimate the final integral appearing in (66), we cannot proceed as before and use $L^\infty _{t,x}$ norms, because only terms with higher order derivatives are present. We will thus use $L^2_tL^\infty _x$ norms instead, which can be estimated through the following lemma:

##### Lemma 3.10

For any $V\in \mathcal {X}$, let as before

$$\begin{aligned} \eta := \frac{\partial _x V}{v_\epsilon - 1}. \end{aligned}$$

Then we have the following bounds:

68 $$\begin{aligned}&\Vert \sqrt{\phi _{\epsilon }}\partial _{x}V\Vert _{L^{2}_{t}L^{\infty }_{x}} \le \frac{C'}{\sqrt{\epsilon }} \Vert V\Vert _{\mathcal {X}}, \end{aligned}$$

69 $$\begin{aligned}&\Vert \sqrt{\phi _{\epsilon }}\partial _{x}\eta \Vert _{L^{2}_{t}L^{\infty }_{x}} \le \frac{C'}{\epsilon ^{3/2}} \Vert V\Vert _{\mathcal {X}} \end{aligned}$$

##### Proof

As for Lemma 3.6, the proof relies on the interpolation equality (50). The proofs of (68) and (69) can be obtained in the same way, hence we only focus on (69). We first write that since

$$\begin{aligned} \partial _{x} ( \sqrt{\phi _{\epsilon }}\partial _{x}\eta ) = \frac{v_{\epsilon }'\phi _{\epsilon }'}{2\sqrt{\phi _{\epsilon }}} \partial _{x}\eta + \sqrt{\phi _{\epsilon }} \partial _{x}^{2}\eta , \end{aligned}$$

we have that

$$\begin{aligned} \Vert \partial _{x}(\sqrt{\phi _{\epsilon }}\partial _{x}\eta )\Vert _{L^{2}_{t,x}} \le \frac{C}{\epsilon } \Vert \sqrt{\phi _{\epsilon }} \partial _{x}\eta \Vert _{L^{2}_{t,x}} + \Vert \sqrt{\phi _{\epsilon }}\partial _{x}^{2} \eta \Vert _{L^{2}_{t,x}} \le \frac{C'}{\epsilon ^{2}} \Vert V\Vert _{\mathcal {X}}. \end{aligned}$$

Therefore,

$$\begin{aligned} \Vert \sqrt{\phi _{\epsilon }}\partial _{x}\eta \Vert _{L^{2}_{t}L^{\infty }_{x}} \le C\Vert \sqrt{\phi _{\epsilon }}\partial _{x}\eta \Vert _{L^{2}_{t,x}}^{1/2}\Vert \partial _{x}(\sqrt{\phi _{\epsilon }}\partial _{x}\eta )\Vert _{L^{2}_{t,x}}^{1/2} \le \frac{C'}{\epsilon ^{3/2}} \Vert V\Vert _{\mathcal {X}}. \end{aligned}$$

$$\square $$

Using (69) and (53), we have that

$$\begin{aligned} \begin{aligned} \epsilon \int _{0}^{t} \int _{\mathbb {R}} \frac{|\partial _{x}^{2}\eta _{2}|}{v_{\epsilon }-1} \frac{ |\partial _{x}^{2}V_{1}|^{2}}{(v_{\epsilon }-1)^{\gamma +2}}&\le C\int _{0}^{t} \int _{\mathbb {R}} \phi _{\epsilon }|\partial _{x}^{2}\eta _{2}| \frac{ |\partial _{x}^{2}V_{1}|^{2}}{(v_{\epsilon }-1)^{2}} \le C \int _{0}^{t} \int _{\mathbb {R}}\phi _{\epsilon }|\partial _{x}^{2}\eta _{2}| \left( |\partial _{x}\eta _{1}|^{2} + \frac{(v_{\epsilon }')^{2} |\partial _{x}V_{1}|^{2}}{(v_{\epsilon }-1)^{4}} \right) \\  &\le C\Vert \sqrt{\phi _{\epsilon }}\partial _{x}^{2}\eta _{2}\Vert _{L^{2}_{t,x}} \left( \Vert \partial _{x}\eta _{1}\Vert _{L^{\infty }_{t}L^{2}_{x}} \Vert \sqrt{\phi _{\epsilon }}\partial _{x}\eta _{1}\Vert _{L^{2}_{t}L^{\infty }_{x}} + \frac{C}{\epsilon ^{2}} \Vert \eta _{1}\Vert _{L^{\infty }_{t,x}}\Vert \sqrt{\phi _{\epsilon }}\partial _{x}V_{1}\Vert _{L^{2}_{t,x}} \right) \\  &\le \frac{C'}{\epsilon ^{11/2}} \Vert V_{1}\Vert _{\mathcal {X}}^{2}\Vert V_{2}\Vert _{\mathcal {X}}. \end{aligned} \end{aligned}$$

In summary, we find $J_{1} \le (C'/\epsilon ^{11/2})\Vert V_{2}\Vert _{\mathcal {X}} \Vert V_{1}\Vert _{\mathcal {X}}^{2}$.

For $J_2$, we proceed as in the $k=1$ case and obtain an estimate similar to (62):

$$\begin{aligned} |J_2| = \left| \int _{0}^{t}\int _{\mathbb {R}} \frac{\partial _{x}^{2}W_0}{v_{\epsilon }-1} \partial _{x}^{2}\eta _{2}\right| \le \frac{1}{6}\int _0^tD_2(\tau ;V_2)d\tau +C\left( \frac{t}{\epsilon }\right) ^{1/\gamma }\Vert \partial _x^2W_0\Vert _{L^2_x}^2+\frac{C}{\epsilon }\Vert \sqrt{x}\partial _x^2W_0\Vert _{L^2(\mathbb {R}_+)}^2. \end{aligned}$$

##### Remark 3.11

If $\sqrt{x}\partial _x^2W_0\notin L^2(\mathbb {R}_+)$, we still have using the Holder and Young inequalities that

$$\begin{aligned} |J_{2}| \le \frac{C}{\sqrt{\epsilon }} \Vert \partial _{x}^{2}W_0\Vert _{L^{2}_{t,x}} \Vert \sqrt{\phi _{\epsilon }}\partial _{x}^{2}\eta _{2}\Vert _{L^{2}_{t,x}} \le \frac{Ct}{\epsilon } \Vert \partial _{x}^{2}W_{0}\Vert _{L^{2}_{x}}^{2} + \frac{1}{6} \int _{0}^{t} D_{2}(\tau ;V_{2})~d\tau . \end{aligned}$$

We now use Equation (38) of Lemma 3.3 with $\alpha =1/6$ to obtain that

$$\begin{aligned} |J_{3}| = \left| \int _{0}^{t}\mathcal {L}_\epsilon (\partial _x\eta _2)\partial _x \eta _2\right| \le \frac{1}{6}\int _0^t\int _{\mathbb {R}}\phi _\epsilon (\partial _x^2\eta _2)^2+ \frac{C}{\epsilon ^2}\int _0^t\int _{\mathbb {R}}\phi _\epsilon (\partial _x \eta _2)^2. \end{aligned}$$

It now remains to estimate the term involving $\mathcal {C}_\epsilon$, i.e. $J_4$. We use this time Equation (39) of Lemma 3.3 with $\alpha = 1/6$ to get

$$\begin{aligned} |J_4| = \left| \int _{0}^{t}\int _{\mathbb {R}} \partial _{x}\eta _{2} ~ \mathcal {C}_\epsilon (\eta _{2})\right| \le \frac{1}{6} \int _0^t\int _{\mathbb {R}}\phi _\epsilon (\partial _x^2\eta )^2 + \frac{C}{\epsilon ^2} \int _0^t\int _{\mathbb {R}}\phi _\epsilon \left( (\partial _x\eta _2)^2+\frac{(\partial _x V_2^2)}{\epsilon ^2}\right) . \end{aligned}$$

Returning to (65) with our estimates for $K_{1}-K_{7}$ and $J_{1}-J_{3}$, we have

$$\begin{aligned} \begin{aligned} E_{2}(t; V_{2}) + \int _{0}^{t} D_{2}(\tau ;V_{2})~d\tau&\le E_{2}(0; V_{2}) + C\left( \frac{t}{\epsilon }\right) ^{1/\gamma } \Vert \partial _{x}^{2}W_{0}\Vert _{L^{2}_{x}}^{2} +\frac{C}{\epsilon }\Vert \sqrt{x}\partial _x^2 W_0\Vert _{L^2(\mathbb {R}_+)}^2+ \frac{C'}{\epsilon ^{11/2}} \Vert V_{1}\Vert _{\mathcal {X}}^{2}\Vert V_{2}\Vert _{\mathcal {X}} \\  &~~~ + \frac{C}{\epsilon ^{2}} \int _{0}^{t} D_{1}(\tau ;V_{2})~d\tau + \frac{C}{\epsilon ^{4}} \int _{0}^{t}D_{0}(\tau ;V_{2})~d\tau . \end{aligned} \end{aligned}$$

We may assume that each $C>0$ appearing on the right hand-side is bounded by $B_0 = B_0(\gamma , v_{+},s) > 0$. Thus letting $k_0 := 1/ (2B_0)$ and multiplying by $k_0\epsilon ^{4}$ results in

70 $$\begin{aligned} \begin{aligned} k_0 \epsilon ^{4} E_{2}(t; V_{2}) + k_0\epsilon ^{4} \int _{0}^{t} D_{2}(\tau ;V_{2})~d\tau&\le k_0 \epsilon ^{4} E_{2}(0; V_{2}) + \left( \frac{t}{\epsilon }\right) ^{1/\gamma }\epsilon ^{4} \Vert \partial _{x}^{2}W_{0}\Vert _{L^{2}_{x}}^{2} + \frac{C'}{\epsilon ^{3/2}}\Vert V_{1}\Vert _{\mathcal {X}}^{2} \Vert V_{2}\Vert _{\mathcal {X}} \\  &~~~+ \frac{\epsilon ^{2}}{2} \int _{0}^{t} D_{1}(\tau ;V_{2})~d\tau + \frac{1}{2} \int _{0}^{t}D_{0}(\tau ;V_{2})~d\tau + \epsilon ^3\Vert \sqrt{x}\partial _x^2 W_0\Vert _{L^2(\mathbb {R}_+)}^2. \end{aligned} \end{aligned}$$

Adding to what we found for $k=1$, i.e. (64), we find after simplifying the terms containing $D_1$ that

$$\begin{aligned} \begin{aligned}&\epsilon ^{2} E_{1}(t; V_{2}) + \epsilon ^{2} \int _{0}^{t} D_{1}(\tau ;V_{2})~d\tau + k_0 \epsilon ^{4} E_{2}(t; V_{2}) + k_0 \epsilon ^{4} \int _{0}^{t} D_{2}(\tau ;V_{2})~d\tau \\&\quad \le \epsilon ^{2} E_{1}(0; V_{2}) + k_0\epsilon ^{4} E_{2}(0; V_{2})+ \left( \frac{t}{\epsilon }\right) ^{1/\gamma }\left( C\epsilon ^2\Vert \partial _x W_0\Vert ^2_{L^2_x}+\epsilon ^{4} \Vert \partial _{x}^{2}W_{0}\Vert _{L^{2}_{x}}^{2}\right) + \frac{C'}{\epsilon ^{3/2}}\Vert V_{1}\Vert _{\mathcal {X}}^{2} \Vert V_{2}\Vert _{\mathcal {X}} \\&\qquad + C\epsilon \Vert \sqrt{x}\partial _x W_0\Vert ^2_{L^2(\mathbb {R}_+)}+ \epsilon ^3\Vert \sqrt{x}\partial _x^2 W_0\Vert ^2_{L^2(\mathbb {R}_+)} + \left( C+\frac{1}{2}\right) \int _0^tD_0(\tau ;V_2)~d\tau . \end{aligned} \end{aligned}$$

Again, we may assume that each $C+1/2, C>0$ that appear on the right-hand side is bounded by $B_1=B_1(\gamma ,v_+,s)>0$. Without loss of generality, we may assume that $B_1>1$. Thus letting $k_1:=1/(2B_1)$ and multiplying by $k_1$ yields

$$\begin{aligned} \begin{aligned}&k_1 \epsilon ^{2} E_{1}(t; V_{2}) + k_1\epsilon ^{2} \int _{0}^{t} D_{1}(\tau ;V_{2})~d\tau + k_1 k_0 \epsilon ^{4} E_{2}(t; V_{2}) + k_1k_0\epsilon ^{4} \int _{0}^{t} D_{2}(\tau ;V_{2})~d\tau \\&\quad \le k_1\epsilon ^{2} E_{1}(0; V_{2}) + k_1 k_0 \epsilon ^{4} E_{2}(0; V_{2})+ \left( \frac{t}{\epsilon }\right) ^{1/\gamma }\left( \epsilon ^2\Vert \partial _x W_0\Vert ^2_{L^2_x}+\epsilon ^{4} \Vert \partial _{x}^{2}W_{0}\Vert _{L^{2}_{x}}^{2}\right) + \frac{C'}{\epsilon ^{3/2}}\Vert V_{1}\Vert _{\mathcal {X}}^{2} \Vert V_{2}\Vert _{\mathcal {X}} \\&\qquad + \epsilon \Vert \sqrt{x}\partial _x W_0\Vert ^2_{L^2(\mathbb {R}_+)}+ \epsilon ^3\Vert \sqrt{x}\partial _x^2 W_0\Vert ^2_{L^2(\mathbb {R}_+)} + \frac{1}{2} \int _0^tD_0(\tau ;V_2)~d\tau . \end{aligned} \end{aligned}$$

Adding this inequality to the one that we obtained for $k=0$, i.e. (57), and simplifying the terms depending on $D_0$, we get

$$\begin{aligned} \begin{aligned}&E_{0}(t; V_{2}) + \int _{0}^{t} D_{0}(\tau ;V_{2})~d\tau + k_1 \epsilon ^{2} \left( E_{1}(t; V_{2}) +\int _{0}^{t} D_{1}(\tau ;V_{2})~d\tau \right) + k_1 k_0 \epsilon ^{4}\left( E_{2}(t; V_{2}) + \int _{0}^{t} D_{2}(\tau ;V_{2})~d\tau \right) \\&\quad \le E_{0}(0; V_{2}) + k_1\epsilon ^{2} E_{1}(0; V_{2}) + k_1 k_0 \epsilon ^{4} E_{2}(0; V_{2})+ \left( \frac{t}{\epsilon }\right) ^{1/\gamma }\left( \epsilon ^2\Vert \partial _x W_0\Vert ^2_{L^2_x}+\epsilon ^{4} \Vert \partial _{x}^{2}W_{0}\Vert _{L^{2}_{x}}^{2}\right) + \frac{C'}{\epsilon ^{3/2}}\Vert V_{1}\Vert _{\mathcal {X}}^{2} \Vert V_{2}\Vert _{\mathcal {X}} \\&\qquad + \epsilon \Vert \sqrt{x}\partial _x W_0\Vert ^2_{L^2(\mathbb {R}_+)}+ \epsilon ^4\Vert \sqrt{x}\partial _x^2 W_0\Vert ^2_{L^2(\mathbb {R}_+)}+ C\Vert W_0\Vert _{L^2_x}^2 + \frac{C}{\epsilon }\Vert \sqrt{x}W_0\Vert ^2_{L^2(\mathbb {R}_+)}. \end{aligned} \end{aligned}$$

Again, we may assume that each $C>0$ in the right-hand side is bounded by $B_2=B_2(s,\gamma ,v_+)>1$. We set $k_2:=1/B_2$ and multiply by $k_2$. In order to simplify computations, we also define

71 $$\begin{aligned} c_0:= k_2,\qquad c_1:= k_2 k_1, \qquad c_2:= k_2 k_1 k_0. \end{aligned}$$

We then obtain

$$\begin{aligned} \begin{aligned} \sum _{k=0}^2 c_k \epsilon ^{2k}\left( E_{k}(t; V_{2}) + \int _{0}^{t} D_{k}(\tau ;V_{2})~d\tau \right) \le&\sum _{k=0}^2 \left( c_k \epsilon ^{2k} E_{k}(0; V_{2})+\epsilon ^{2k-1}\Vert \sqrt{x}\partial _x^k W_0\Vert _{L^2(\mathbb {R}_+)}^2\right) + \Vert W_0\Vert _{L^2_x}^2\\&+ \left( \frac{t}{\epsilon }\right) ^{1/\gamma }\left( \epsilon ^2\Vert \partial _x W_0\Vert ^2_{L^2_x}+\epsilon ^{4} \Vert \partial _{x}^{2}W_{0}\Vert _{L^{2}_{x}}^{2}\right) + \frac{C'}{\epsilon ^{3/2}}\Vert V_{1}\Vert _{\mathcal {X}}^{2} \Vert V_{2}\Vert _{\mathcal {X}}. \end{aligned} \end{aligned}$$

We use the inequality $t^{1/\gamma }\le T^{1/\gamma }$ in the right-hand side, and then take the supremum in time in the left-hand side to obtain

72 $$\begin{aligned} \begin{aligned} \sup _{t \in [0,T]}&\left( \sum _{k=0}^{2} c_{k}\epsilon ^{2k} \left[ E_{k}(t;V_{2}) + \int _{0}^{t}D_{k}(\tau ;V_{2})~d\tau \right] \right) \le \sum _{k=0}^2\left[ c_k \epsilon ^{2k} E_k(0,V_2)+\epsilon ^{2k-1}\Vert \sqrt{x}\partial _x^k W_0\Vert _{L^2(\mathbb {R}_+)}^2\right] \\  &+ \frac{C'}{\epsilon ^{3/2}} \Vert V_{1}\Vert _{\mathcal {X}}^{2}\Vert V_{2}\Vert _{\mathcal {X}} + \Vert W_{0}\Vert _{L^{2}_{x}}^{2} + \epsilon ^2\left( \frac{T}{\epsilon }\right) ^{1/\gamma }\left( \left\| \partial _{x}W_{0}\right\| _{L^{2}_{t,x}}^{2}+\epsilon ^2\Vert \partial _x^2W_0\Vert _{L^2_x}^2\right) . \end{aligned} \end{aligned}$$

Note that, when $W_0=0$, one can take $T=+\infty$ and obtain a global bound. Using the constants $c_0, c_1, c_2$ in the definition of the norm $\Vert \cdot \Vert _{\mathcal {X}}$, (72) can be recast as

$$\begin{aligned} \begin{aligned} \Vert V_{2}\Vert _{\mathcal {X}}^{2} \le&\sum _{k=0}^2\left[ c_k \epsilon ^{2k} E_k(0,V_2)+\epsilon ^{2k-1}\Vert \sqrt{x}\partial _x^k W_0\Vert _{L^2(\mathbb {R}_+)}^2\right] + \Vert W_{0}\Vert _{L^{2}_{x}}^{2} \\&+ \epsilon ^2\left( \frac{T}{\epsilon }\right) ^{1/\gamma }\left( \left\| \partial _{x}W_{0}\right\| _{L^{2}_{t,x}}^{2}+\epsilon ^2\Vert \partial _x^2W_0\Vert _{L^2_x}^2\right) +\frac{C'}{\epsilon ^{3/2}}\Vert V_1\Vert _{\mathcal {X}}^2\Vert V_2\Vert _{\mathcal {X}} \\ \le&C' \delta ^{2} \epsilon ^{3/2} \Vert V_{2}\Vert _{\mathcal {X}}+ \delta _{0}^{2}\epsilon ^{3} , \end{aligned} \end{aligned}$$

where we have used our assumption on the initial data (47). Taking $\delta < 1$ to be such that $\delta < 1/(\sqrt{2}C')$, defining $\delta _{0} := \delta /2$ and using Young’s inequality gives us

73 $$\begin{aligned} \Vert V_{2}\Vert _{\mathcal {X}}^{2} \le \delta ^{2} \epsilon ^{3}, \end{aligned}$$

and so $V_{2} \in B_{\delta }$ as required. This completes the first part of Proposition 3.5.

##### Remark 3.12

When we do not suppose that $\sqrt{x}\partial _x^k W_0\in L^2(\mathbb {R}_+)$ for $k=0,1,2$, we still obtain that

$$\begin{aligned} \begin{aligned} \Vert V_2\Vert _{\mathcal {X}}^2 \le \sum _{k=0}^2\left[ c_k\epsilon ^{2k}E_k(0;V_2)+T\epsilon ^{2k-1}\Vert \partial _x^k W_0\Vert _{L^2_x}^2\right] + C'\delta ^2 \epsilon ^{3/2} \Vert V_2\Vert _{\mathcal {X}}. \end{aligned} \end{aligned}$$

Hence the proof of the proposition still works after suitable modification of the assumption on the initial data. In this case, one has to impose the condition

$$\begin{aligned} \sum _{k=0}^2\left[ c_k\epsilon ^{2k}E_k(0;V_2)+T\epsilon ^{2k-1}\Vert \partial _x^k W_0\Vert _{L^2_x}^2\right] \le \delta _0^2\epsilon ^3. \end{aligned}$$

##### Remark 3.13

If the integrability assumption on the perturbations is removed, one can still perform computations analogous to the ones of Sects. 3.4.2,3.4.3, in order to obtain energy estimates for the variable $\eta$. Recall that $\eta$ corresponds to the original perturbation, divided by $(v_\epsilon -1)$ (see also Remark 3.1 for the importance of the variable $\eta$). However, these estimates are only valid on a local time interval. Indeed, one can check that equations (36) and (37) coupled to the bounds (40) and (41) formally yield the following estimate:

74 $$\begin{aligned} \frac{d}{dt}\beta (t) + \left[ 1-\frac{C}{\epsilon ^{1/2}}\beta (t)^{1/2}\right] \int _{\mathbb {R}}\phi _\epsilon \left[ |\partial _x \eta |^2+\epsilon ^2|\partial ^2_x\eta |^2\right] \le \frac{C}{\epsilon }\beta (t) + \frac{1}{\epsilon }\Vert \tilde{w}_0\Vert ^2, \end{aligned}$$

where

75 $$\begin{aligned} \beta (t):=\int _{\mathbb {R}}\left[ \eta ^2+\epsilon ^2|\partial _x\eta |^2\right] , \qquad \Vert \tilde{w}_0\Vert ^2:= \int _\mathbb {R}\left[ |w-w_\epsilon |^2+\epsilon ^2|\partial _x(w-w_\epsilon )|^2\right] , \end{aligned}$$

and $C=C(s,\gamma ,v_+)$. As long as the constraint $\beta (t)< \epsilon / C^2$ holds, one can apply Gronwall’s lemma and obtain that

76 $$\begin{aligned} \beta (t)\le \left( \beta (0) + \frac{1}{C}\Vert \tilde{w}_0\Vert ^2\right) \exp \left( \frac{Ct}{\epsilon }\right) - \frac{1}{C}\Vert \tilde{w}_0\Vert ^2. \end{aligned}$$

This last inequality coupled to the constraint $\beta (t)< \epsilon / C^2$ yields an existence time *T* with a lower bound (note that $\Vert \tilde{w}_0\Vert$ is time-independent):

77 $$\begin{aligned} T\ge \frac{\epsilon }{C}\log \left( \frac{C\Vert \tilde{w}_0\Vert ^2+\epsilon }{C\Vert \tilde{w}_0\Vert ^2+C^2\beta (0)}\right) . \end{aligned}$$

### The Map $\mathcal {A}^{\epsilon }$ is a Contraction

In this subsection we aim to show that the map $\mathcal {A}^{\epsilon }$ is a contraction. We consider two elements $V_{1}, V_{1}' \in B_{\delta }$ and define $V_{2} := \mathcal {A}^{\epsilon }(V_{1})$ and $V_{2}' := \mathcal {A}^{\epsilon }(V_{2}')$. Our goal is to show that there exists $\delta < 1$ with $\Vert V_{2} - V_{2}'\Vert _{\mathcal {X}} \le \delta \Vert V_{1} - V_{1}'\Vert _{\mathcal {X}}$. Defining $\tilde{V}_{1} := V_{1} - V_{1}'$ and $\tilde{V}_{2} := V_{2} - V_{1}'$, the following equations are satisfied:

78 $$\begin{aligned} \left\{ \begin{aligned}&\partial _t \tilde{V}_{2} - \partial _{x}(\phi _{\epsilon }(v_{\epsilon }) \partial _{x}\tilde{V}_{2}) = \partial _{x} \left( H(\partial _{x}V_{1}) - H(\partial _{x}V_{1}') \right) ,\\&\partial _t \tilde{\eta }_{2} -\partial _x (\phi _{\epsilon }(v_{\epsilon }) \partial _{x}\tilde{\eta }_{2}) = \frac{\partial _{x}^{2}\left( H(\partial _{x}V_{1}) - H(\partial _{x}V_{1}') \right) }{v_{\epsilon }-1} + \mathcal {L}_{\epsilon }(\tilde{\eta }_{2}), \\&\partial _{t} \partial _{x}\tilde{\eta }_{2} - \partial _{x}(\phi _{\epsilon }(v_{\epsilon })\partial _{x}^{2}\tilde{\eta }_{2}) = \partial _{x}\left( \frac{\partial _{x}^{2}\left( H(\partial _{x}V_{1}) - H(\partial _{x}V_{1}') \right) }{v_{\epsilon }-1} \right) + \mathcal {L}_{\epsilon }(\partial _{x}\tilde{\eta }_{2}) + \mathcal {C}_\epsilon (\tilde{\eta }_{2}). \end{aligned} \right. \end{aligned}$$

We see that $\tilde{V}_2$, $\tilde{\eta _2}$ and $\partial _x\tilde{\eta }_2$ solve the same equations than $V_2,\eta _2,\partial _x\eta _2$ respectively, up to two differences:

- The constant term $W_0$ is equal to zero here, as well as the initial data,
- The nonlinear terms (depending on *H*) are different.

With this in mind, multiplying the equations by $\tilde{V}_{2}, \tilde{\eta }_{2}, \partial _{x}\tilde{\eta }_{2}$ respectively and using again Lemma 3.3 (taking advantage of the linearity of $\mathcal {L}_\epsilon$ and $\mathcal {C}_\epsilon$), we see that the computations of the previous section can be repeated. We get a similar estimate after suitable modification of the nonlinear terms. Since here $W_0$ and the initial data are equal to zero, we are left with

79 $$\begin{aligned} \begin{aligned} \Vert \tilde{V}_{2}\Vert _{\mathcal {X}}^{2} \le&C\left| \int _{0}^{t} \int _{\mathbb {R}} \tilde{V}_{2} ~\partial _{x} \left( H(\partial _{x}V_{1}) - H(\partial _{x}V_{1}') \right) \right| + C\epsilon ^{2}\left| \int _{0}^{t} \int _{\mathbb {R}} \tilde{\eta }_{2}~ \left( \frac{\partial _{x}^{2}\left( H(\partial _{x}V_{1}) - H(\partial _{x}V_{1}') \right) }{v_{\epsilon }-1}\right) \right| \\  &+ C\epsilon ^{4}\left| \int _{0}^{t}\int _{\mathbb {R}} \partial _{x} \tilde{\eta }_{2} ~\partial _{x} \left( \frac{\partial _{x}^{2}(H(\partial _{x}V_{1}) - H(\partial _{x}V_{1}')}{v_{\epsilon }-1} \right) \right| =: C\sum _{k=1}^{3}\epsilon ^{2k-2}|L_{k}|. \end{aligned} \end{aligned}$$

To estimate each $L_{k}$ we will need the following result.

#### Lemma 3.14

If $f_{1}, f_{2} \in \mathcal {X}$ are such that $\Vert \frac{f_{1}}{v_\epsilon -1}\Vert _\infty + \Vert \frac{f_{2}}{v_\epsilon -1}\Vert _\infty \le \delta$ for some $\delta < 1$, then there exists a constant $C>0$ independent of $\epsilon$ such that

80 $$\begin{aligned} |H(f_{1}) - H(f_{2})|&\le \frac{C \phi _{\epsilon }(v_{\epsilon })}{v_{\epsilon }-1} |f_{1}-f_{2}|( |f_{1}| + |f_{2}|), \end{aligned}$$

81 $$\begin{aligned} |\partial _{x}H(f_{1}) - \partial _{x}H(f_{2})|&\le \frac{C\phi _{\epsilon }(v_{\epsilon })}{v_{\epsilon }-1} \left( \frac{v_{\epsilon }'}{v_{\epsilon }-1} |f_{1}-f_{2}| (|f_{1}|+|f_{2}|) + |\partial _{x}(f_{1}-f_{2})| |f_{1}| + |\partial _{x}f_{2}| |f_{1}-f_{2}| \right) , \end{aligned}$$

82 $$\begin{aligned} |\partial _{x}^{2}H(f_{1}) - \partial _{x}^{2}H(f_{2})|&\le \frac{C(v_{\epsilon }-1)^{\gamma -2}}{\epsilon } |f_{1}-f_{2}|(|f_{1}|+f_{2}|) \nonumber \\  &\left. \quad + \frac{C}{(v_{\epsilon }-1)^{2}}\left( |\partial _{x}(f_{1}-f_{2})||f_{2}| + |f_{1}-f_{2}||\partial _{x}f_{1}|\right) \right. \nonumber \\  &\left. \quad + \frac{C\phi _{\epsilon }(v_{\epsilon })}{v_{\epsilon }-1}|\partial _{x}(f_{1}-f_{2})|(|\partial _{x}f_{1}|+ |\partial _{x}f_{2}|) \right. \nonumber \\  &\quad + \frac{C\phi _{\epsilon }(v_{\epsilon })}{v_{\epsilon }-1}\left( |f_{1}-f_{2}| |\partial _{x}^{2}f_{1}| + |\partial _{x}^{2}(f_{1}-f_{2})||f_{2}| + \frac{(\partial _{x}f_{2})^{2}}{v_{\epsilon }-1}|f_{1}-f_{2}| \right) . \end{aligned}$$

The proof follows the same approach as that of Lemma 2.4, and a very similar result can be seen in Lemma 3.4 of [12]. Thus we omit the proof. Integrating by parts and using (80),

$$\begin{aligned} \begin{aligned} |L_{1}|&= \left| \int _{0}^{t} \int _{\mathbb {R}} \partial _{x}\tilde{V}_{2}~( H(\partial _{x}V_{1}) - H(\partial _{x} V_{1}'))\right| \le \int _{0}^{t} \int _{\mathbb {R}} |\partial _{x}\tilde{V}_{2}| \left( \frac{\phi _{\epsilon }}{v_{\epsilon }-1} |\partial _{x}\tilde{V}_{1}| (|\partial _{x}V_{1}| + |\partial _{x}V_{1}'|) \right) \\  &\le C \int _{0}^{t} \int _{\mathbb {R}} \sqrt{\phi _{\epsilon }} |\partial _{x}\tilde{V}_{2}| \frac{|\partial _{x}\tilde{V}_{1}|}{v_{\epsilon }-1} \left( \sqrt{\phi _{\epsilon }}|\partial _{x}V_{1}|+\sqrt{\phi _{\epsilon }}|\partial _{x}V_{1}'|\right) \\  &\le \left\| \tilde{\eta }_{1} \right\| _{L^{\infty }_{t,x}} \Vert \tilde{V}_{2}\Vert _{\mathcal {X}} (\Vert V_{1}\Vert _{\mathcal {X}} + \Vert V_{2}\Vert _{\mathcal {X}}). \end{aligned} \end{aligned}$$

Using the definition of the norm,

83 $$\begin{aligned} |L_{1}| \le \frac{C}{\epsilon ^{3/2}}\Vert \tilde{V}_{2}\Vert _{\mathcal {X}} \Vert \tilde{V}_{1}\Vert _{\mathcal {X}} \left( \Vert V_{1}\Vert _{\mathcal {X}} + \Vert V_{1}'\Vert _{\mathcal {X}}\right) . \end{aligned}$$

To deal with $L_{2}$, we first integrate by parts to get

84 $$\begin{aligned} \begin{aligned} L_{2}&= - \int _{0}^{t} \int _{\mathbb {R}} \tilde{\eta }_{2} \frac{v_{\epsilon }' (\partial _{x}H(\partial _{x}V_{1}) - \partial _{x}H(\partial _{x}V_{1}'))}{(v_{\epsilon }-1)^{2}} - \int _{0}^{t} \int _{\mathbb {R}} \frac{\partial _{x}\tilde{\eta }_{2}}{v_{\epsilon }-1} (\partial _{x}H(\partial _{x}V_{1}) - \partial _{x}H(\partial _{x}V_{1}')) \\  &=: (2a) + (2b). \end{aligned} \end{aligned}$$

Using (81),

$$\begin{aligned} \begin{aligned} |(2a)|&\le C\int _{0}^{t} \int _{\mathbb {R}} |\tilde{\eta }_{2}| \frac{(v_{\epsilon }')^{2}\phi _{\epsilon }}{(v_{\epsilon }-1)^{4}} |\partial _{x} \tilde{V}_{1}| ( |\partial _{x}V_{1}| + |\partial _{x}V_{1}'|) + C\int _{0}^{t} \int _{\mathbb {R}} |\tilde{\eta }_{2}| \frac{v_{\epsilon }'\phi _{\epsilon }}{(v_{\epsilon }-1)^{3}} |\partial _{x}^{2} \tilde{V}_{1}| |\partial _{x}V_{1}| \\  &~~~+C \int _{0}^{t} \int _{\mathbb {R}} |\tilde{\eta }_{2}| \frac{v_{\epsilon }'\phi _{\epsilon }}{(v_{\epsilon }-1)^{3}} |\partial _{x}^{2}V_{1}'| |\partial _{x} \tilde{V}_{1}| \\  &\le C\left\| \frac{(v_\epsilon ')^2}{(v_\epsilon -1)^4}\right\| _{L^\infty _{t,x}} \Vert \tilde{\eta }_{2}\Vert _{L^{\infty }_{t,x}} \Vert \sqrt{\phi _{\epsilon }} \partial _{x}\tilde{V}_{1}\Vert _{L^{2}_{t,x}} ( \Vert \sqrt{\phi _{\epsilon }} \partial _{x}V_{1}\Vert _{L^{2}_{t,x}} + \Vert \sqrt{\phi _{\epsilon }} \partial _{x}V_{1}'\Vert _{L^{2}_{t,x}}) \\  &~~~+ C\left\| \frac{v_\epsilon '}{(v_\epsilon -1)^2}\right\| _{L^\infty _{t,x}}\Vert \tilde{\eta }_{2}\Vert _{L^{\infty }_{t,x}} \left( \Vert \frac{\sqrt{\phi _{\epsilon }} \partial _{x}^{2}\tilde{V}_{1}}{v_{\epsilon }-1}\Vert _{L^{2}_{t,x}} \Vert \sqrt{\phi _{\epsilon }} \partial _{x}V_{1}\Vert _{L^{2}_{t,x}} + \Vert \frac{\sqrt{\phi _{\epsilon }} \partial _{x}^{2}V_{1}'}{v_{\epsilon }-1}\Vert _{L^{2}_{t,x}} \Vert \sqrt{\phi _{\epsilon }} \partial _{x}\tilde{V}_{1}\Vert _{L^{2}_{t,x}}\right) . \end{aligned} \end{aligned}$$

Using (50) and (60), we therefore find

$$\begin{aligned} \begin{aligned} |(2a)|&\le \frac{C}{\epsilon ^{7/2}} \Vert \tilde{V}_{2}\Vert _{\mathcal {X}} \Vert \tilde{V}_{1}\Vert _{\mathcal {X}} \left( \Vert V_{1}\Vert _{\mathcal {X}} + \Vert V_{1}'\Vert _{\mathcal {X}}\right) . \end{aligned} \end{aligned}$$

For (2*b*) we use (81) to get

$$\begin{aligned} \begin{aligned} |(2b)|&\le \int _{0}^{t} \int _{\mathbb {R}} |\partial _{x} \tilde{\eta }_{2}| \frac{ \phi _{\epsilon }}{(v_{\epsilon }-1)^2} \left( \frac{v_{\epsilon }'}{v_{\epsilon }-1} |\partial _{x}\tilde{V}_{1}|( |\partial _{x}V_{1}| + |\partial _{x}V_{1}'|) + |\partial _{x}^{2}\tilde{V}_{1}| |\partial _{x}V_{1}| + |\partial _{x}^{2}V_{1}'||\partial _{x}\tilde{V}_{1}| \right) \\  &=: b_{1} + b_{2} + b_{3}. \end{aligned} \end{aligned}$$

We then have that

$$\begin{aligned} \begin{aligned} b_{1}&\le C\left\| \frac{v_\epsilon '}{(v_\epsilon -1)^2} \right\| _{L^\infty _{t,x}}\int _{0}^{t} \int _{\mathbb {R}} |\sqrt{\phi _{\epsilon }}\partial _{x} \tilde{\eta }_{2}| |\sqrt{\phi _{\epsilon }}\partial _{x}\tilde{V}_{1}|( |\eta _1| + |\eta _1'|) \\&\le \frac{C}{\epsilon } \Vert \sqrt{\phi _{\epsilon }}\partial _{x}\tilde{\eta }_{2}\Vert _{L^{2}_{t,x}}\Vert \sqrt{\phi _{\epsilon }}\partial _{x}\tilde{V}_{1}\Vert _{L^{2}_{t,x}} \left( \Vert \eta _{1}\Vert _{L^{\infty }_{t,x}} + \Vert \eta _{1}'\Vert _{L^{\infty }_{t,x}}\right) \\&\le \frac{C}{\epsilon ^{7/2}} \Vert \tilde{V}_{1}\Vert _{\mathcal {X}}\Vert \tilde{V}_{2}\Vert _{\mathcal {X}} \left( \Vert V_{1}\Vert _{\mathcal {X}} + \Vert V_{1}'\Vert _{\mathcal {X}}\right) . \end{aligned} \end{aligned}$$

Next,

$$\begin{aligned} \begin{aligned} b_{2}&\le \int _{0}^{t} \int _{\mathbb {R}} |\sqrt{\phi _{\epsilon }}\partial _{x}\tilde{\eta }_{2}| |\sqrt{\phi _{\epsilon }} \frac{\partial _{x}^{2}\tilde{V}_{1}}{v_{\epsilon }-1} | |\eta _1| \le C \Vert \eta _{1}\Vert _{L^{\infty }_{t,x}}\Vert \sqrt{\phi _{\epsilon }}\partial _{x}\tilde{\eta }_{2}\Vert _{L^{2}_{t,x}} \Vert \sqrt{\phi _{\epsilon }}\frac{\partial _{x}^{2}\tilde{V}_{1}}{v_{\epsilon }-1}\Vert _{L^{2}_{t,x}} \\  &\le \frac{C}{\epsilon ^{7/2}} \Vert \tilde{V}_{1}\Vert _{\mathcal {X}}\Vert \tilde{V}_{2}\Vert _{\mathcal {X}}\Vert V_{1}\Vert _{\mathcal {X}}. \end{aligned} \end{aligned}$$

We find similarly that

85 $$\begin{aligned} b_{3} \le \frac{C}{\epsilon ^{7/2}}\Vert \tilde{V}_{1}\Vert _{\mathcal {X}}\Vert \tilde{V}_{2}\Vert _{\mathcal {X}}\Vert V_{1}'\Vert _{\mathcal {X}}, \end{aligned}$$

which yields with the previous estimates that

$$\begin{aligned} \epsilon ^2 |L_2| \le \epsilon ^2 \frac{C}{\epsilon ^{7/2}} \Vert \tilde{V}_{2}\Vert _{\mathcal {X}} \Vert \tilde{V}_{1}\Vert _{\mathcal {X}} \left( \Vert V_{1}\Vert _{\mathcal {X}} + \Vert V_{1}'\Vert _{\mathcal {X}}\right) = \frac{C}{\epsilon ^{3/2}} \Vert \tilde{V}_{2}\Vert _{\mathcal {X}} \Vert \tilde{V}_{1}\Vert _{\mathcal {X}} \left( \Vert V_{1}\Vert _{\mathcal {X}} + \Vert V_{1}'\Vert _{\mathcal {X}}\right) . \end{aligned}$$

We now estimate the term $L_{3}$. Using (82) and (48),

86 $$\begin{aligned} \begin{aligned} |L_{3}| \le&\frac{C}{\epsilon }\int _{0}^{t} \int _{\mathbb {R}} |\partial _{x}^{2} \tilde{\eta }_{2}| (v_\epsilon -1)^{\gamma -3} |\partial _{x}\tilde{V}_{1}|(|\partial _{x}V_{1}| + |\partial _{x}V_{1}'|) + C\int _{0}^{t} \int _{\mathbb {R}}\frac{|\partial _{x}^{2} \tilde{\eta }_{2} |}{(v_{\epsilon }-1)^{3}}(|\partial _{x}^{2}\tilde{V}_{1}||\partial _{x}V_{1}| + |\partial _{x}\tilde{V}_{1}||\partial _{x}^{2}V_{1}|) \\  &+ C\int _{0}^{t}\int _{\mathbb {R}} |\partial _{x}^{2} \tilde{\eta }_{2}| \frac{\phi _{\epsilon }}{(v_{\epsilon }-1)^2} |\partial _{x}^{2}\tilde{V}_{1}|(|\partial _{x}^{2}V_{1}| + |\partial _{x}^{2}V_{1}'|) \\  &+ C\int _{0}^{t}\int _{\mathbb {R}} |\partial _{x}^{2} \tilde{\eta }_{2}| \frac{\phi _{\epsilon }}{(v_{\epsilon }-1)^2}(|\partial _{x}\tilde{V}_{1}||\partial _{x}^{3}V_{1}| + |\partial _{x}^{3}\tilde{V}_{1}||\partial _{x}V_{1}'|) \\  &+ C\int _{0}^{t}\int _{\mathbb {R}} |\partial _{x}^{2} \tilde{\eta }_{2} |\frac{\phi _{\epsilon }}{(v_{\epsilon }-1)^{3}} |\partial _{x}^{2}V_{1}'|^{2}|\partial _{x}\tilde{V}_{1}| =: \sum _{n=1}^{5}M_{n}. \end{aligned} \end{aligned}$$

Using (60),

$$\begin{aligned} \begin{aligned} M_{1}&\le \frac{C}{\epsilon }\left\| \frac{(v_\epsilon -1)^{\gamma -2}}{\phi _\epsilon }\right\| _{L^\infty _{t,x}} \Vert \sqrt{\phi _{\epsilon }} \partial _{x}^{2} \tilde{\eta }_{2}\Vert _{L^{2}_{t,x}} \Vert \sqrt{\phi _{\epsilon }} \partial _{x}\tilde{V}_{1}\Vert _{L^{2}_{t,x}} \left( \Vert \eta _{1}\Vert _{L^{\infty }_{t,x}} + \Vert \eta _{1}'\Vert _{L^{\infty }_{t,x}} \right) \\  &\le \frac{C}{\epsilon ^{11/2}} \Vert \tilde{V}_{2}\Vert _{\mathcal {X}} \Vert \tilde{V}_{1}\Vert _{\mathcal {X}} (\Vert V_{1}\Vert _{\mathcal {X}} + \Vert V_{1}'\Vert _{\mathcal {X}} ). \end{aligned} \end{aligned}$$

Similarly,

$$\begin{aligned} \begin{aligned} M_{2}&\le C\left\| \frac{1}{\phi _\epsilon (v_\epsilon -1)}\right\| _{L^\infty _{t,x}}\Vert \sqrt{\phi _{\epsilon }}\partial _{x}^{2}\tilde{\eta }_{2}\Vert _{L^{2}_{t,x}} \left( \Vert \sqrt{\phi _{\epsilon }}\frac{\partial _{x}^{2}\tilde{V}_{1}}{v_{\epsilon }-1} \Vert _{L^{2}_{t,x}} \Vert \eta _{1}\Vert _{L^{\infty }_{t,x}} + \Vert \tilde{\eta }_{1}\Vert _{L^{\infty }_{t,x}}\Vert \sqrt{\phi _{\epsilon }}\frac{\partial _{x}^{2}V_{1}}{v_{\epsilon }-1} \Vert _{L^{2}_{t,x}} \right) \\  &\le \frac{C}{\epsilon ^{11/2}} \Vert \tilde{V}_{1}\Vert _{\mathcal {X}} \Vert \tilde{V}_{2}\Vert _{\mathcal {X}}(\Vert V_{1}\Vert _{\mathcal {X}}+\Vert V_{1}'\Vert _{\mathcal {X}}). \end{aligned} \end{aligned}$$

Next,

$$\begin{aligned} \begin{aligned} M_{3}&\le C\int _{0}^{t} \int _{\mathbb {R}} |\sqrt{\phi _{\epsilon }}\partial _{x}^{2}\tilde{\eta }_{2}| \left| \sqrt{\phi _{\epsilon }} \frac{\partial _{x}^{2}\tilde{V}_{1}}{v_{\epsilon }-1} \right| \left( \left| \frac{\partial _{x}^{2}V_{1}}{v_\epsilon -1}\right| + \left| \frac{\partial _{x}^{2}V_{1}'}{v_\epsilon -1}\right| \right) \\&\le C \left\| \sqrt{\phi _{\epsilon }}\partial _{x}^{2}\tilde{\eta }_{2}\right\| _{L^{2}_{t,x}} \left\| \sqrt{\phi _{\epsilon }} \frac{\partial _{x}^{2}\tilde{V_{1}}}{v_{\epsilon }-1}\right\| _{L^{2}_{t}L^{\infty }_{x}}\left( \left\| \frac{\partial _{x}^{2}V_{1}}{v_\epsilon -1}\right\| _{L^{\infty }_{t}L^{2}_{x}} + \left\| \frac{\partial _{x}^{2}V_{1}'}{v_\epsilon -1}\right\| _{L^{\infty }_{t}L^{2}_{x}} \right) . \end{aligned} \end{aligned}$$

Using (53), (69) and (68),

87 $$\begin{aligned} \left\| \sqrt{\phi _{\epsilon }} \frac{\partial _{x}^{2}\tilde{V}_{1}}{v_{\epsilon }-1}\right\| _{L^{2}_{t}L^{\infty }_{x}} \le \Vert \sqrt{\phi _{\epsilon }}\partial _{x}\tilde{\eta }_{1}\Vert _{L^{2}_{t}L^{\infty }_{x}} + \left\| \frac{v_\epsilon '}{(v_\epsilon -1)^2}\right\| _{L^\infty _{t,x}}\Vert \sqrt{\phi _{\epsilon }}\partial _{x}\tilde{V}_{1}\Vert _{L^{2}_{t}L^{\infty }_{x}} \le \frac{C}{\epsilon ^{3/2}}\Vert \tilde{V}_{1}\Vert _{\mathcal {X}}. \end{aligned}$$

On the other hand (53) also gives us

88 $$\begin{aligned} \left\| \frac{\partial _{x}^{2}V_{1}}{v_\epsilon -1}\right\| _{L^{\infty }_{t}L^{2}_{x}} \le C\Vert \partial _{x}\eta _{1}\Vert _{L^{\infty }_{t}L^{2}_{x}} + \left\| \frac{v_\epsilon '}{(v_\epsilon -1)^2}\right\| _{L^\infty _{t,x}}\Vert \eta _1\Vert _{L^{\infty }_{t}L^{2}_{x}} \le \frac{C}{\epsilon ^{2}} \Vert V_{1}\Vert _{\mathcal {X}}, \end{aligned}$$

and so

$$\begin{aligned} M_{3} \le \frac{C}{\epsilon ^{11/2}} \Vert \tilde{V}_{1}\Vert _{\mathcal {X}} \Vert \tilde{V}_{2}\Vert _{\mathcal {X}}( \Vert V_{1}\Vert _{\mathcal {X}} + \Vert V_{1}'\Vert _{\mathcal {X}}) . \end{aligned}$$

For $M_{4}$, we use again (67), thus,

$$\begin{aligned} \begin{aligned} M_{4}&\le C\Vert \sqrt{\phi _{\epsilon }}\partial _{x}^{2}\tilde{\eta }_{2}\Vert _{L^{2}_{t,x}} \Vert \tilde{\eta }_{1}\Vert _{L^{\infty }_{t,x}} \left\| \sqrt{\phi _{\epsilon }} \frac{\partial _{x}^{3}V_{1}}{v_{\epsilon }-1} \right\| _{L^{2}_{t,x}} +C\Vert \sqrt{\phi _{\epsilon }}\partial _{x}^{2}\tilde{\eta }_{2}\Vert _{L^{2}_{t,x}} \Vert \eta _{1}'\Vert _{L^{\infty }_{t,x}} \left\| \sqrt{\phi _{\epsilon }} \frac{\partial _{x}^{3}\tilde{V}_{1}}{v_{\epsilon }-1} \right\| _{L^{2}_{t,x}} \\  &\le \frac{C}{\epsilon ^{11/2}}\Vert \tilde{V}_{1}\Vert _{\mathcal {X}}\Vert \tilde{V}_{2}\Vert _{\mathcal {X}} ( \Vert V_{1}\Vert _{\mathcal {X}} + \Vert V_{1}'\Vert _{\mathcal {X}}) . \end{aligned} \end{aligned}$$

Lastly, using (60) and (88),

$$\begin{aligned} \begin{aligned} M_{5}&\le C \Vert \tilde{\eta }_{1}\Vert _{L^{\infty }_{t,x}} \Vert \sqrt{\phi _{\epsilon }}\partial _{x}^{2}\tilde{\eta }_{2}\Vert _{L^{2}_{t,x}} \left\| \sqrt{\phi _{\epsilon }} \frac{\partial _{x}^{2}V_{1}'}{v_{\epsilon }-1}\right\| _{L^{2}_{t}L^{\infty }_{x}} \left\| \frac{\partial _{x}^{2}V_{1}'}{v_\epsilon -1}\right\| _{L^{\infty }_{t}L^{2}_{x}} \\  &\le \frac{C}{\epsilon ^{7}} \Vert \tilde{V}_{1}\Vert _{\mathcal {X}}\Vert \tilde{V}_{2}\Vert _{\mathcal {X}}\Vert V_{1}'\Vert _{\mathcal {X}}^{2} = \frac{C}{\epsilon ^{11/2}}\Vert \tilde{V}_{1}\Vert _{\mathcal {X}}\Vert \tilde{V}_{2}\Vert _{\mathcal {X}}\Vert V_{1}'\Vert _{\mathcal {X}} \left( \frac{\Vert V_{1}'\Vert _{\mathcal {X}}}{\epsilon ^{3/2}} \right) . \end{aligned} \end{aligned}$$

Finally,

$$\begin{aligned} \epsilon ^4|L_3|&\le \epsilon ^4\frac{C}{\epsilon ^{11/2}}\Vert \tilde{V}_{1}\Vert _{\mathcal {X}}\Vert \tilde{V}_{2}\Vert _{\mathcal {X}} ( \Vert V_{1}\Vert _{\mathcal {X}} + \Vert V_{1}'\Vert _{\mathcal {X}}) \left( 1 + \frac{\Vert V_{1}'\Vert _{\mathcal {X}}}{\epsilon ^{3/2}} \right) \\&\le \frac{C}{\epsilon ^{3/2}}\Vert \tilde{V}_{1}\Vert _{\mathcal {X}}\Vert \tilde{V}_{2}\Vert _{\mathcal {X}} ( \Vert V_{1}\Vert _{\mathcal {X}} + \Vert V_{1}'\Vert _{\mathcal {X}}) \left( 1 + \frac{\Vert V_{1}'\Vert _{\mathcal {X}}}{\epsilon ^{3/2}} \right) \end{aligned}$$

Gathering our estimates for $L_1, L_2, L_3$ and returning to (79), using the fact that $V_1,V_1'\in B_\delta$, we get

89 $$\begin{aligned} \begin{aligned} \Vert \tilde{V}_{2}\Vert _{\mathcal {X}}^{2}&\le \frac{C}{\epsilon ^{3/2}}\Vert \tilde{V}_{1}\Vert _{\mathcal {X}}\Vert \tilde{V}_{2}\Vert _{\mathcal {X}} ( \Vert V_{1}\Vert _{\mathcal {X}} + \Vert V_{1}'\Vert _{\mathcal {X}}) \left( 1 + \frac{\Vert V_{1}'\Vert _{\mathcal {X}}}{\epsilon ^{3/2}} \right) \\  &\le \delta C \Vert \tilde{V}_{1}\Vert _{\mathcal {X}}\Vert \tilde{V}_{2}\Vert _{\mathcal {X}}. \end{aligned} \end{aligned}$$

Therefore taking $\delta < 1/C$ we obtain that $\mathcal {A}^{\epsilon }$ is a contraction, as required. This concludes the proof of Proposition 3.5.

## Asymptotic Stability of $(u_{\epsilon }, v_{\epsilon })$

So far, we have proved that if (*u*, *v*) is a solution to the original system such that $v - v_{\epsilon }, u - u_{\epsilon } \in L^{1}_{0}(\mathbb {R})$ for all positive times then the system can be re-expressed in terms of the integrated quantities $(V,W_0)$, as (24). Furthermore, provided the initial energy is small enough (i.e. satisfying (47)) then there exists a unique strong solution to (24). In order to prove the existence and uniqueness of strong solutions to the original system (1), we will need the following result:

### Proposition 4.1

Provided that

90 $$\begin{aligned}&(u-u_{\epsilon })(0) \in L^{1}_{0}(\mathbb {R}) \cap H^{1}(\mathbb {R}),\quad \frac{\partial _x(u-u_\epsilon )(0)}{v_\epsilon -1}\in L^2(\mathbb {R}), \end{aligned}$$

91 $$\begin{aligned}&(v - v_{\epsilon })(0) \in L^{1}_{0}(\mathbb {R}), \end{aligned}$$

we have

1. $$\Vert u-u_{\epsilon }\Vert _{L^{\infty }(0,t; L^{1}(\mathbb {R}))} + \Vert v-v_{\epsilon }\Vert _{L^{\infty }(0,t; L^{1}(\mathbb {R}))} \le C(t, \epsilon ), $$
2. $$(u-u_{\epsilon })(t), (v-v_{\epsilon })(t) \in L^{1}_{0}(\mathbb {R})$$ for each $$t > 0$$.

Our strategy of proof is as follows.

- **Step 1:** Establish the stability of the profile $u_{\epsilon }$. This involves bounding the quantity $u-u_{\epsilon }$ in $L^{\infty }_{t}H^{1}_{x}$, which will in particular be needed for the next steps.
- **Step 2:** Use the previous estimates to show that $u-u_{\epsilon }$ and $v-v_{\epsilon }$ remain in $L^{1}$ for positive times.
- **Step 3:** Complete the remaining part of Proposition 4.1 using the estimates from the previous steps.

For the remainder of this section we define $v := v_{\epsilon } + \partial _{x}V$, $w := w_{\epsilon } + \partial _{x}W_0$ and $u:= w + \phi _{\epsilon }(v) \partial _{x}v$. Recall that $\eta = \partial _x V/(v_\epsilon -1)$ is bounded, with $\Vert \eta \Vert _{L^\infty _{t,x}} \le C\epsilon ^{-3/2}\Vert V\Vert _{\mathcal {X}}\le C\delta$ since $V\in B_\delta$, hence $v\ge 1+(1-C\delta )(v_\epsilon -1)>1$ (up to reducing $\delta$) and $\phi _\epsilon (v)$ is well defined. As a consequence, the pair (*w*, *v*) satisfies the system

$$\begin{aligned} \left\{ \begin{aligned}&\partial _t w = 0,\\&\partial _t v -\partial _x w - \partial _x (\phi _\epsilon (v)\partial _x v)=0. \end{aligned} \right. \end{aligned}$$

### Stability of the Profile $u-u_{\epsilon }$

From the system solved by (*w*, *v*), we deduce that *u* is a solution of

92 $$\begin{aligned} \partial _t (u - u_\epsilon ) - \partial _x(\phi _\epsilon (v_\epsilon )\partial _{x}(u-u_{\epsilon })) = \partial _x[(\phi _\epsilon (v)-\phi _\epsilon (v_\epsilon ))\partial _x u] =: G. \end{aligned}$$

We see that the quantity *G* appearing on the right-hand side depends on the difference $\phi _\epsilon (v)-\phi _\epsilon (v_\epsilon )$. In order to control *G*, we will make use of the following lemma, which is analogous to Lemma 2.4.

#### Lemma 4.2

Let $\Delta \phi _\epsilon (f) := \phi _\epsilon (v_{\epsilon }+f) - \phi _\epsilon (v_{\epsilon })$. Then we have

93 $$\begin{aligned}&|\Delta \phi _\epsilon (f)| \le C \phi _{\epsilon }(v_{\epsilon }) \frac{|f|}{v_{\epsilon }-1}, \end{aligned}$$

94 $$\begin{aligned}&|\partial _{x}\Delta \phi _\epsilon (f)| \le C \phi _{\epsilon }(v_{\epsilon })\left( \frac{\partial _{x}v_{\epsilon }|f|}{(v_{\epsilon }-1)^{2}} + \frac{|\partial _{x}f|}{v_{\epsilon }-1} \right) , \end{aligned}$$

95 $$\begin{aligned}&|\partial _{x}^{2}\Delta \phi _\epsilon (f)| \le C \phi _{\epsilon }(v_{\epsilon })\left( \frac{(\partial _{x}v_\epsilon )^{2}|f|}{(v_{\epsilon }-1)^{3}} + \frac{\partial _{x}v_{\epsilon } |\partial _x f|}{(v_{\epsilon }-1)^2} + \left| \frac{\partial _{x}f}{v_{\epsilon }-1} \right| ^{2} + \frac{|\partial _{x}^{2}f|}{v_{\epsilon }-1} \right) . \end{aligned}$$

As a consequence, we also have that

96 $$\begin{aligned} |\phi _{\epsilon }(v)| =|\phi _\epsilon (v_\epsilon +\partial _x V)| \le |\phi _{\epsilon }(v) - \phi _{\epsilon }(v_{\epsilon })| + \phi _{\epsilon }(v_{\epsilon }) \le (|\eta | + 1)\phi _{\epsilon }(v_{\epsilon }). \end{aligned}$$

We omit the proof since it follows the same argument as that of Lemma 2.4. We now wish to prove the following existence result.

#### Lemma 4.3

Suppose that $(U_{0}, V_{0}) \in H^{2}(\mathbb {R}) \times H^{3}(\mathbb {R})$ is such that (47) is satisfied by $(W_{0}, V_{0})$, and that (90) is also satisfied. Consider the solution $(W_0,V) \in B_{\delta } \subset \mathcal {X}$ of (24) obtained in Proposition 3.5. Then there exists a unique solution $u-u_{\epsilon }$ to (92) such that

97 $$\begin{aligned} u-u_{\epsilon } \in C([0,T]; H^{1}(\mathbb {R})) \cap L^{2}(0,T; H^{2}(\mathbb {R})). \end{aligned}$$

Moreover, $$u-u_{\epsilon }$$ satisfies

98 $$\begin{aligned} \begin{aligned} \sup _{t\in [0,T]}\left( \sum _{k=0}^{1} \left[ E_{k}(t; u-u_{\epsilon }) + \int _{0}^{t} D_{k}(\tau ; u-u_{\epsilon })~d\tau \right] \right) \le C, \end{aligned} \end{aligned}$$

where $$C=C(\epsilon ,\delta ,v_+,\gamma ,s)$$.

#### Proof

Recall that the bounds on *V*, *W* (and therefore on *v*, *w*) obtained previously are sufficient to ensure that $u-u_\epsilon \in C([0,T],H^1_{loc}(\mathbb {R}))\cap L^2(0,T;H^2_{loc}(\mathbb {R}))$. The true purpose of Lemma 4.3 is thus the derivation of estimate (98). We start from the equation satisfied by $u-u_{\epsilon }$:

$$\begin{aligned} \partial _t (u - u_\epsilon ) - \partial _x(\phi _\epsilon (v_\epsilon )\partial _{x}(u-u_{\epsilon })) = \partial _x[(\phi _\epsilon (v)-\phi _\epsilon (v_\epsilon ))\partial _x u] = G. \end{aligned}$$

Multiplying by $u-u_{\epsilon }$ and integrating by parts where appropriate, we have

$$\begin{aligned} \begin{aligned} \frac{1}{2} \int _{\mathbb {R}} |u-u_{\epsilon }|^{2}(t)~dx + \int _{0}^{t}\int _{\mathbb {R}} \phi _\epsilon (v_\epsilon ) |\partial _{x}(u-u_{\epsilon })|^{2}~dxd\tau&- \frac{1}{2} \int _{\mathbb {R}} |u-u_{\epsilon }|^{2}(0)~dx \\  &= - \int _{0}^{t}\int _{\mathbb {R}} \partial _{x}(u-u_{\epsilon }) (\phi _{\epsilon }(v) - \phi _{\epsilon }(v_{\epsilon }))\partial _{x}u~dxd\tau . \end{aligned} \end{aligned}$$

With (93) and the triangle inequality, the right-hand side can be bounded by

$$\begin{aligned} \left| \int _{0}^{t}\int _{\mathbb {R}} \partial _{x}(u-u_{\epsilon }) (\phi _{\epsilon }(v) - \phi _{\epsilon }(v_{\epsilon }))\partial _{x}u\right| \le&C\Vert \eta \Vert _{L^\infty _{t,x}}\Vert \sqrt{\phi _\epsilon }\partial _x(u-u_\epsilon )\Vert _{L^2_{t,x}}\Vert \sqrt{\phi _\epsilon }\partial _x u\Vert _{L^2_{t,x}} \\ \le&C\Vert \eta \Vert _{L^\infty _{t,x}}\Vert \sqrt{\phi _\epsilon }\partial _x(u-u_\epsilon )\Vert _{L^2_{t,x}}^2 + C\Vert \eta \Vert _{L^\infty _{t,x}}\Vert \sqrt{\phi _\epsilon }\partial _xu_\epsilon \Vert _{L^2_{t,x}}^2 \\ \le&C\delta \Vert \sqrt{\phi _\epsilon }\partial _x(u-u_\epsilon )\Vert _{L^2_{t,x}}^2 + C(\delta ,v_+,s,\epsilon ), \end{aligned}$$

by using $\partial _x u_\epsilon = -s\partial _x v_\epsilon$ and $\Vert \eta \Vert _\infty \le \delta$. Hence we obtain the following estimate (up to reducing $\delta$):

$$\begin{aligned} \begin{aligned} \int _{\mathbb {R}} |u-u_{\epsilon }|^{2}(t)~dx + \int _{0}^{t}\int _{\mathbb {R}} \phi _\epsilon (v_\epsilon ) |\partial _{x}(u-u_{\epsilon })|^{2}~dx \le \int _{\mathbb {R}} |u-u_{\epsilon }|^{2}(0)~dx + C(\delta ,v_+,s,\epsilon ). \end{aligned} \end{aligned}$$

We now wish to find an estimate for $\partial _{x}(u-u_\epsilon )$. Notice that (92) has the same structure as the equation for *V* (i.e. (32)), up to the source term. As we did for *V*, we define

99 $$\begin{aligned} \mu := \mu (\partial _x u) = \frac{\partial _x(u-u_\epsilon )}{v_\epsilon -1}. \end{aligned}$$

The computations that we did for *V* imply that $\mu$ is solution of

100 $$\begin{aligned} \partial _t \mu -\partial _x(\phi _\epsilon (v_\epsilon ) \partial _x \mu ) + \mathcal {L}_\epsilon (\mu ) = \frac{\partial _x G}{v_\epsilon -1}, \end{aligned}$$

where $\mathcal {L}_\epsilon$ is the same as in Equation (36). Multiplying by $\mu$ and integrating yields

101 $$\begin{aligned} \frac{1}{2}\int _{\mathbb {R}}\mu ^2(t) +\int _0^t\int _{\mathbb {R}}\phi _\epsilon (v_\epsilon )(\partial _x\mu )^2 = \frac{1}{2}\int _{\mathbb {R}}\mu ^2|_{t=0} -\int _0^t\int _{\mathbb {R}}\mathcal {L}_\epsilon (\mu )\mu +\int _0^t\int _{\mathbb {R}}\mu \frac{\partial _x G}{v_\epsilon -1}. \end{aligned}$$

By (38), for $\alpha =1/2$, there exists $C>0$ such that

$$\begin{aligned} \left| \int _0^t\int _{\mathbb {R}}\mathcal {L}_\epsilon (\mu )\mu \right| \le \frac{1}{2} \int _0^t\int _{\mathbb {R}}\phi _\epsilon (v_\epsilon )(\partial _x\mu )^2 + C \int _0^t\int _{\mathbb {R}}\phi _\epsilon (v_\epsilon )[\partial _x(u-u_\epsilon )]^2. \end{aligned}$$

The first integral can be absorbed by the left-hand side of (101). The second integral can be bounded by using the energy estimate satisfied by $u-u_\epsilon$. Hence it is enough to bound the last integral in the right-hand side of (101). We compute that

$$\begin{aligned} \int _0^t\int _{\mathbb {R}}\mu \frac{\partial _x G}{v_\epsilon -1} =&-\int _0^t\int _{\mathbb {R}}\partial _x\mu \frac{G}{v_\epsilon -1} + \int _0^t\int _{\mathbb {R}}\mu \frac{\partial _x v_\epsilon G}{(v_\epsilon -1)^2} \\ =&-\int _0^t\int _{\mathbb {R}}\partial _x\mu \partial _x(\phi _\epsilon (v)-\phi _\epsilon (v_\epsilon ))\mu - \int _0^t\int _{\mathbb {R}} \partial _x \mu \partial _x(\phi _\epsilon (v)-\phi _\epsilon (v_\epsilon ))\frac{\partial _x u_\epsilon }{v_\epsilon -1}\\&- \int _0^t\int _{\mathbb {R}}\partial _x\mu (\phi _\epsilon (v)-\phi _\epsilon (v_\epsilon ))\frac{\partial _x^2(u-u_\epsilon )}{v_\epsilon -1} -\int _0^t\int _{\mathbb {R}}\partial _x\mu (\phi _\epsilon (v)-\phi _\epsilon (v_\epsilon ))\frac{\partial _x^2 u_\epsilon }{v_\epsilon -1} \\&+\int _0^t\int _{\mathbb {R}}\frac{\mu \partial _x v_\epsilon }{v_\epsilon -1} \partial _x(\phi _\epsilon (v)-\phi _\epsilon (v_\epsilon ))\mu +\int _0^t\int _{\mathbb {R}} \frac{\mu \partial _x v_\epsilon }{v_\epsilon -1} \partial _x(\phi _\epsilon (v)-\phi _\epsilon (v_\epsilon ))\frac{\partial _x u_\epsilon }{v_\epsilon -1}\\&+ \int _0^t\int _{\mathbb {R}}\frac{\mu \partial _x v_\epsilon }{(v_\epsilon -1)^2}(\phi _\epsilon (v)-\phi _\epsilon (v_\epsilon ))\partial _x^2(u-u_\epsilon ) + \int _0^t\int _{\mathbb {R}}\frac{\mu \partial _x v_\epsilon }{(v_\epsilon -1)^2}(\phi _\epsilon (v)-\phi _\epsilon (v_\epsilon ))\partial _x^2 u_\epsilon \\ =:&\sum _{k=1}^8 I_k. \end{aligned}$$

By (94) and Young’s inequality, for any $\alpha >0$,

$$\begin{aligned} |I_1| \le&C\int _0^t\int _{\mathbb {R}}\phi _\epsilon (v_\epsilon )|\mu \partial _x\mu |\left( \frac{\partial _x v_\epsilon |\eta |}{v_\epsilon -1}+ \frac{|\partial _{x}^{2} V|}{v_\epsilon -1}\right) \\ \le&C\Vert \sqrt{\phi _\epsilon }\partial _x\mu \Vert _{L^2_{t,x}}\left( \frac{1}{\epsilon }\Vert \sqrt{\phi _\epsilon }\partial _x(u-u_\epsilon )\Vert _{L^2_{t,x}}\Vert \eta \Vert _{L^\infty _{t,x}}+\Vert \mu \Vert _{L^\infty _{t} L^2_{x}}\left\| \sqrt{\phi _\epsilon }\frac{\partial _{x}^{2}V}{v_\epsilon -1}\right\| _{L^2_{t} L^\infty _{x}}\right) \\ \le&\alpha \Vert \sqrt{\phi _\epsilon }\partial _x\mu \Vert _{L^2_{t,x}}^2 + \frac{C\delta ^2}{\alpha }\left( \frac{1}{\epsilon ^2}\Vert \sqrt{\phi _\epsilon }\partial _x(u-u_\epsilon )\Vert _{L^2_{t,x}}^2 + \Vert \mu \Vert _{L^\infty _{t} L^2_{x}}^2\right) , \end{aligned}$$

where the last line uses the fact that

$$\begin{aligned} \Vert \eta \Vert _{L^\infty } + \left\| \sqrt{\phi _\epsilon }\frac{\partial _x^2V}{v_\epsilon -1}\right\| _{L^2_tL^\infty _x}\le \frac{C}{\epsilon ^{3/2}}\delta \epsilon ^{3/2}\le C\delta . \end{aligned}$$

(see Equation (87)). Similarly,

$$\begin{aligned} |I_2| \le ~&C\int _0^t\int _{\mathbb {R}}\phi _\epsilon (v_\epsilon )|\partial _x\mu |\left( \frac{\partial _x v_\epsilon |\eta |}{v_\epsilon -1}+ \frac{|\partial _{x}^{2} V|}{v_\epsilon -1}\right) \frac{\partial _x u_\epsilon }{v_\epsilon -1} \\ \le&\alpha \Vert \sqrt{\phi _\epsilon }\partial _x\mu \Vert _{L^2_{t,x}}^2 + \frac{C}{\alpha }\left( \Vert \sqrt{\phi _\epsilon }\partial _x V\Vert _{L^2_{t,x}}^2\Vert \frac{\partial _x v_\epsilon }{(v_\epsilon -1)^{2}}\Vert ^2_{L^\infty _{t,x}}+\Vert \sqrt{\phi _\epsilon }\frac{\partial _{x}^{2}V}{v_\epsilon -1}\Vert ^2_{L^2_{t,x}}\right) \Vert \frac{\partial _x u_\epsilon }{v_\epsilon -1}\Vert ^2_{L^\infty _{t,x}} \\ \le&\alpha \Vert \sqrt{\phi _\epsilon }\partial _x\mu \Vert _{L^2_{t,x}}^2 + C(\alpha ,\epsilon ,\delta ,s,v_+,\gamma ). \end{aligned}$$

By (93),

$$\begin{aligned} |I_3| \le ~&C\int _0^t\int _{\mathbb {R}}\phi _\epsilon |\partial _x\mu ||\eta |\frac{|\partial _x^2(u-u_\epsilon )|}{v_\epsilon -1} \\ \le&\alpha \Vert \sqrt{\phi _\epsilon }\partial _x\mu \Vert _{L^2_{t,x}}^2 + \frac{C\delta ^2}{\alpha }\left( \Vert \sqrt{\phi _\epsilon }\partial _x\mu \Vert _{L^2_{t,x}}^2 + \frac{1}{\epsilon ^2} \Vert \sqrt{\phi _\epsilon }\partial _x(u-u_\epsilon )\Vert _{L^2_{t,x}}^2\right) \end{aligned}$$

by using

$$\begin{aligned} \frac{\partial ^2_x(u-u_\epsilon )}{v_\epsilon -1} = \partial _x\mu + \frac{\partial _x v_\epsilon \partial _x(u-u_\epsilon )}{(v_\epsilon -1)^2}. \end{aligned}$$

Then,

$$\begin{aligned} |I_4|&\le C\int _0^t\int _{\mathbb {R}} \phi _{\epsilon }|\partial _x\mu | |\eta |\frac{\partial _x^2u_\epsilon }{v_\epsilon -1} \le \alpha \Vert \sqrt{\phi _\epsilon }\partial _x\mu \Vert _{L^2_{t,x}}^2 + \frac{C\delta ^2}{\alpha }\Vert \sqrt{\phi _\epsilon }\frac{\partial _x^2 u_\epsilon }{v_\epsilon -1}\Vert _{L^2_{t,x}}^2 \\&\le \alpha \Vert \sqrt{\phi _\epsilon }\partial _x\mu \Vert _{L^2_{t,x}}^2 + C(\alpha ,\epsilon ,\delta ,s,v_+,\gamma ),\\ |I_5|&\le C \int _0^t\int _{\mathbb {R}}\mu ^2\phi _\epsilon \frac{\partial _x v_\epsilon }{v_\epsilon -1}\left( \frac{\partial _x v_\epsilon |\eta |}{v_\epsilon -1}+ \frac{|\partial _{x}^{2} V|}{v_\epsilon -1}\right) \\&\le \frac{C\delta }{\epsilon ^2} \int _0^t\int _{\mathbb {R}}\phi _\epsilon [\partial _x(u-u_\epsilon )]^2 + \frac{1}{\epsilon }\Vert \mu \Vert _{L^\infty _{t} L^2_{x}}\Vert \sqrt{\phi _\epsilon }\partial _x(u-u_\epsilon )\Vert _{L^2_{t,x}}\left\| \sqrt{\phi _\epsilon }\frac{\partial _x^2V}{v_\epsilon -1}\right\| _{L^2_{t}L^\infty _{x}} \\&\le \alpha \Vert \mu \Vert _{L^\infty _{t} L^2_{x}}^2 + C(\alpha ,\epsilon ,\delta ,s,v_+,\gamma ),\\ |I_6|&\le C \int _0^t\int _{\mathbb {R}}|\mu |\phi _\epsilon \frac{\partial _x v_\epsilon }{v_\epsilon -1}\left( \frac{\partial _x v_\epsilon |\eta |}{v_\epsilon -1}+ \frac{|\partial _{x}^{2} V|}{v_\epsilon -1}\right) \frac{\partial _x u_\epsilon }{v_\epsilon -1} \\&\le \frac{C}{\epsilon ^2}\left\| \sqrt{\phi _\epsilon }\partial _x(u-u_\epsilon )\right\| _{L^2_{t,x}}\left( \frac{1}{\epsilon }\left\| \sqrt{\phi _\epsilon }\partial _x V\right\| _{L^2_{t,x}} + \left\| \sqrt{\phi _\epsilon }\frac{\partial _x^2V}{v_\epsilon -1}\right\| _{L^2_{t,x}}\right) \\&\le C(\epsilon ,\delta ,s,v_+,\gamma ),\\ |I_7|&\le C\int _0^t\int _{\mathbb {R}}|\mu |\phi _\epsilon |\eta |\frac{\partial _x v_\epsilon \partial _x^2(u-u_\epsilon )}{(v_\epsilon -1)^2} \\&\le \frac{C}{\epsilon }\Vert \eta \Vert _{L^\infty _{t,x}}\left\| \sqrt{\phi _\epsilon }\partial _x(u-u_\epsilon )\right\| _{L^2_{t,x}}\left( \left\| \sqrt{\phi _\epsilon }\partial _x\mu \right\| _{L^2_{t,x}} + \frac{1}{\epsilon }\left\| \sqrt{\phi _\epsilon }\partial _x(u-u_\epsilon )\right\| _{L^2_{t,x}} \right) \\&\le \alpha \left\| \sqrt{\phi _\epsilon }\partial _x\mu \right\| _{L^2_{t,x}}^2 + C(\alpha ,\epsilon ,\delta ,v_+,s,\gamma ), \end{aligned}$$

and

$$\begin{aligned} |I_8| \le&C\int _0^t\int _{\mathbb {R}}|\mu |\phi _\epsilon |\eta |\frac{\partial _x v_\epsilon |\partial _x^2u_\epsilon |}{(v_\epsilon -1)^2} \le \frac{C}{\epsilon ^3}\left\| \sqrt{\phi _\epsilon }\partial _x(u-u_\epsilon )\right\| _{L^2_{t,x}}\left\| \sqrt{\phi _\epsilon }\partial _x V\right\| _{L^2_{t,x}} \le C(\epsilon ,\delta ,s,v_+,\gamma ). \end{aligned}$$

Hence, coming back to Equation (101), by taking $\alpha >0$ sufficiently small, up to reducing $\delta$, we obtain that there exists $\alpha _0>0$ such that

$$\begin{aligned} \alpha _0\left( \left\| \mu \right\| _{L^\infty _{t} L^2_{x}}^2 + \left\| \sqrt{\phi _\epsilon }\partial _x\mu \right\| _{L^2_{t,x}}^2\right) \le \frac{1}{2}\Vert \mu _0\Vert _{L^2_{x}}^2+C(\alpha _0,\epsilon ,\delta ,s,v_+,\gamma ), \end{aligned}$$

with $$C(\alpha _0,\epsilon ,\delta ,s,v_+,\gamma )\rightarrow +\infty $$ as $$\epsilon \rightarrow 0$$.

With this estimate, the existence and uniqueness of $u-u_{\epsilon }$ is classical. It remains to verify that $u-u_{\epsilon }$ is continuous in time. Let $f \in H^{1}(\mathbb {R})$ with $\Vert f\Vert _{H^{1}(\mathbb {R})} = 1$. Then using $\partial _{t}(v-v_{\epsilon }) = \partial _{x}(u-u_{\epsilon })$, we have

$$\begin{aligned} \begin{aligned} \langle \partial _{t}(v-v_{\epsilon }), f \rangle _{H^{-1} \times H^{1}}&= (\partial _{x}(u-u_{\epsilon }), f) = (u-u_{\epsilon }, \partial _{x}f) \\  &\le \Vert (u-u_{\epsilon })(t)\Vert _{L^{2}_{x}} \end{aligned} \end{aligned}$$

and so $\Vert \partial _t(v-v_\epsilon )\Vert _{L^\infty _t H^{-1}_x}\le \Vert u-u_\epsilon \Vert _{L^\infty _t L^2_x}$. Since $v-v_\epsilon = \partial _x V$, we have that $\partial _{t}\partial _{x}V \in L^{\infty }(0,T; H^{-1}(\mathbb {R}))\subset L^{2}(0,T; H^{-1}(\mathbb {R}))$. Since we also have $\partial _{x}V \in L^{2}(0,T, H^{2}(\mathbb {R}))$, it follows (e.g. from Theorem II.5.13. of [5]) that $\partial _{x}V \in C([0,T]; H^{1}(\mathbb {R}))$ and so $V \in C([0,T]; H^{2}(\mathbb {R}))$. Now since $w = u - \phi _{\epsilon }(v)\partial _x v$ and $w_{\epsilon } = u_{\epsilon } - \phi _{\epsilon }(v_{\epsilon })\partial _x v_\epsilon$, we have that $\partial _{x}W_0 = u-u_{\epsilon } + \phi _{\epsilon }'(v)\partial _{x}v - \phi _{\epsilon }'(v_{\epsilon })\partial _{x}v_{\epsilon }$. After rearranging, we find that

$$\begin{aligned} u - u_{\epsilon } = \partial _{x}W_0 - \phi _{\epsilon }'(v)\partial _{x}V + (\phi _{\epsilon }'(v) - \phi _{\epsilon }'(v_{\epsilon }))\partial _{x}v_{\epsilon }. \end{aligned}$$

The continuity in time of $W_0, v_{\epsilon }, \phi _\epsilon '(v)$ and *V* imply that $u- u_{\epsilon } \in C([0,T]; H^{1}(\mathbb {R}))$ as claimed. $\square$

### Bounding $(v-v_{\epsilon })$ in $L^{1}(\mathbb {R})$

We first obtain a $L^{1}_{x}$ bound for $u-u_{\epsilon }$. In order to do so, we follow [16], where analogous bounds are derived. We thus introduce $\{j_{n}\}_{n \in \mathbb {N}} \subset C^{2}(\mathbb {R})$ a convex approximation of $| \cdot |$ with $|j_{n}'| \le C, ~j_{n}'' > 0$. For example, we may take $j_{n}(z) := \sqrt{z^{2} + n^{-1}}-\sqrt{n^{-1}}$. Multiplying (92) by $j_{n}'(u-u_{\epsilon })$, we get

102 $$\begin{aligned} \begin{aligned}&\int _{\mathbb {R}} j_{n}(u-u_{\epsilon })(t)~dx-\int _{\mathbb {R}} j_{n}(u-u_{\epsilon })(0)~dx + \int _{0}^{t} \int _{\mathbb {R}} j_{n}''(u-u_{\epsilon }) \phi _{\epsilon }(v) |\partial _{x}(u-u_{\epsilon })|^{2} \\  &\quad = \int _{0}^{t} \int _{\mathbb {R}} j_{n}'(u-u_{\epsilon }) \left( (\phi _{\epsilon }(v)-\phi _{\epsilon }(v_{\epsilon })) \partial _{x}^{2}u_{\epsilon } + (\partial _{x}\phi _{\epsilon }(v) - \partial _{x}\phi _{\epsilon }(v_{\epsilon }))\partial _{x}u_{\epsilon } \right) . \end{aligned} \end{aligned}$$

Using $\partial _{x}u_{\epsilon } = -s\partial _{x}v_{\epsilon }$, (93), (94) and (60), the right-hand side can be bounded by

$$\begin{aligned} \begin{aligned}&C\int _{0}^{t} \int _{\mathbb {R}} \phi _{\epsilon }(v_{\epsilon }) \partial _{x}V \frac{|\partial _{x}^{2}v_{\epsilon }|}{v_{\epsilon }-1} + C\int _{0}^{t} \int _{\mathbb {R}} \phi _{\epsilon }(v_{\epsilon }) \left( \frac{\partial _{x}v_{\epsilon } |\partial _{x}V|}{(v_{\epsilon }-1)^{2}} + \frac{|\partial _{x}^{2}V|}{v_{\epsilon }-1} \right) \partial _{x}v_{\epsilon } \\&\quad \le C\Vert \sqrt{\phi _{\epsilon }}\partial _{x}V\Vert _{L^{2}_{t,x}} \left\| \sqrt{\phi _{\epsilon }}\frac{\partial _{x}^2 v_\epsilon }{v_\epsilon -1}\right\| _{L^{2}_{t,x}} \\  &\qquad + C\Vert \sqrt{\phi _{\epsilon }}\partial _{x}v_{\epsilon }\Vert _{L^{2}_{t,x}} \left( \Vert \sqrt{\phi _{\epsilon } }\frac{\partial _{x}^{2}V}{v_{\epsilon }-1}\Vert _{L^{2}_{t,x}} + \left\| \frac{v_\epsilon '}{(v_\epsilon -1)^2}\right\| _{L^\infty _{t,x}}\Vert \sqrt{\phi _{\epsilon }}\partial _{x}V\Vert _{L^{2}_{t,x}}\right) . \end{aligned} \end{aligned}$$

By Lemma 2.3,

$$\begin{aligned} \left\| \sqrt{\phi _{\epsilon }}\frac{\partial _{x}^2 v_\epsilon }{v_\epsilon -1}\right\| _{L^{2}_{t,x}} + \Vert \sqrt{\phi _{\epsilon }}\partial _{x}v_{\epsilon }\Vert _{L^{2}_{t,x}} \le C(s,\gamma ,v_+,\epsilon )t^{1/2}, \end{aligned}$$

Hence we obtain a global bound of the form $C(\epsilon ,\delta ,s,v_+,\gamma )t^{1/2}$ for the right-hand side. Note that the second term on the left-hand side of (102) has a positive sign and therefore can be discarded. Returning to (102) and taking $n \rightarrow \infty$, we get

103 $$\begin{aligned} \begin{aligned} \Vert (u-u_{\epsilon })(t)\Vert _{L^{1}_{x}} \le \Vert (u-u_{\epsilon })(0)\Vert _{L^{1}_{x}} + C(\epsilon ,\delta ,s,v_+,\gamma )t^{1/2}. \end{aligned} \end{aligned}$$

We now wish to find an estimate for $v-v_{\epsilon }$. From (5) we have that

$$\begin{aligned} \partial _{t}(v-v_{\epsilon }) = \partial _{x}(u-u_{\epsilon }). \end{aligned}$$

Multiplying this equation by $j_{n}'(v-v_{\epsilon })$ where $j_{n}$ is defined as above and integrating in space, we have

104 $$\begin{aligned} \frac{d}{dt}\int _{\mathbb {R}} j_{n}(v-v_{\epsilon })~dx = \int _{\mathbb {R}} j_{n}'(v-v_{\epsilon }) \partial _{x}(u-u_{\epsilon })~dx \le \int _{\mathbb {R}} |\partial _{x}(u-u_{\epsilon })|~dx. \end{aligned}$$

Therefore, to obtain a $L^{1}(\mathbb {R})$ estimate for $v-v_{\epsilon }$ it is sufficient to bound $\partial _{x}(u-u_{\epsilon })$ in $L^{1}(\mathbb {R})$. Now, the evolution equation of $\partial _{x}(u-u_{\epsilon })$ can be expressed as

$$\begin{aligned} \partial _{t}\partial _{x}(u-u_{\epsilon }) - \partial _{x}(\phi _\epsilon (v)\partial _{x}^{2}(u-u_{\epsilon }))&= \partial _{x}\left( (\phi _\epsilon (v)-\phi _\epsilon (v_{\epsilon })) \partial _{x}^{2}u_{\epsilon } \right) + \partial _{x}[\partial _{x}(\phi _\epsilon (v)-\phi _\epsilon (v_\epsilon ))\partial _{x}u] \\  &~~~+ \partial _{x}[\partial _{x}\phi _\epsilon (v_\epsilon )\partial _{x}(u-u_{\epsilon })]. \end{aligned}$$

Multiplying by $j_{n}'(\partial _{x}(u-u_{\epsilon }))$ and integrating by parts where appropriate,

105 $$\begin{aligned} \begin{aligned}&\int _{\mathbb {R}} j_{n}(\partial _{x}(u-u_{\epsilon }))(t)~dx - \int _{\mathbb {R}} j_{n}(\partial _{x}(u-u_{\epsilon }))(0)~dx + \int _{0}^{t} \int _{\mathbb {R}} j_{n}''(\partial _{x}(u-u_{\epsilon })) ~\phi _\epsilon (v) |\partial _{x}^{2}(u-u_{\epsilon })|^{2} \\  &\quad = \int _{0}^{t} \int _{\mathbb {R}} j_{n}'(\partial _{x}(u-u_{\epsilon })) \partial _{x}\left( (\phi _\epsilon (v)-\phi _\epsilon (v_{\epsilon }))\partial _{x}^{2}u_{\epsilon })\right) +\int _{0}^{t} \int _{\mathbb {R}} j_{n}'(\partial _{x}(u-u_{\epsilon })) \partial _{x}[\partial _{x}(\phi _\epsilon (v)-\phi _\epsilon (v_\epsilon ))\partial _{x}u] \\  &\qquad + \int _{0}^{t} \int _{\mathbb {R}} j_{n}'(\partial _{x}(u-u_{\epsilon })) \partial _{x} \left[ \partial _{x}\phi _\epsilon (v_\epsilon )\partial _{x}(u-u_{\epsilon } )\right] =: \sum _{n=1}^{3}I_{n}. \end{aligned} \end{aligned}$$

Firstly using (94),

$$\begin{aligned} \begin{aligned} |I_{1}|&= \left| \int _{0}^{t} \int _{\mathbb {R}} j_{n}'(\partial _{x}(u-u_{\epsilon })) \left( (\partial _{x}\phi _\epsilon (v)-\partial _{x}\phi _\epsilon (v_{\epsilon })) \partial _{x}^{2}u_{\epsilon } + (\phi _\epsilon (v)-\phi _\epsilon (v_{\epsilon }) \partial _{x}^{3}u_{\epsilon } \right) \right| \\  &\le C\int _{0}^{t} \int _{\mathbb {R}} |j_{n}'(\partial _{x}(u-u_{\epsilon }))| \phi _{\epsilon }(v_{\epsilon }) \left( |\partial _{x}^{2}v_{\epsilon }| \left( \frac{\partial _{x}v_{\epsilon }|\partial _{x}V|}{(v_{\epsilon }-1)^{2}} + \frac{|\partial _{x}^{2}V|}{v_{\epsilon }-1} \right) + \frac{|\partial _{x}^{3}v_{\epsilon }|}{v_{\epsilon }-1} |\partial _{x}V| \right) \\  &\le \left\| \sqrt{\phi _{\epsilon }} \frac{\partial _{x}^{3}v_{\epsilon }}{v_{\epsilon }-1}\right\| _{L^{2}_{t,x}}\Vert \sqrt{\phi _{\epsilon }}\partial _{x}V\Vert _{L^{2}_{t,x}} + \left\| \sqrt{\phi _{\epsilon }} \frac{\partial _{x}^{2}v_{\epsilon }}{v_{\epsilon }-1}\right\| _{L^{2}_{t,x}}\left( \frac{C}{\epsilon } \Vert \sqrt{\phi _{\epsilon }}\partial _{x}V\Vert _{L^{2}_{t,x}} + \Vert \sqrt{\phi _{\epsilon }}\partial _{x}^{2}V\Vert _{L^{2}_{t,x}} \right) \\&\le C(\epsilon ,\delta ,s,v_+,\gamma )t^{1/2}. \end{aligned} \end{aligned}$$

Then

$$\begin{aligned} I_2 = \int _0^t\int _{\mathbb {R}}j_n'(\partial _{x}(u-u_{\epsilon }))\partial _x^2[\phi _\epsilon (v)-\phi _\epsilon (v_\epsilon )]\partial _x u + \int _0^t\int _{\mathbb {R}}j_n'(\partial _{x}(u-u_{\epsilon }))\partial _x[\phi _\epsilon (v)-\phi _\epsilon (v_\epsilon )]\partial _x^2 u =: K_1 +K_2. \end{aligned}$$

Using (95),

$$\begin{aligned} |K_1|\le&C\int _0^t\int _{\mathbb {R}}\phi _{\epsilon }(v_{\epsilon })\left( \frac{(\partial _{x}v_\epsilon )^{2}|\partial _x V|}{(v_{\epsilon }-1)^{3}} + \frac{\partial _{x}v_{\epsilon } |\partial _x^2 V|}{(v_{\epsilon }-1)^2} + \left| \frac{\partial _{x}^2V}{v_{\epsilon }-1} \right| ^{2} + \frac{|\partial _{x}^{3}V|}{v_{\epsilon }-1} \right) |\partial _x u| \\ \le&C\Vert \sqrt{\phi _\epsilon }\partial _x u\Vert _{L^2_{t,x}}\left( \Vert \sqrt{\phi _\epsilon }\partial _x V\Vert _{L^2_{t,x}}+ \left\| \sqrt{\phi _\epsilon }\frac{\partial _x^2V }{v_\epsilon -1} \right\| _{L^2_{t,x}}+ \left\| \sqrt{\phi _\epsilon }\frac{\partial _x^3V }{v_\epsilon -1} \right\| _{L^2_{t,x}} \right. \\&\left. +\left\| \sqrt{\phi _\epsilon }\frac{\partial _x^2V}{v_\epsilon -1}\right\| _{L^2L^\infty }\left\| \frac{\partial _x^2V}{v_\epsilon -1}\right\| _{L^\infty L^2} \right) , \end{aligned}$$

and the triangle inequality

$$\begin{aligned} \Vert \sqrt{\phi _\epsilon }\partial _x u\Vert _{L^2_{t,x}} \le \Vert \sqrt{\phi _\epsilon }\partial _x (u-u_\epsilon )\Vert _{L^2_{t,x}} + \Vert \sqrt{\phi _\epsilon }\partial _x u_\epsilon \Vert _{L^2_{t,x}} \end{aligned}$$

enables to bound $K_1$. For $K_2$, (94) yields

$$\begin{aligned} |K_2| \le&C\int _0^t\int _{\mathbb {R}}\phi _{\epsilon }(v_{\epsilon })\left( \frac{\partial _{x}v_{\epsilon }|\partial _x V|}{(v_{\epsilon }-1)^{2}} + \frac{|\partial _{x}^2 V|}{v_{\epsilon }-1} \right) |\partial _x^2 u| \\ \le&\left( \left\| \sqrt{\phi _\epsilon }\partial _x V\right\| _{L^2_{t,x}}+\left\| \sqrt{\phi _\epsilon }\frac{\partial _x^2 V}{v_\epsilon -1}\right\| _{L^2_{t,x}}\right) \left( \left\| \sqrt{\phi _\epsilon }\partial _x^2(u-u_\epsilon )\right\| _{L^2_{t,x}}+\left\| \sqrt{\phi _\epsilon }\partial _x^2 u_\epsilon \right\| _{L^2_{t,x}}\right) . \end{aligned}$$

For $I_3$, we decompose

$$\begin{aligned} |I_3| =&C\left| \int _0^t\int _{\mathbb {R}}j_n'\left[ \left( \phi _\epsilon ''(v_\epsilon )(\partial _x v_\epsilon )^2+\phi _\epsilon '(v_\epsilon )\partial _x^2v_\epsilon \right) \partial _x(u-u_\epsilon ) + \phi _\epsilon '(v_\epsilon )\partial _x v_\epsilon \partial _x^2(u-u_\epsilon ) \right] \right| \\ \le&C\left[ \left\| \frac{\phi _\epsilon ''(v_\epsilon )v_\epsilon '}{\phi _\epsilon (v_\epsilon )}\right\| _{L^\infty _{t,x}}\Vert \sqrt{\phi _\epsilon }\partial _x v_\epsilon \Vert _{L^2_{t,x}}+\left\| \frac{\phi _\epsilon '(v_\epsilon )(v_\epsilon -1)}{\phi _\epsilon (v_\epsilon )}\right\| _{L^\infty _{t,x}}\left\| \sqrt{\phi _\epsilon }\frac{\partial _x^2 v_\epsilon }{v_\epsilon -1}\right\| _{L^2_{t,x}}\right] \Vert \sqrt{\phi _\epsilon }\partial _x(u-u_\epsilon )\Vert _{L^2_{t,x}} \\&+ C\left\| \frac{\phi _\epsilon '(v_\epsilon )(v_\epsilon -1)}{\phi _\epsilon (v_\epsilon )}\right\| _{L^\infty _{t,x}}\Vert \sqrt{\phi _\epsilon }\partial _x v_\epsilon \Vert _{L^2_{t,x}}\left\| \sqrt{\phi _\epsilon }\frac{\partial _x^2(u-u_\epsilon )}{v_\epsilon -1}\right\| _{L^2_{t,x}}. \end{aligned}$$

Passing to the limit $n \rightarrow \infty$ with the help of Fatou’s lemma gives us

106 $$\begin{aligned} \Vert \partial _{x}(u-u_{\epsilon })(t)\Vert _{L^{1}_{x}} \le \Vert \partial _{x}(u-u_{\epsilon })(0)\Vert _{L^{1}_{x}} +C(\epsilon ,\delta ,s,v_+,\gamma )t^{1/2}. \end{aligned}$$

We have now shown that $(u-u_{\epsilon })(t), ~(v-v_{\epsilon })(t)$ belong to $L^{1}(\mathbb {R})$ for any $t>0$. Note that since the equations for both quantities are conservative, we actually have that $(u-u_{\epsilon })(t), ~(v-v_{\epsilon })(t) \in L^{1}_{0}(\mathbb {R})$. This marks the end of the proof of Proposition 4.1.

### Concluding the Proofs of Theorem 1.7 and Corollary 1.8

We are now in a position to justify the equivalence between the original and integrated systems, which will allow us to conclude the proof of Theorem 1.7. Firstly, consider the original system with some initial data $(u_{0}, v_{0})$ which satisfies the assumptions of Theorem 1.7, and let (*V*, *W*) be the corresponding solution of the integrated system (24). Since we defined $(u,v) := (u_{\epsilon } + \partial _{x}U, v_{\epsilon } + \partial _{x}V)$, it follows that (*u*, *v*) is a solution to the original system. Moreover, Lemma 4.3 tells us that $\partial _{x}U \in C([0,T]; H^{1}(\mathbb {R}))$ and Theorem 1.2 tells us that $\partial _{x}V \in C([0,T];H^{1}(\mathbb {R})) \cap L^{2}(0,T; H^{2}(\mathbb {R}))$. Thus, $(u,v) \in (u_{\epsilon }, v_{\epsilon }) + (C([0,T];H^{1}(\mathbb {R})))^{2}$ solves the original system and using Proposition 4.1 we have that $(u,v)(t) \in L^{1}_{0}(\mathbb {R})$ for any $t \in (0,T]$. It remains to show that the solution is unique. To this end, suppose that (*u*, *v*) is another solution to the original system with initial data $(u_{0}, v_{0})$ satisfying the assumptions of Theorem 1.7. Then we may write $(u,v) = (u_{\epsilon }, v_{\epsilon }) + (f,g)$ for some $f,g \in C([0,T]; H^{1}(\mathbb {R}) \cap L^{1}_{0}(\mathbb {R}))$. Now, define the integrated quantities

$$\begin{aligned} U(t,x) := \int _{-\infty }^{x} (u(t,z)-u_{\epsilon }(t,z))~dz, \quad V(t,x) := \int _{-\infty }^{x} (v(t,z)-v_{\epsilon }(t,z))~dz, \end{aligned}$$

and

$$\begin{aligned} W := U + \phi _{\epsilon }(v) - \phi _{\epsilon }(v_{\epsilon }). \end{aligned}$$

Then (*V*, *W*) is a solution of the integrated system (24). Furthermore, thanks to the hypotheses on the initial data (*u*, *v*) we have that $\partial _{x}V \in C([0,T]; H^{1}(\mathbb {R}) \cap L^{1}_{0}(\mathbb {R})) \cap L^{2}(0,T; H^{2}(\mathbb {R}))$. Therefore $V \in \mathcal {X}$. Moreover, due to the assumptions on the initial data, we in fact have $V \in B_{\delta }$ and so since $\mathcal {A}^{\epsilon }$ is a contraction on $B_{\delta }$ we easily deduce that *V* is uniquely determined by the initial data. Since *W* is constant in time, we conclude that the pair (*W*, *V*) is unique and therefore so is (*u*, *v*).

Finally, let us explain how Corollary 1.8 is obtained. With the additional assumption (17), the inequality (72) becomes

107 $$\begin{aligned} \begin{aligned} \sup _{t \in [0,T]}&\left( \sum _{k=0}^{2} c_{k}\epsilon ^{2k}\left[ E_{k}(t;V_{2}) + \int _{0}^{t}D_{k}(\tau ;V_{2})~d\tau \right] \right) \\  &\le C( E_{0}(0; V_{2}) + \epsilon ^{2}E_{1}(0;V_{2}) + \epsilon ^{4}E_{2}(0;V_{2})) + \frac{C}{\epsilon ^{3/2}} \Vert V_{1}\Vert _{\mathcal {X}}^{2}\Vert V_{2}\Vert _{\mathcal {X}}, \end{aligned} \end{aligned}$$

and so we may take $T = + \infty$ which gives us a solution defined on $\mathbb {R}_{+} \times \mathbb {R}$. Then using (50) we have that there exists $C > 0$ independent of time such that

108 $$\begin{aligned} \Vert (v-v_{\epsilon })(t)\Vert _{L^{\infty }_{x}} \le C \Vert (v-v_{\epsilon })(t)\Vert _{L^{2}_{x}}^{1/2} \Vert \partial _{x}(v-v_{\epsilon })(t)\Vert _{L^{2}_{x}}^{1/2}, \end{aligned}$$

where the right hand-side tends to 0 as $t \rightarrow \infty$ since $V \in L^{2}(\mathbb {R}_{+}; H^{2}(\mathbb {R}))$. This shows that $(v-v_{\epsilon })(t) \rightarrow 0$ as $t \rightarrow \infty$. Similarly, the bounds obtained in Lemma 4.3 imply that

$$\begin{aligned} \Vert (u-u_{\epsilon })(t)\Vert _{H^{1}_{x}} \rightarrow 0 \text { as } t \rightarrow \infty \end{aligned}$$

and so we also find that $(u-u_{\epsilon })(t) \rightarrow 0$ as $t \rightarrow \infty$, which completes the proof.

## Data Availability

No new data was collected in the course of this research.

## Proof that the Map $\mathcal {A}^\epsilon$ is Well Defined

We give here a proof that the map $\mathcal {A}^\epsilon$ of Section 3.3 is well defined, i.e. we show that Equation (44):

$$\begin{aligned} \left\{ \begin{aligned}&\partial _t V_{2} -\partial _x W_0 -\partial _x(\phi _\epsilon (v_\epsilon )\partial _xV_{2})=\partial _x H(\partial _xV_{1}),\\&V_{2}|_{t=0} = V_{0}, \end{aligned} \right. \end{aligned}$$

is well posed. Equation (44) rewrites as

109 $$\begin{aligned} \left\{ \begin{aligned}&\partial _t V_{2} =\partial _x(\phi _\epsilon \partial _x V_2) +f,\\&V_{2}|_{t=0} = V_{0}, \end{aligned} \right. \end{aligned}$$

where $f=\partial _x H(\partial _x V_1)+\partial _x W_0$. Since the operator $\partial _x(\phi _\epsilon \partial _x\cdot )$ is uniformly elliptic, one can hope that it is the infinitesimal generator of an analytic evolution operator $(U(t,\tau ))_{t,\tau \ge 0}$. The unique solution of (109) is then given by

$$\begin{aligned} V_2(t) = U(t,0)V_0 + \int _0^t U(t,\tau )f(\tau )d\tau . \end{aligned}$$

The proof that $\partial _x(\phi _\epsilon \partial _x\cdot )$ generates an analytic evolution operator is classical. Note that one has to be careful because the coefficient $\phi _\epsilon = \phi _\epsilon (t,x)$ depends on time and is unbounded. In order to simplify the situation, we take advantage of the fact that $\phi _\epsilon$ is a function of the variable $\xi := x-st$. A change of variable $(t,x)\mapsto (t,\xi )\in (0,T)\times \mathbb {R}$ then yields that (109) can be written as

$$\begin{aligned} \left\{ \begin{aligned}&\partial _t V_{2} =\partial _\xi (\phi _\epsilon (\xi ) \partial _\xi V_2) + s\partial _\xi V_2 +f,\\&V_{2}|_{t=0} = V_{0}, \end{aligned} \right. \end{aligned}$$

Hence in these coordinates, the operator $\partial _\xi (\phi _\epsilon (\xi )\partial _\xi \cdot )+s\partial _\xi$ is independant of time. Consider the Hilbert space $H=L^2(\mathbb {R},\mathbb {C})$, and define the unbounded operator

$$\begin{aligned} T_c V:= \phi _\epsilon ^{1/2}\partial _\xi V, \qquad \textrm{with}\qquad D(T_c) = C^\infty _c(\mathbb {R}). \end{aligned}$$

Let *T* be the closure of $T_c$, i.e.

$$\begin{aligned} D(T)= \{V\in H,\phi _\epsilon ^{1/2}\partial _\xi V\in H, \exists V_n \in C^\infty _c(\mathbb {R}), V_n \rightarrow V, \phi _\epsilon ^{1/2}\partial _\xi V_n \rightarrow \phi _\epsilon ^{1/2}\partial _\xi V\}. \end{aligned}$$

The adjoint $T^*$ of *T* is given by

$$\begin{aligned} D(T^*) = \{V\in H, \partial _\xi (\phi _\epsilon ^{1/2}V) \in H\},\qquad \textrm{with} \qquad T^*V = -\partial _\xi (\phi _\epsilon ^{1/2}V). \end{aligned}$$

Indeed, if $V\in D(T^*)$, then $T^*V = -\partial _\xi (\phi _\epsilon ^{1/2}V)$ in the sense of distributions. Conversely, if *V* and $\partial _\xi (\phi _\epsilon ^{1/2}V)\in H$, then $<-\partial _\xi (\phi _\epsilon ^{1/2}V),\psi>_H = <V, T\psi >_H$ for every $\psi \in C^\infty _c(\mathbb {R})$, and the same is true for every $\psi \in D(T)$ by density.

Since *T* is closed and densely defined, $T^*T$ defined by

$$\begin{aligned} D(T^*T) = \{V\in D(T), TV \in D(T^*) \}, \qquad T^*T V = T^*(TV) =-\partial _\xi (\phi _\epsilon (\xi )\partial _x V) \end{aligned}$$

is self-adjoint and positive (see Theorem 13.13 in [21], Chapter 13). It follows that $-T^* T$ is the infinitesimal generator of an analytic semigroup. Now define the closed operator $B:= s\partial _\xi$, with $D(B) = H^1(\mathbb {R})$. Since there exists $\alpha > 0$ such that $\phi _\epsilon \ge \alpha$, we deduce that $D(T^* T)\subset D(T) \subset D(B)$. Furthermore, for every $\epsilon >0$, for every $V\in D(T^* T)$, one has that

$$\begin{aligned} \Vert BV\Vert \le \frac{s}{\alpha }\Vert TV\Vert =&\frac{s}{\alpha } |<V,T^* TV>_H|^{1/2} \le \frac{s}{\alpha }\Vert V\Vert ^{1/2}\Vert T^* TV\Vert ^{1/2} \\ \le&\epsilon \Vert T^* TV\Vert + \frac{s^2}{4\alpha ^2\epsilon }\Vert V\Vert , \end{aligned}$$

by Young’s inequality. By Theorem 12.37 in [20], it follows that $-T^*T+B$ is the infinitesimal generator of an analytic semigroup.

Note that in fact it is possible to prove that

$$\begin{aligned} D(T) = \{ V\in H, \phi _\epsilon ^{1/2}\partial _x V \in H\}. \end{aligned}$$

Indeed, let $\theta$ a smooth function such that $0\le \theta \le 1$, $\theta |_{[-\frac{1}{3},\frac{1}{3}]}\equiv 1$, $\textrm{supp}(\theta )\subset [-1,1]$ and $\int _{\mathbb {R}}\theta =1$. For $V\in H$ with $\phi _\epsilon ^{1/2}\partial _\xi V\in H$, define

$$\begin{aligned} V_n(\xi ) := \theta \left( \frac{\xi }{n}\right) (\rho _n * V)(\xi ), \end{aligned}$$

where $\rho _n := n\theta (n\cdot )\in C^\infty _c(\mathbb {R})$ is an approximation of unity. Then $V_n\in C_c^\infty (\mathbb {R})$ and $V_n\rightarrow V$ in $L^2$. Furthermore,

$$\begin{aligned} \phi _\epsilon ^{1/2}\partial _\xi V_n = \frac{\phi _\epsilon ^{1/2}}{n}\theta '\left( \frac{\xi }{n}\right) (\rho _n * V)(\xi ) + \phi _\epsilon ^{1/2}\theta \left( \frac{\xi }{n}\right) (\rho _n * \partial _\xi V)(\xi ). \end{aligned}$$

Since $\phi _\epsilon ^{1/2}(\xi )\le C(1+|\xi |^{1/2+1/(2\gamma )})$ and $\theta$ is compactly supported,

$$\begin{aligned} \left| \frac{\phi _\epsilon ^{1/2}}{n}\theta '\left( \frac{\xi }{n}\right) \right| \le Cn^{1/(2\gamma )- 1/2}\textbf{1}_{n/3\le |\xi |\le n}. \end{aligned}$$

Hence

$$\begin{aligned} \frac{\phi _\epsilon ^{1/2}}{n}\theta '\left( \frac{\xi }{n}\right) (\rho _n * V)(\xi )\rightarrow 0 \qquad \textrm{and}\qquad \phi _\epsilon ^{1/2}\theta \left( \frac{\xi }{n}\right) (\rho _n * \partial _\xi V)(\xi )\rightarrow \phi _\epsilon ^{1/2}\partial _\xi V. \end{aligned}$$

Hence $$V\in D(T)$$.

## Proof of Lemma 3.3

We give here the proof of Lemma 3.3.

### Proof

We first do the computations of the estimate for $\mathcal {L}_\epsilon$. We compute that

$$\begin{aligned} \int _0^t\int _{\mathbb {R}}\mathcal {L}_\epsilon (\eta )\eta =&\int _{0}^{t}\int _{\mathbb {R}} \frac{s\eta ^{2}v_{\epsilon }'}{v_{\epsilon }-1}~dxd\tau + \int _{0}^{t}\int _{\mathbb {R}} \frac{\phi _{\epsilon }(v_{\epsilon })v_{\epsilon }'}{v_{\epsilon }-1} \eta \left( \partial _{x}\eta + \frac{\eta v_{\epsilon }'}{v_{\epsilon }-1}\right) ~dxd\tau \\&+ \int _{0}^{t} \int _{\mathbb {R}} \eta \partial _{x} \left( \frac{\eta \phi _{\epsilon }(v_{\epsilon })v_{\epsilon }'}{v_{\epsilon }-1}\right) ~dxd\tau + \int _{0}^{t} \int _{\mathbb {R}} \frac{\eta }{v_{\epsilon }-1} \partial _{x}( \phi _{\epsilon }'(v_{\epsilon })v_{\epsilon }'(v_{\epsilon }-1)\eta )~dxd\tau =:\sum _{k=1}^4 I_k. \end{aligned}$$

Let us fix $\alpha \in (0,1)$. For $I_1$, we exploit the smallness of $v_{\epsilon }'$ (i.e. (49)) to get

$$\begin{aligned} |I_{1}| = \left| \int _{0}^{t}\int _{\mathbb {R}} \frac{s\eta ^{2}v_{\epsilon }'}{v_{\epsilon }-1}~dxd\tau \right| \le \left\| \frac{s v_{\epsilon }'}{\phi _{\epsilon }(v_{\epsilon }-1)^{3}}\right\| _{L^{\infty }_{t,x}} \int _{0}^{t} \int _{\mathbb {R}} |\sqrt{\phi _{\epsilon }}\partial _{x}V |^{2} \le \frac{C}{\epsilon ^{2}} \int _{0}^{t}D_{0}(\tau ; V )~d\tau . \end{aligned}$$

Next,

$$\begin{aligned} \begin{aligned} |I_{2}|&= \left| \int _{0}^{t}\int _{\mathbb {R}} \frac{v_{\epsilon }'\phi _{\epsilon }}{v_{\epsilon }-1} \eta \left( \partial _{x}\eta + \frac{\eta v_{\epsilon }'}{v_{\epsilon }-1}\right) ~dxd\tau \right| \\&\le \int _{0}^{t} \int _{\mathbb {R}} \left| \frac{v_{\epsilon }' \phi _{\epsilon }}{v_{\epsilon }-1} \right| |\eta | |\partial _{x}\eta | + \int _{0}^{t} \int _{\mathbb {R}} \left| \frac{v_{\epsilon }' \phi _{\epsilon }}{v_{\epsilon }-1} \frac{v_{\epsilon }' \partial _{x}V }{(v_{\epsilon }-1)^{2}} \frac{\partial _{x}V }{v_{\epsilon }-1} \right| \\  &\le \left\| \frac{v_{\epsilon }'}{(v_{\epsilon }-1)^{2}}\right\| _{L^{\infty }_{t,x}} \Vert \sqrt{\phi _{\epsilon }}\partial _{x}V \Vert _{L^{2}_{t,x}} \Vert \sqrt{\phi _{\epsilon }}\partial _{x}\eta \Vert _{L^{2}_{t,x}} + \left\| \frac{(v_{\epsilon }')^{2}}{(v_{\epsilon }-1)^{4}}\right\| _{L^{\infty }_{t,x}} \Vert \sqrt{\phi _{\epsilon }} \partial _{x}V \Vert _{L^{2}_{t,x}}^{2} \\  &\le \frac{C}{\epsilon } \left( \int _{0}^{t}D_{0}(\tau ; V )~d\tau \right) ^{\frac{1}{2}}\left( \int _{0}^{t}D_{1}(\tau ; V )~d\tau \right) ^{\frac{1}{2}} + \frac{C}{\epsilon ^{2}} \int _{0}^{t} D_{0}(\tau ;V )~d\tau \\  &\le \frac{\alpha }{3} \int _{0}^{t} D_{1}(\tau ; V )~d\tau + \frac{C}{\alpha \epsilon ^{2}} \int _{0}^{t} D_{0}(\tau ; V )~d\tau , \end{aligned} \end{aligned}$$

where we used Young’s inequality for the last line. Similarly,

$$\begin{aligned} \begin{aligned} |I_{3}|&= \left| \int _{0}^{t} \int _{\mathbb {R}} \eta \partial _{x} \left( \frac{\eta \phi _{\epsilon }(v_{\epsilon })v_{\epsilon }'}{v_{\epsilon }-1}\right) ~dxd\tau \right| \\&\le \int _{0}^{t} \int _{\mathbb {R}} |\partial _{x}\eta | \left| \frac{\eta v_{\epsilon }'\phi _{\epsilon }}{v_{\epsilon }-1}\right| \le \left\| \frac{v_{\epsilon }'}{(v_{\epsilon }-1)^{2}} \right\| _{L^{\infty }_{t,x}} \Vert \sqrt{\phi _{\epsilon }}\partial _{x}\eta \Vert _{L^{2}_{t,x}} \Vert \sqrt{\phi _{\epsilon }}\partial _{x}V \Vert _{L^{2}_{t,x}} \\  &\le \frac{\alpha }{3} \int _{0}^{t} D_{1}(\tau ;V )~d\tau + \frac{C}{\alpha \epsilon ^{2}} \int _{0}^{t}D_{0}(\tau ;V )~d\tau . \end{aligned} \end{aligned}$$

To deal with the last term, we first integrate by parts to get

$$\begin{aligned} I_{4} = \int _{0}^{t} \int _{\mathbb {R}} \frac{\eta }{v_{\epsilon }-1} \partial _{x}( \phi _{\epsilon }'(v_{\epsilon })v_{\epsilon }'(v_{\epsilon }-1)\eta )~dxd\tau =- \int _{0}^{t} \int _{\mathbb {R}} \frac{\partial _{x}\eta }{v_{\epsilon }-1} v_{\epsilon }' \phi _{\epsilon }' \partial _{x}V + \int _{0}^{t} \int _{\mathbb {R}} \frac{(v_{\epsilon }')^{2} \eta }{(v_{\epsilon }-1)^{2}} \phi _{\epsilon }' \partial _{x}V . \end{aligned}$$

Then in the same spirit as the previous estimates and with the estimate (48) on $\phi _\epsilon '$,

$$\begin{aligned} \begin{aligned} |I_{4}|&\le \left\| \frac{v_\epsilon '}{(v_\epsilon -1)^2}\right\| _{L^{\infty }_{t,x}} \Vert \sqrt{\phi _{\epsilon }}\partial _{x}\eta \Vert _{L^{2}_{t,x}} \Vert \sqrt{\phi _{\epsilon }}\partial _{x}V \Vert _{L^{2}_{t,x}} + \left\| \frac{(v_{\epsilon }')^{2}}{(v_{\epsilon }-1)^{4}}\right\| _{L^{\infty }_{t,x}}\Vert \sqrt{\phi _{\epsilon }}\partial _{x}V \Vert _{L^{2}_{t,x}}^{2} \\  &\le \frac{\alpha }{3} \int _{0}^{t} D_{1}(\tau ; V )~d\tau + \frac{C}{\alpha \epsilon ^{2}} \int _{0}^{t}D_{0}(\tau ;V )~d\tau . \end{aligned} \end{aligned}$$

We finally put all terms together to obtain that

$$\begin{aligned} \left| \int _0^t\int _{\mathbb {R}}\mathcal {L}_\epsilon (\eta )\eta \right| \le \sum _{k=1}^4 |I_k| \le \alpha \int _0^t D_1(\tau ;V)d\tau + \frac{C}{\alpha \epsilon ^2}\int _0^t D_0(\tau ; V)d\tau . \end{aligned}$$

We now move on to the proof of the second estimate. Again, we fix $\alpha \in (0,1)$ and compute that

110 $$\begin{aligned} \begin{aligned} \int _{0}^{t} \int _{\mathbb {R}}\partial _{x}\eta ~ \mathcal {C}_\epsilon (\eta ) =&-\int _{0}^{t} \int _{\mathbb {R}} \partial _{x}\phi _{\epsilon } \partial _{x}^{2}\eta \partial _{x}\eta + \int _{0}^{t} \int _{\mathbb {R}} \eta \partial _{x} \eta \partial _{x} \left( \frac{sv_{\epsilon }'}{v_{\epsilon }-1} \right) \\&+\int _0^t\int _{\mathbb {R}}(\partial _x \eta )^2\partial _x\left( \phi _\epsilon \frac{v_\epsilon '}{v_\epsilon -1}\right) + \int _{0}^{t} \int _{\mathbb {R}} \eta \partial _{x}\eta \partial _{x} \left( \frac{(v_{\epsilon }')^{2}\phi _{\epsilon }(v_{\epsilon })}{(v_{\epsilon }-1)^{2}}\right) \\  &+ \int _{0}^{t} \int _{\mathbb {R}} \partial _{x}\eta \partial _{x} \left( \eta \partial _{x}\left( \frac{v_{\epsilon }'\phi _{\epsilon }(v_{\epsilon })}{v_{\epsilon }-1}\right) \right) - \int _{0}^{t} \int _{\mathbb {R}} \partial _{x}\eta \frac{v_{\epsilon }' \partial _{x}(\phi _{\epsilon }'(v_{\epsilon }) v_{\epsilon }'(v_{\epsilon }-1) \eta )}{(v_{\epsilon }-1)^{2}} \\  &+ \int _{0}^{t} \int _{\mathbb {R}} \frac{\partial _{x}\eta }{v_{\epsilon }-1} \partial _{x}(\partial _{x}(v_{\epsilon }'\phi _{\epsilon }'(v_{\epsilon })(v_{\epsilon }-1))\eta ) =: \sum _{n=1}^{7}K_{n}. \end{aligned} \end{aligned}$$

We can estimate each of these terms using the same strategy as our estimates for $I_{1}-I_{4}$. This leads to

$$\begin{aligned} \begin{aligned}&|K_{1}|\le \left\| \frac{\partial _x\phi _\epsilon }{\phi _\epsilon }\right\| _{L^\infty _{t,x}}\Vert \sqrt{\phi _\epsilon }\partial _x^2\eta \Vert _{L^2_{t,x}}\Vert \sqrt{\phi _\epsilon }\partial _x\eta \Vert _{L^2_{t,x}} \le \frac{\alpha }{6} \int _{0}^{t} D_{2}(\tau ;V )~d\tau + \frac{C}{\alpha \epsilon ^{2}} \int _{0}^{t} D_{1}(\tau ;V )~d\tau , \\  &|K_{2}| =\left| \int _0^t\int _{\mathbb {R}}\frac{sv_\epsilon '}{v_\epsilon -1}\left( (\partial _x\eta )^2+\eta \partial _x^2\eta \right) \right| \le \frac{\alpha }{6} \int _{0}^{t} D_{2}(\tau ;V )~d\tau + \frac{C}{\epsilon ^{2}} \int _{0}^{t} D_{1}(\tau ;V )~d\tau + \frac{C}{\alpha \epsilon ^{4}} \int _{0}^{t}D_{0}(\tau ;V )~d\tau , \\  &|K_3| =2\left| \int _0^t \phi _\epsilon \frac{v_\epsilon '}{v_\epsilon -1} \partial _x^2\eta \partial _x\eta \right| \le \frac{\alpha }{6} \int _{0}^{t} D_{2}(\tau ;V )~d\tau +\frac{C}{\alpha \epsilon ^2}\int _0^tD_1(\tau ;V)d\tau , \\  &|K_{4} | = \left| \int _0^t\int _{\mathbb {R}}\frac{(v_\epsilon ')^2\phi _\epsilon }{(v_\epsilon -1)^2}\left( \eta \partial _x^2\eta +(\partial _x\eta )^2\right) \right| \le \frac{\alpha }{6} \int _{0}^{t} D_{2}(\tau ;V )~d\tau + \frac{C}{\epsilon ^{2}}\int _{0}^{t} D_{1}(\tau ;V )~d\tau + \frac{C}{\epsilon ^{4}} \int _{0}^{t}D_{0}(\tau ;V )~d\tau , \\&|K_{5}|=\left| \int _0^t\int _{\mathbb {R}}\partial _x\left( \phi _\epsilon \frac{v_\epsilon '}{v_\epsilon -1}\right) \eta \partial _x^2\eta \right| , \end{aligned} \end{aligned}$$

and we compute with the estimate (48) and Lemma 2.1 that

$$\begin{aligned} \left| \partial _x\left( \phi _\epsilon \frac{v_\epsilon '}{v_\epsilon -1}\right) \right| \le \frac{C}{\epsilon ^2}\phi _\epsilon (v_\epsilon -1)^{2\gamma } \le \frac{C}{\epsilon ^2}\phi _\epsilon , \end{aligned}$$

hence

$$\begin{aligned} |K_5|\le \frac{\alpha }{6} \int _{0}^{t} D_{2}(\tau ;V )~d \tau + \frac{C}{\alpha \epsilon ^{4}} \int _{0}^{t}D_{0}(\tau ;V )~d\tau . \end{aligned}$$

Next,

$$\begin{aligned} K_6 = - \int _{0}^{t} \int _{\mathbb {R}} (\partial _{x}\eta )^2 \frac{v_{\epsilon }' \phi _{\epsilon }'(v_{\epsilon }) v_{\epsilon }'(v_{\epsilon }-1) }{(v_{\epsilon }-1)^{2}} -\int _{0}^{t} \int _{\mathbb {R}} \eta \partial _{x}\eta \frac{v_{\epsilon }' \partial _{x}(\phi _{\epsilon }'(v_{\epsilon }) v_{\epsilon }'(v_{\epsilon }-1) )}{(v_{\epsilon }-1)^{2}}. \end{aligned}$$

With the estimate (48) and Lemma 2.1, we see that

111 $$\begin{aligned} \left| \partial _x[\phi _\epsilon ' v_\epsilon '(v_\epsilon -1)]\right| \le \frac{C}{\epsilon }(v_\epsilon -1)^\gamma . \end{aligned}$$

Hence

$$\begin{aligned} |K_6| \le \frac{C}{\epsilon ^{2}}\int _{0}^{t} D_{1}(\tau ;V )~d\tau + \frac{C}{\epsilon ^{4}} \int _{0}^{t}D_{0}(\tau ;V )~d\tau . \end{aligned}$$

To estimate $K_{7}$, we first integrate by parts to obtain

$$\begin{aligned} \begin{aligned} K_{7} =&- \int _{0}^{t} \int _{\mathbb {R}} \frac{\eta \partial _{x}^{2}\eta }{v_{\epsilon }-1} \partial _{x}[\phi _\epsilon ' v_\epsilon '(v_\epsilon -1)] + \int _{0}^{t} \int _{\mathbb {R}} \frac{\eta \partial _{x}\eta }{(v_{\epsilon }-1)^{2}} v_{\epsilon }' \partial _{x}[\phi _\epsilon ' v_\epsilon '(v_\epsilon -1)]. \end{aligned} \end{aligned}$$

Then using again (111) and the Holder and Young inequalities,

112 $$\begin{aligned} \begin{aligned} |K_{7}|&\le \frac{C}{\epsilon }\Vert \sqrt{\phi _{\epsilon }}\partial _{x}V \Vert _{L^{2}_{t,x}} \left( \left\| \frac{(v_\epsilon -1)^{\gamma -2}}{\phi _\epsilon }\right\| _{L^\infty _{t,x}}\Vert \sqrt{\phi _{\epsilon }}\partial _{x}^{2}\eta \Vert _{L^{2}_{t,x}} + \left\| \frac{v_\epsilon '(v_\epsilon -1)^{\gamma -3}}{\phi _\epsilon }\right\| _{L^\infty _{t,x}} \Vert \sqrt{\phi _{\epsilon }}\partial _{x}\eta \Vert _{L^{2}_{t,x}}\right) \\&\le \frac{C}{\epsilon ^{2}}\int _{0}^{t} D_{1}(\tau ;V )~d\tau + \frac{C}{\alpha \epsilon ^{4}} \int _{0}^{t}D_{0}(\tau ;V )~d\tau + \frac{\alpha }{6} \int _{0}^{t} D_{2}(\tau ;V )~d\tau . \end{aligned} \end{aligned}$$

Summing $|K_1|,\dots , |K_7|$ finally yields the desired estimate. $\square$
